# Design, Synthesis, Electrochemical, and Biological Evaluation of Fluorescent Chlorido[*N*,*N*′-bis(methoxy/hydroxy)salicylidene-1,2-bis(4-methoxyphenyl)ethylenediamine]iron(III) Complexes as Anticancer Agents

**DOI:** 10.1021/acs.jmedchem.3c01359

**Published:** 2023-11-28

**Authors:** Astrid Dagmar Bernkop-Schnürch, Donja Chavooshi, Hubert Aaron Descher, Daniel Leitner, Heribert Talasz, Martin Hermann, Klaus Wurst, Stephan Hohloch, Ronald Gust, Brigitte Kircher

**Affiliations:** † Department of Pharmaceutical Chemistry, Institute of Pharmacy, CMBI−Center for Molecular Biosciences Innsbruck, CCB—Center for Chemistry and Biomedicine, University of Innsbruck, Innrain 80-82, 6020 Innsbruck, Austria; ‡ Immunobiology and Stem Cell Laboratory, Department of Internal Medicine V (Hematology and Oncology), Medical University of Innsbruck, Anichstraße 35, 6020 Innsbruck, Austria; § Department of General, Inorganic and Theoretical Chemistry, University of Innsbruck, Innrain 80-82, 6020 Innsbruck, Austria; ∥ Biocenter, Institute of Medical Biochemistry, Protein Core Facility, Medical University of Innsbruck, Innrain 80-82, 6020 Innsbruck, Austria; ⊥ Department of Anesthesiology and Critical Care Medicine, Medical University of Innsbruck, Anichstraße 35, 6020 Innsbruck, Austria; # Tyrolean Cancer Research Institute, Innrain 66, 6020 Innsbruck, Austria

## Abstract

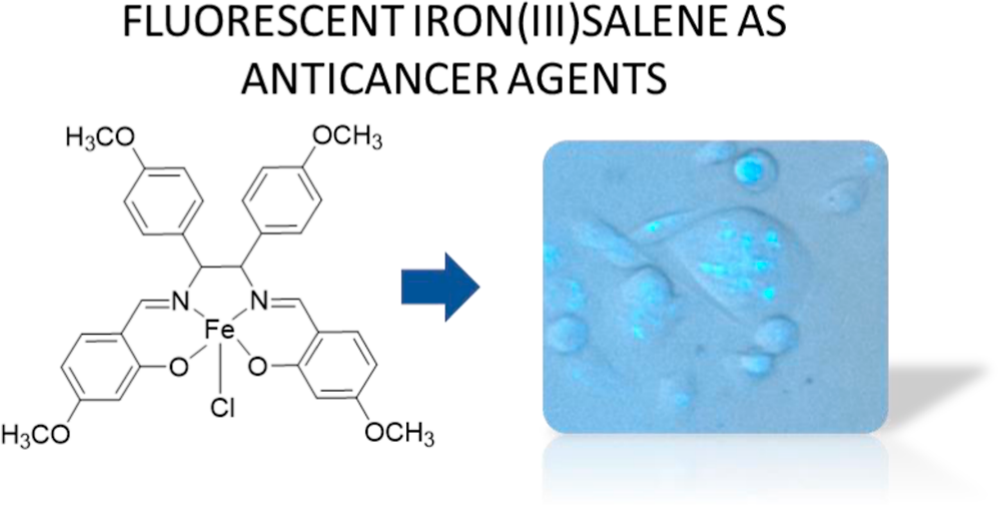

The impact of methoxy and hydroxyl groups at the salicylidene moiety of chlorido[*N*,*N*′-bis(methoxy/hydroxy)salicylidene-1,2-bis(4-methoxyphenyl)ethylenediamine]iron(III) complexes was evaluated on human MDA-MB 231 breast cancer and HL-60 leukemia cells. Methoxylated complexes (**C1**–**C3**) inhibited proliferation, migration, and metabolic activity in a concentration-dependent manner following the rank order: **C2** > **C3** > **C1**. In particular, **C2** was highly cytotoxic with an IC_50_ of 4.2 μM which was 6.6-fold lower than that of cisplatin (IC_50_ of 27.9 μM). In contrast, hydroxylated complexes **C4**–**C6** were almost inactive up to the highest concentration tested due to lack of cellular uptake. **C2** caused a dual mode of cell death, ferroptosis, and necroptosis, whereby at higher concentrations, ferroptosis was the preferred form. Ferroptotic morphology and the presence of ferrous iron and lipid reactive oxygen species proved the involvement of ferroptosis. **C2** was identified as a promising lead compound for the design of drug candidates inducing ferroptosis.

## 1 Introduction

The therapeutic use of metal complexes in cancer treatment has a long history.^1−4^ Among various metals including platinum^5−8^ (which is broadly used in the form of cisplatin^6^), gold^9^ and copper,^10^ more recently iron moved into the limelight of research.^11−16^ On the one hand, iron is essential for cells by playing a crucial role in, e.g., electron transfer as well as oxygen transport and storage.^17^ On the other hand, it is toxic by forming hydroxyl radicals. The underlying mechanism is known as the Fenton reaction where iron ions and peroxides react to hydroxyl radicals^18^ as following: Fe^2+^ + H_2_O_2_ → Fe^3+^ + OH^–^ + OH^•^. Mediated by this reaction, lipids are peroxidized, and the resulting hydroperoxides destroy membrane structures causing a nonapoptotic cell death–ferroptosis.^19,20^ As ferroptosis plays a critical role in cancer,^21−24^ research on ferroptosis inducing active pharmaceutical ingredients as a new generation of anticancer drugs seems promising. Our research group has shown that not only iron ions but also chlorido [*N*,*N*′-bissalicylidene-1,2-phenylenediamine]iron(III) complexes induce ferroptosis, underlining their cytotoxic potential on cancer cells.^11,25^ Nevertheless, these drug candidates have not reached their full potential yet by far. In particular, the exchange of the 1,2-phenylenediamine by a 1,2-bis(4-methoxyphenyl)ethylenediamine moiety, which is known to act as a carrier for platinum(II) complexes,^26,27^ as well as the impact of ligands increasing the electron density on the salicylidene substructure, deserves detailed investigation and is addressed in this report.

**Scheme 1:**
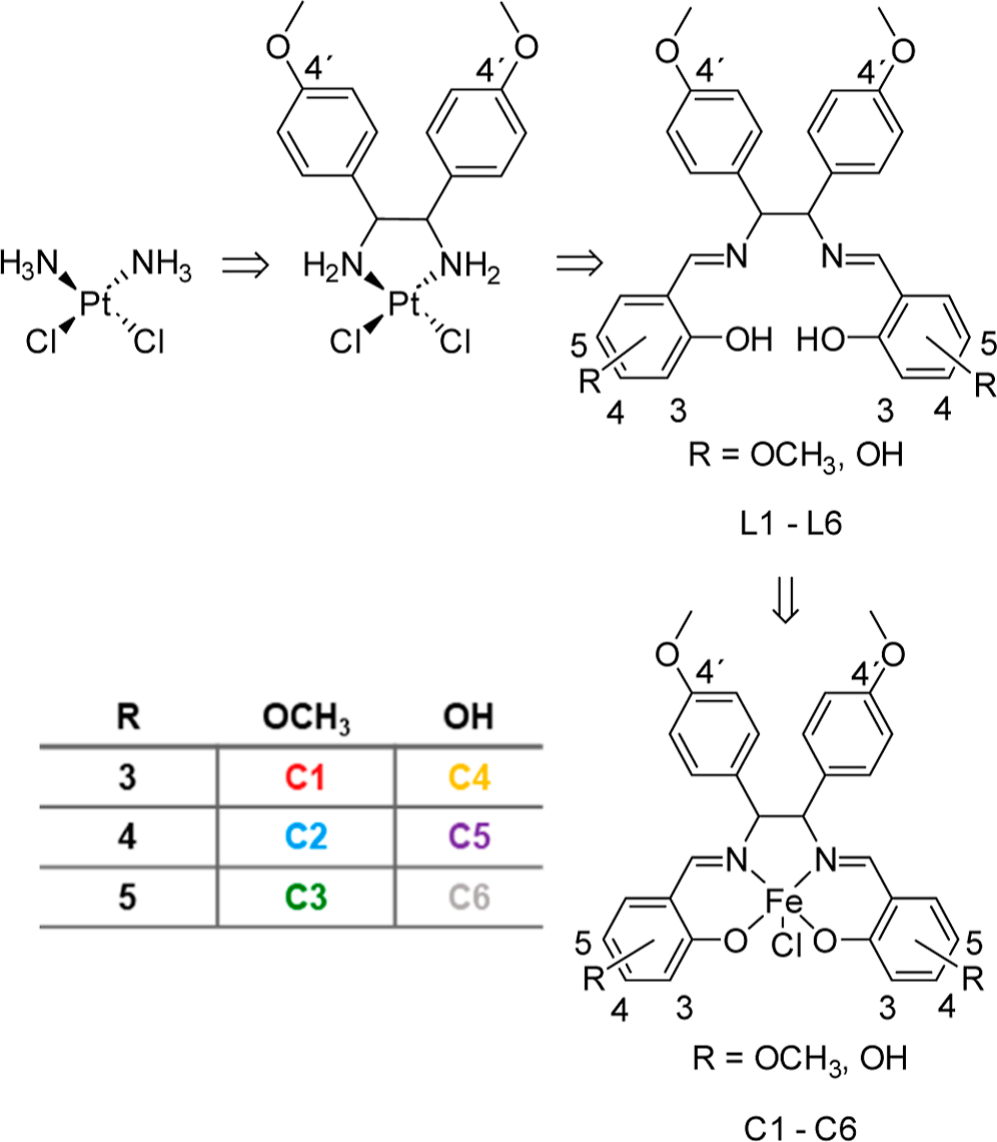
Design of Substituted Complexes **C1–C6** Starting from cisplatin, the diarylethane structure was introduced by substituting the diammine with an 1,2-bis(4-methoxyphenyl)ethylenediamine moiety. In parallel, the dichloridoplatinum substructure was removed, and bis-Schiff bases were formed (**L1**–**L6**) that chelate iron (**C1**–**C6**).

The design of these complexes (**C1–C6**) is outlined in Scheme 1.

The diammine substructure of cisplatin was replaced by a 1,2-bis(4-methoxyphenyl)ethylenediamine moiety. In parallel, the dichloridoplatinum substructure was removed, and bis-Schiff bases were formed between the primary amino groups and methoxylated or hydroxylated salicylaldehyde, resulting in the bis(salicylidene)ethylenediamine (salene) scaffold (**L1**–**L6**, Figures S1–S6) that chelate iron.

Chlorido[*N*,*N*′-bis (methoxy/hydroxy)salicylidene-1,2-bis(4-methoxyphenyl)ethylenediamine]iron(III) complexes **C1–C6** were chemically characterized, their fluorescence intensity was analyzed, and their redox behavior was evaluated via cyclic voltammetry. Moreover, the structure–activity relationship (SAR) of these complexes was investigated by means of cellular proliferation and cytotoxicity as well as cellular uptake studies using atom absorption spectroscopy and live confocal microscopy.

## 2 Results and Discussion

### 2.1 Chemistry

#### 2.1.1 Synthesis and Structural Characterization

The synthesis of complexes **C1–C6** follows a multistep process, according to a known procedure with minor modifications.^12,13^ All complexes (**C1–C6**) were obtained as a black powder. The coordination of the ligands to iron(III) was confirmed by Fourier transform infrared (FT-IR) spectroscopy (Figures S7–S12) and mass spectrometry. Elemental analysis (CHN) confirmed the elemental composition and purity. Purity of the lead compounds **C2** and **C6** was further analyzed by high-performance liquid chromatography (HPLC, Figures S13 and S14). Due to the paramagnetic nature of the complexes, effective magnetic moments were determined using the Evans method.^28^ Measured magnetic moments ranged from 5.17 to 5.57 μ_B_ for **C1**–**C6** (Figures S15–S26), indicating the formation of high-spin Fe(III) complexes (*S* = 5/2), which is in accordance with other iron(III) complexes reported.^29,30^ In addition, electron paramagnetic resonance (EPR) measurements revealed the expected *g*-values and signature for an iron(III) center (Figures S27 and S28). Furthermore, crystal structure of **C2** and **C4** was determined (Tables S1, S2, Figures S29 and S30). Additional details of the crystal structure investigation can be obtained from the Cambridge Crystallographic Data Centre (CCDC).

#### 2.1.2 Evaluation of Fluorescence Properties

**Figure 1:**
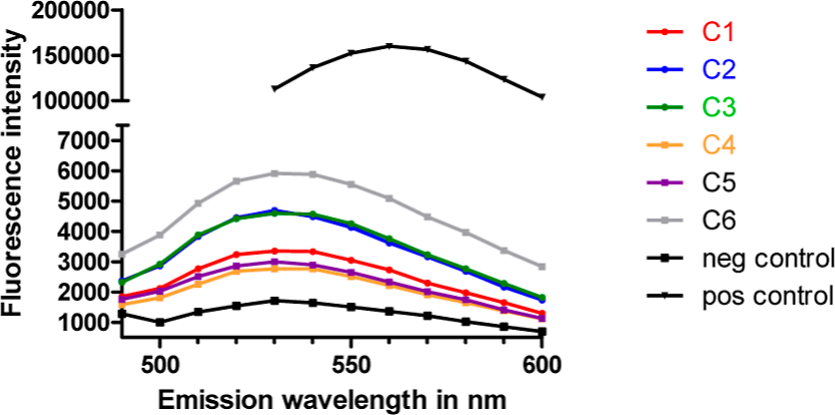
Fluorescence evaluation of complexes **C1**–**C6**, coumarin 6 (positive control), and chlorido[*N*,*N*′-disalicylidene-1,2-phenylenediamine]iron(III) (negative control).

Although various Schiff bases have been reported as fluorescent when complexed with metal ions,^31−34^ the iron complexes synthesized so far did not show fluorescence. With the introduction of methoxy and hydroxyl groups in the salicylidene moiety of our chlorido[*N*,*N*′-bissalicylidene-1,2-bis(4-methoxyphenyl)ethylenediamine]iron(III) complexes, the electron density on the ring system can be increased. This influences the electronic properties of the molecule, leading to changes in the wavelength and fluorescence behavior. As fluorescent compounds are a powerful analytical tool to investigate their fate in cancer cells in vitro and, subsequently, in vivo, we evaluated the fluorescence of these complexes. Therefore, **C1**–**C6** were dissolved in dimethyl sulfoxide (DMSO) (5 mM) and diluted with cell culture medium without fetal bovine serum (FBS) (ratio 1:9), transferred into a 96-well plate, and their fluorescence was recorded by an excitation wavelength of 450 nm and an emission scan in the range of 490–600 nm using a fluorimeter. As a positive control served the well-known fluorescence dye coumarine 6 and, as a negative control, chlorido[*N*,*N*′-disalicylidene-1,2-phenylenediamine]iron(III) (Figure 1).

As anticipated, coumarin 6 displayed a pronounced fluorescence at an emission (*E*_m_) maximum of 560 nm. For all iron complexes, *E*_m_ maxima were detected at 530 nm. Among the tested iron complexes, **C2**, **C3**, and **C6** exhibited the highest fluorescence. As expected, the negative control showed a minor fluorescence.

#### 2.1.3 Cyclic Voltammetry

**Figure 2:**
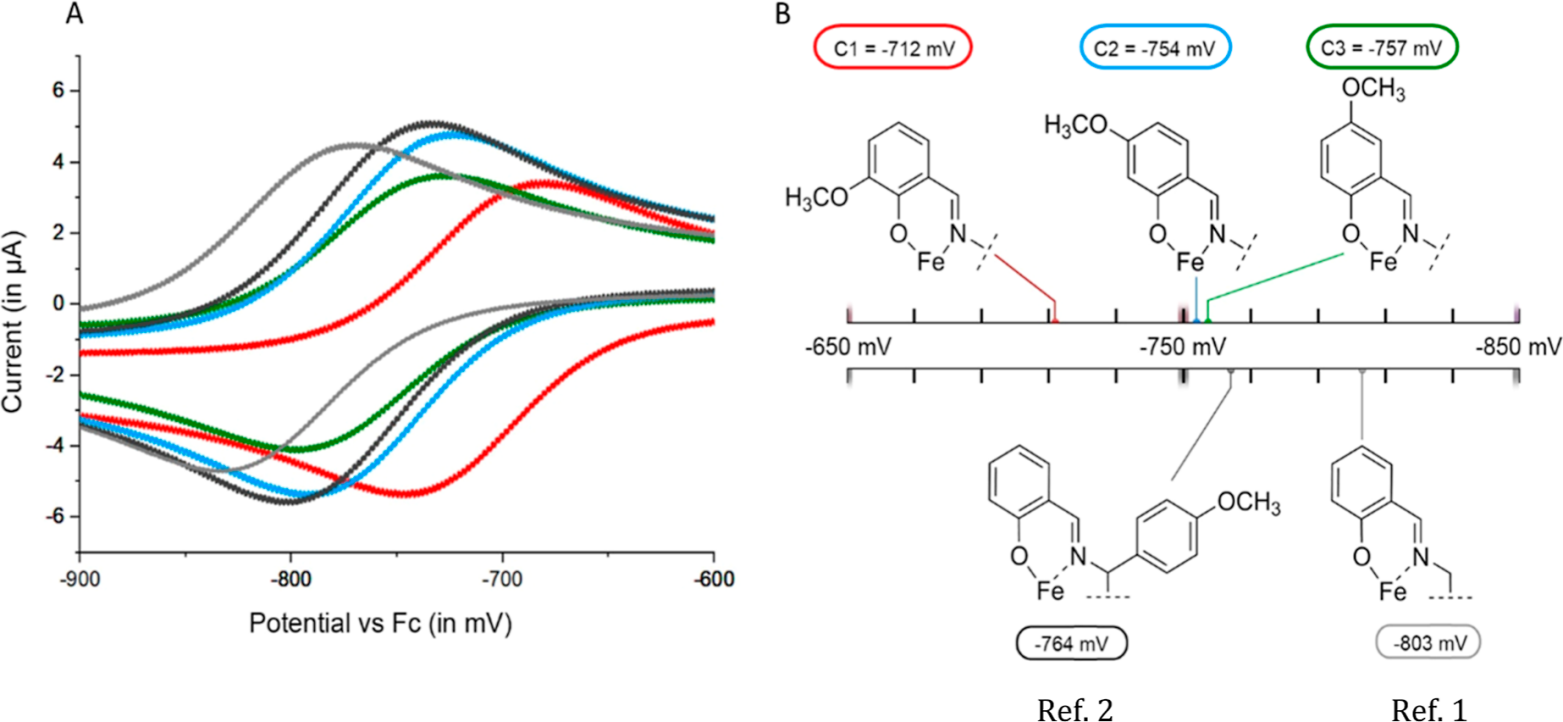
Graphical presentation of the redox potentials of **C1**–**C3** by combining the resulting voltammograms (A) and comparison of the values of the redox potentials of **C1** (red), **C2** (blue), **C3** (green), ref (1) (light gray), and ref (2) (dark gray) in DMSO (B).

Iron complexes are redox active and have the ability to form iron (III/II) pairs^35^ that may participate in the Fenton reaction. Therefore, the electrochemical behavior was analyzed by cyclic voltammetry in DMSO, the solvent of the complexes. The voltammograms versus ferrocene (Fc) and the standard potential of the iron(III/II) redox pairs of **C1–C3** as well as (bissalicylidenethylenediamine)iron(III) (ref (1)) and the unsubstituted chlorido[*N*,*N*′-bissalicylidene-1,2-bis(4-methoxyphenyl)ethylenediamine]iron(III) (ref (2)) to evaluate the influence of the 1,2-bis(4-methoxyphenyl)-substitution at the ethylenediamine part and the methoxy substitution in the salicylidene moieties are depicted in Figure 2A. Data of **C4–C6** are presented as Supporting Information (Figure S31 and Table S3).

Comparison of refs (1) and (2) indicated that the additional methoxy groups on the ethylenediamine substructure decreased the electron density of the iron metal center (Figure 2B). In addition, the substitution in the salicylidene moiety clearly influenced the redox potential in dependence of the group and its position. The methoxy group increased the redox potential in the following order: **C1** (*E*_Δ_ = 52 mV) > **C2** (*E*_Δ_ = 10 mV) = **C3** (*E*_Δ_ = 7 mV). Although the methoxy groups generally exhibit a +M effect,^36,37^ the −I effect seems to dominate, likely influenced through the iron center compared to the unsubstituted derivative ref (2). With *E* = −694 mV, **C4** showed a significant decrease in the electron density of 70 mV compared to *E* = −764 mV of ref (2), which is likely the result of the formation of hydrogen bonds. Furthermore, **C5** decreased the redox potential of the iron(III/II) pair to −794 mV, which can be explained by the electron-donating properties of hydroxyl groups. Especially, the comparison of **C4** and **C5** highlights the influence of the substitution pattern on the redox potential. Comparison of **C2** (*E* = −754 mV), which is slightly higher and **C5** (*E* = −794 mV), that has a lower redox potential than ref (2), shows the different effects of the group on the iron(III/II) redox couple. The standard potential of **C6** was found at about *E* = −333 mV. Although the comparison of reduction and oxidation peak current did not significantly differ, the peak-to-peak separation of the reduction and oxidation signal at about 202 mV revealed that this redox pair was quasi-reversible. The signal could be assigned to the reduction and oxidation of the unchelated iron(III/II),^38^ indicating that this complex is not stable in the electrochemical cell. Nevertheless, the electrochemical behavior indicates that these complexes can participate in the Fenton reaction.

### 2.2 Biological Activity

The anticancer activity of the complexes was analyzed by their capacity to inhibit cell growth and migration as well as to induce cell death. The biological effects were tested on the ferroptosis sensitive mammary carcinoma cell line MDA-MB 231^39^ and the acute myeloid leukemia cell line HL-60.^40^

#### 2.2.1 Effect on Cell Growth

The antiproliferative activity was investigated by the ability of the complexes to inhibit [^3^H]-thymidine incorporation into DNA. The cell lines were incubated for 72 h with complexes **C1–C6** and cisplatin as a reference at concentrations ranging from 1 to 20 μM.

**Figure 3:**
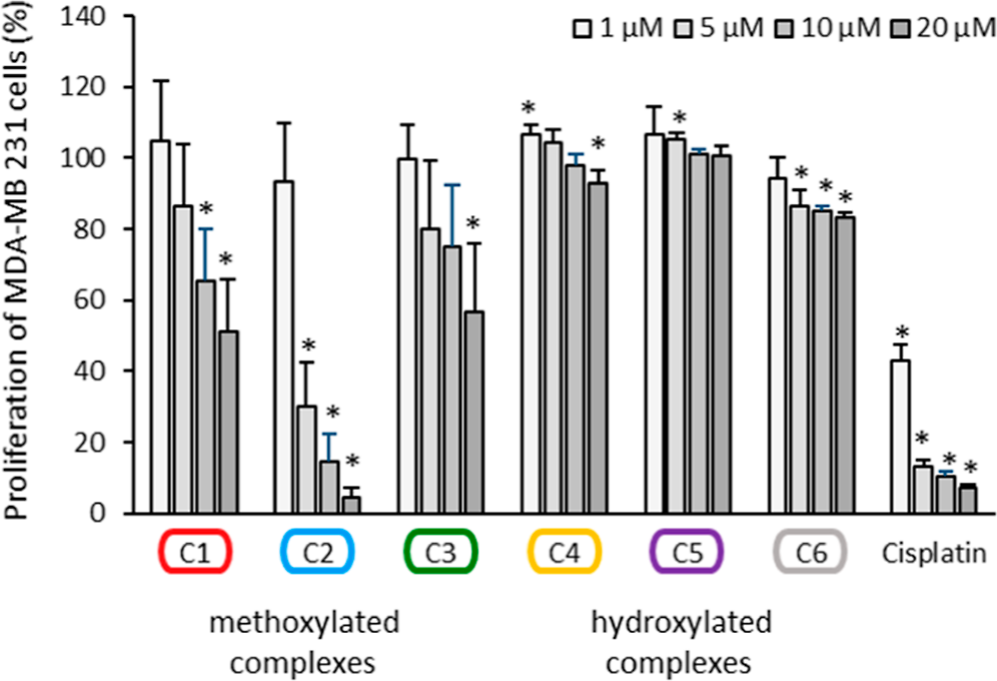
Proliferation of MDA-MB 231 cells treated for 72 h with the complexes **C1–C6** and cisplatin at concentrations 1, 5, 10, and 20 μM, respectively. Proliferation in the absence of the compounds was set at 100% (data not shown). Data are expressed as mean + SE of five experiments. The asterisk (*p < 0.05 against no compound) represents statistical significance.

The methoxylated complexes **C1–C3** inhibited proliferation of the cell lines MDA-MB 231 and HL-60 in a concentration-dependent manner, whereby mammary carcinoma cells were slightly more sensitive than leukemic cells (Figures 3 and S32).

The strongest antiproliferative effect was observed for the methoxylated complex **C2** that significantly reduced the proliferation at 20 μM to 4.3 ± 3.0%, similar to cisplatin (7.2 ± 0.9%), indicating an IC_50_ of 1.1 μM for **C2** in comparison to an IC_50_ of 0.4 μM for cisplatin. **C1** was similar active than **C3** with a proliferation rate of 51.3 ± 14.7% versus 56.8 ± 19.1% at 20 μM, respectively. In contrast, hydroxylated complexes **C4–C6** did not strongly influence proliferation of MDA-MB 231 and HL-60 cells at the applied concentrations. Although statistically significant (due to the low standard error), the proliferation did not fall below 83.1 ± 1.5% (**C6**) at the highest concentration tested.

#### 2.2.2 Effect on Cell Death

##### 2.2.2.1 Effect on Metabolic Activity

A modified 3-(4,5-dimethylthiazol-2-yl)-2,5-diphenyl-tetrazolium bromide (MTT) assay, which detects the reduction of light-yellow tetrazolium salts into orange formazan derivatives by functional mitochondria, was performed to study the cytotoxic activity of the complexes.

**Figure 4:**
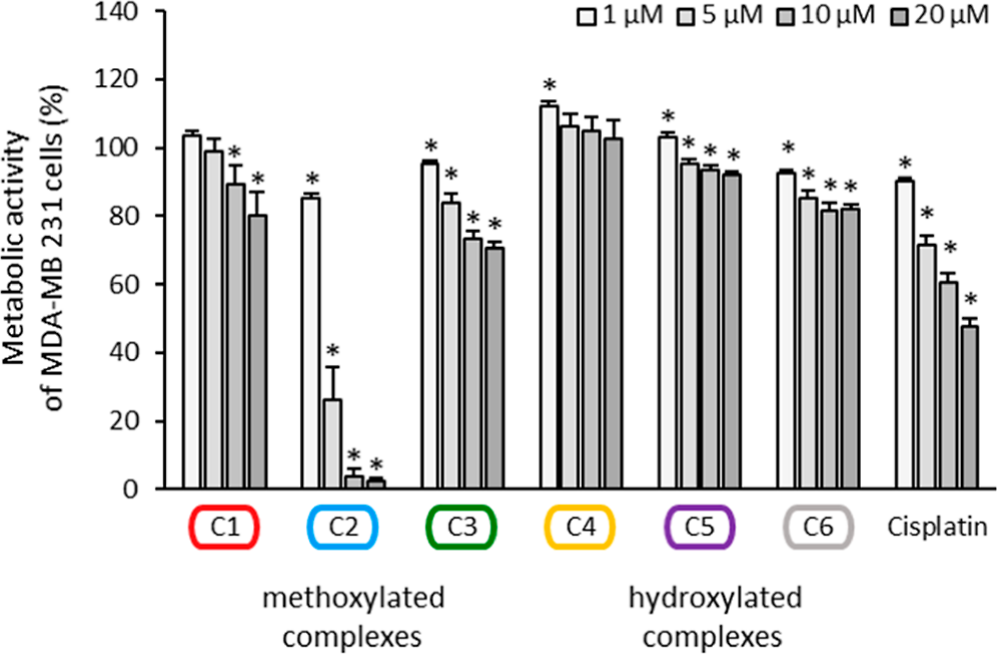
Metabolic activity of MDA-MB 231 cells treated for 72 h with the complexes **C1–C6** and cisplatin at concentrations 1, 5, 10, and 20 μM, respectively. Metabolic activity in the absence of the complexes was set at 100% (data not shown). Data are expressed as mean + SE of five experiments. The asterisks represent statistical significance.

All compounds, as well as cisplatin, concentration-dependently and statistically significant inhibited the metabolic activity, whereby their antimetabolic activity was not as strong as their antiproliferative activity, indicating stronger cytostatic than cytotoxic effects (Figures 4 and S33).

Again, **C2** was the most effective complex, significantly decreasing the metabolic activity at 5 μM to 26.2 ± 9.7% and at 20 μM to 2.4 ± 1.1%. An IC_50_ of 4.2 μM that was 6.6-fold lower than that of cisplatin (IC_50_ of 27.9 μM) was calculated. From the hydroxylated series, **C6** again reduced the metabolic activity more significantly than its counterparts did. However, even at 20 μM **C6** statistically, but of negligible significance, diminished the metabolic activity of MDA-MB 231 cells to 82.0 ± 1.5%.

As the redox potentials of **C2** and **C3** are very similar but metabolic activity is clearly different, no correlation between biological activity and redox behavior was identified.

##### 2.2.2.2 Effect on Migration

The inhibitory effect of the compounds was further tested by the “scratch” assay, also known as a wound healing assay. The scratch assay is a useful tool for studying cancer-cell migration and invasion, which are key processes in tumor progression and metastasis.

In this analysis, the confluent monolayer was scratched with a pipet tip, and the migratory potential of the compounds to close this scratch was analyzed microscopically by taking pictures immediately after complex addition and every 24 h.

MDA-MB 231 cells in the absence of the complex migrated toward the scratch very quickly and closed it completely within 72 h (Figure S34A). In the presence of 20 μM **C2**, some living MDA-MB 231 cells migrated toward the scratch, but cell death—indicated by detached, round cells—was already visible after 24 h (Figure S34B), thus confirming antiproliferative activity. After 72 h, no living cells were detected anymore, as also observed in the metabolic activity assay. In contrast, the corresponding hydroxylated compound **C5** that did not inhibit proliferation migrated similar to the negative control (Figure S34C).

##### 2.2.2.3 Mode of Cell Death

**Figure 5:**
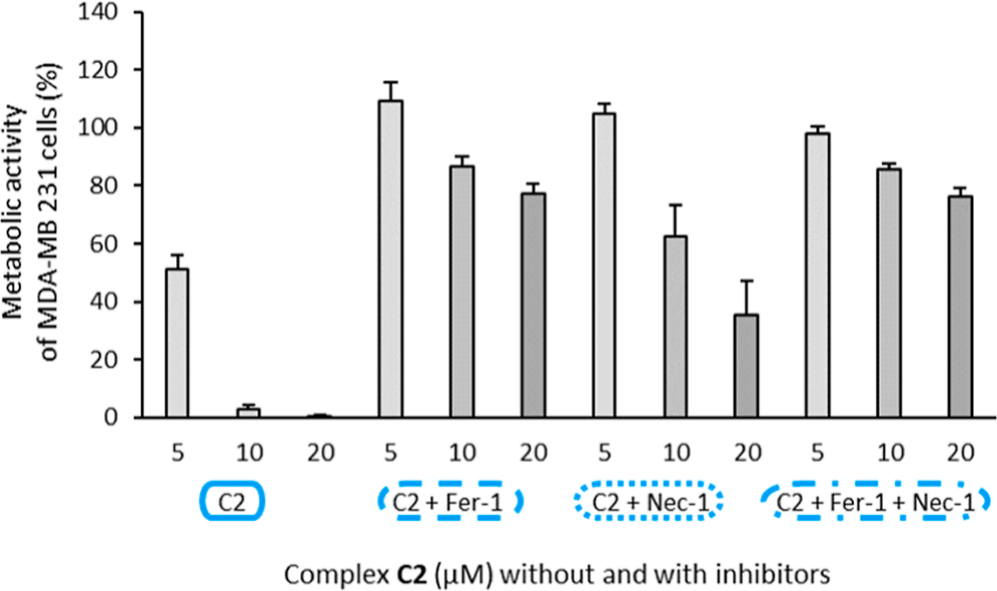
Metabolic activity of MDA-MB 231 cells treated for 72 h with **C2** at concentrations 5, 10, and 20 μM, respectively, alone and in combination with the cell death inhibitors Fer-1 (1 μM) and/or Nec-1 (20 μM). Metabolic activity in the absence of **C2** was set at 100% (data not shown). Data are expressed as the mean + SE of four experiments.

Since **C2** was the strongest inhibitory complex in this series, its mode of cell death was investigated in more detail. **C2** was incubated simultaneously with an inhibitor of ferroptosis (ferrostatin-1, Fer-1, 1 μM) and/or an inhibitor of necroptosis (necrostatin-1, Nec-1, 20 μM) for 72 h, and metabolic activity was used as read-out. Fer-1 was able to completely prevent the inhibition of metabolic activity induced by 5 μM **C2** from 51.2 ± 4.8 to 109.2 ± 6.3% and by 20 μM **C2** from 10.5 ± 0.4 to 77.2 ± 3.7% (Figure 5). Nec-1 was able to compensate the inhibitory effect of 5 μM **C2** (to 104.9 ± 3.4%), but was less effective at 10 and 20 μM **C2**, respectively, and did not further enhance the abrogating effect of Fer-1. Thus, at higher concentrations, ferroptosis was the preferred mode of cell death.

#### 2.2.3 Cellular Uptake

##### 2.2.3.1 Quantification of Iron by Graphite Furnace Atomic Absorption Spectrometry

The lacking biological activity of the hydroxylated complexes might be explained by their insufficient membrane permeability in contrast to that of the methoxylated complexes. In order to confirm this working hypothesis, a cellular uptake study using GF-AAS was performed.

Therefore, MDA-MB 231 cells were incubated with 5 μM **C2** and **C6**, respectively, at various time points (20 min, 1, 4, and 24 h). To overcome the potential problem that ubiquitous presence of iron in cells may influence results, cells incubated without a compound were prepared under the same conditions and were used as a negative control.

**Figure 6:**
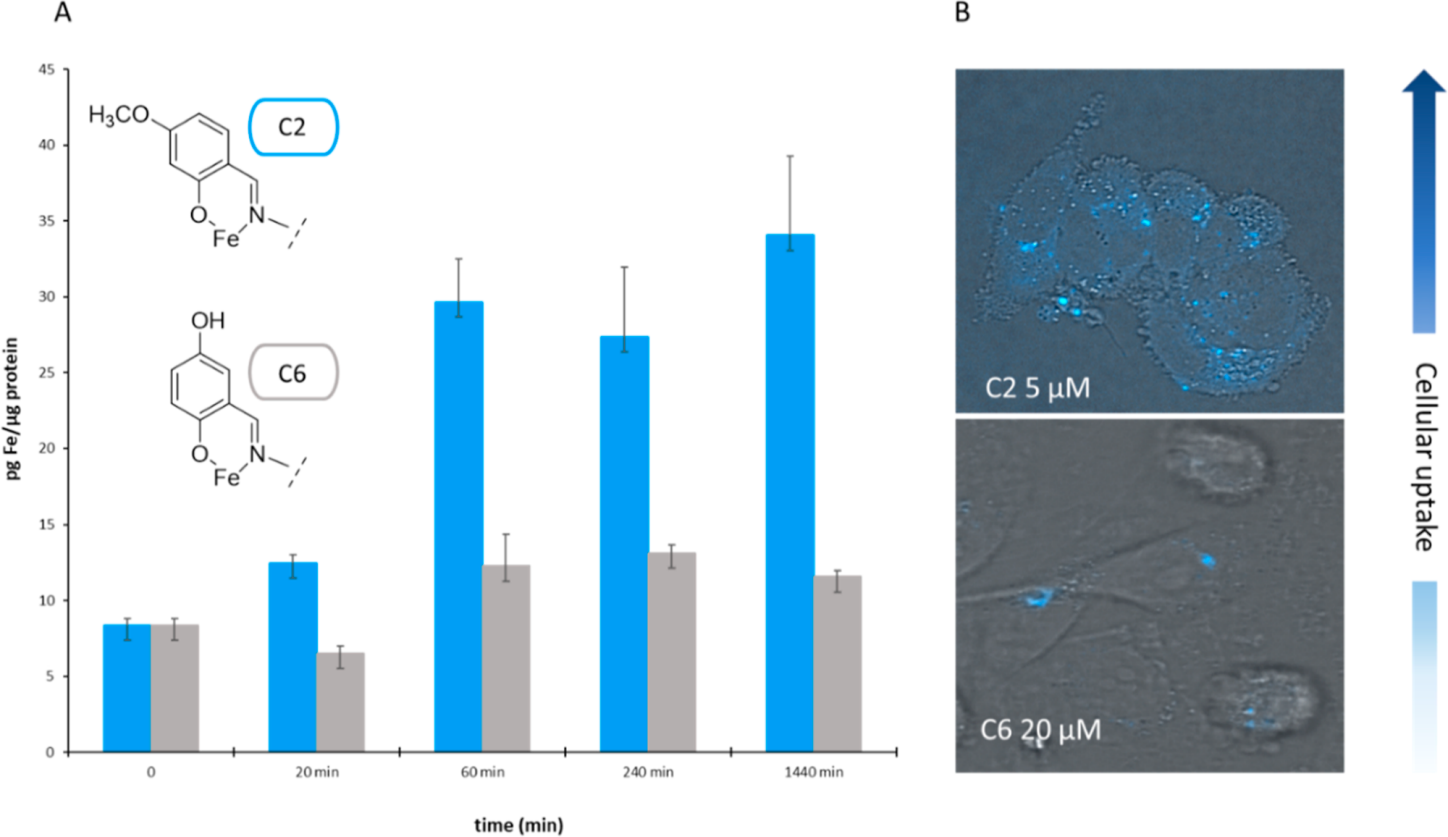
(A) Cellular uptake measured via GF-AAS after 0 min, 20 min, 1, 4, and 24 h incubation in MDA-MB 231 cells with complex **C2** and **C6** at 5 μM, respectively. Cells without complex served as reference. Data are expressed as the mean + SE of three experiments. (B) Cellular uptake was visualized via live confocal microscopy at 405 nm in MDA-MB 231 cells with complex **C2** 5 μM and **C6** 20 μM.

**C2** accumulated in the breast cancer cell line with a steep increase already after 60 min to 29.7 ± 2.8 pg Fe/μg protein which is even slightly higher after 24 h (34.1 ± 5.2 pg Fe/μg protein). In contrast, despite addition of **C6**, almost the same amount of iron was measured compared to the negative control (Figure 6A).

As a lower amount of iron was detected in MDA-MB 231 cells treated with complex **C6**, the limited biological activity of this complex could likely be explained by the differences in the cellular uptake of **C2** versus **C6**.

Furthermore, these findings highlight that not only the 1,2-bis(4-methoxyphenyl)ethylenediamine moiety is crucial for cellular uptake but also the substructure on the salicylidene moiety.

##### 2.2.3.2 Imaging Cellular Uptake

**C2** and **C6** were selected due to their strong fluorescence properties to investigate the cellular uptake via a live confocal microscopy study. After a 24 h incubation with 5 μM **C2**, fluorescence was detected in MDA-MB 231 cells, confirming the cellular uptake (Figure 6B). Furthermore, cells treated with **C2** showed characteristics of ferroptotic cell death,^19^ which is in accordance with the inhibitor experiments. In contrast, despite a 24 h treatment with **C6** at 20 μM, only living cells with characteristic epithelial appearance and fluorescence outside the cells were detected, concluding that the lack of effectiveness of the complexes **C4**–**C6** results from lack of the cellular uptake.

#### 2.2.4 Study of Ferroptotic Activity

##### 2.2.4.1 Morphology and Ferrous Iron

**Figure 7:**
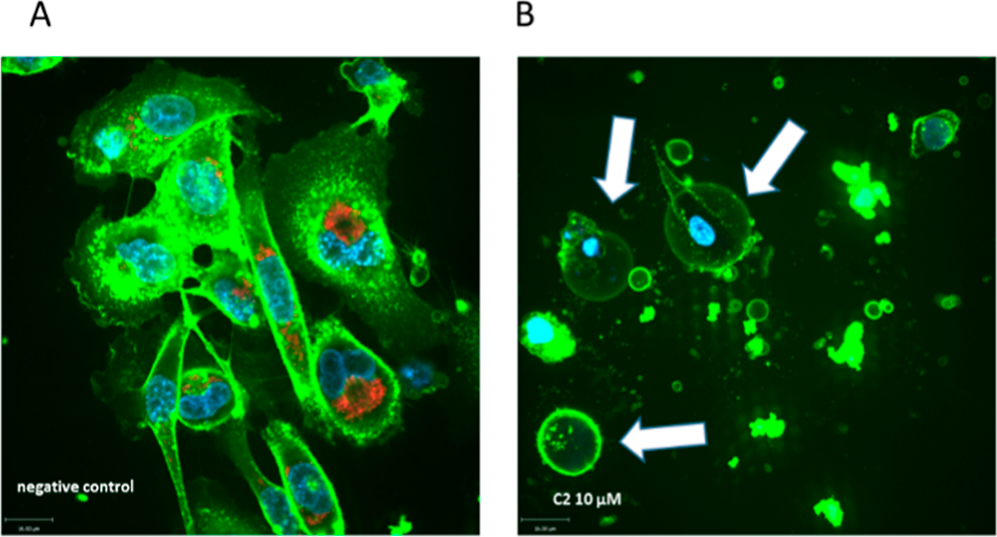
MDA-MB 231 cells imaged by live confocal microscopy after staining with Hoechst 33342 (blue, 405 nm) to image the nuclei, wheat germ lectin (green, 488 nm) to visualize the cell morphology, and TMRM (red, 561 nm) to stain active mitochondria. (A) untreated cells; (B) incubation with 10 μM **C2** for 24 h. One representative experiment is shown.

Live confocal microscopy was also used to detect the ferroptotic activity of the complexes. MDA-MB 231 cells were incubated for 24 h with 1 and 10 μM **C2** and 10 μM **C6**, respectively, and their effect on active mitochondria was visualized by tetramethylrhodamine ethyl ester (TMRM), a red cell-permeant fluorescent dye (Figures 7 and S35).

The pictures demonstrate the changes in the cell morphology after incubation with **C2** (Figures 7B and S35A) clearly visible by the concentration-dependent change from the typical spindle-shape MDA-MB 231 cells to round-shaped ferroptotic cells (white arrows in Figure 7B). This clearly indicates an uptake of the substance and confirms the high cytotoxic activity of **C2**. In contrast, when cells were treated with **C6** (Figure S35B), no difference to the negative control (Figure 7A) was detected.

**Figure 8:**
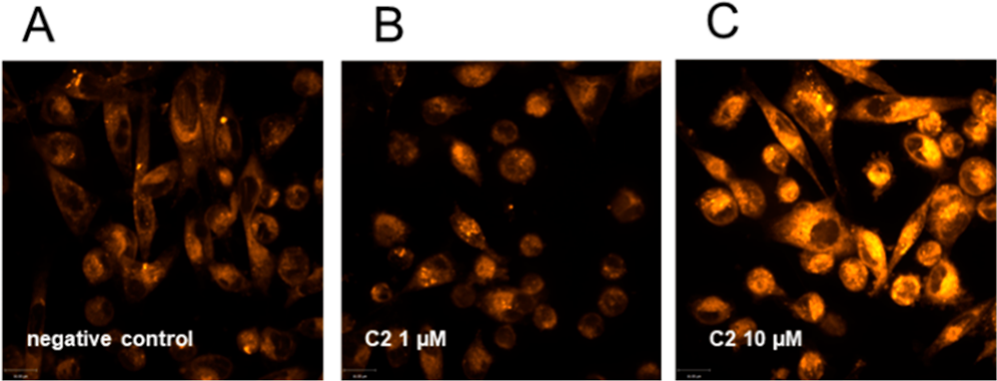
MDA-MB 231 cells imaged by live confocal microscopy after staining with FerroOrange (orange, 561 nm) to visualize ferrous iron (A) untreated cells; (B) incubation with 1 μM **C2**; and (C) incubation with 10 μM **C2** for 24 h. One representative experiment is shown.

Live confocal imaging was again utilized to further study the ferroptotic activity using FerroOrange staining.^41^ This dye uniquely detects the labile ferrous form of iron, which can participate in the Fenton reaction and drives ferroptosis. Figure 8A depicts the presence of a physiological amount of ferrous iron in MDA-MB 231 cells. After treatment with 1 μM **C2** a slight increase of ferrous iron is visible (Figure 8B). **C2** at 10 μM clearly increased the intensity of the dye, indicating high amounts of iron within the cells.

##### 2.2.4.2 Lipid Peroxidation

Ferroptosis is characterized by the accumulation of lipid peroxides, resulting from lipid reactive oxygen species (ROS) generated through the Fenton reaction. To further investigate the data indicating that ferroptosis is the main cause of cell death by **C2**, lipid ROS was detected with BODIPY 581/591 C11 dye staining and analyzed by flow cytometry.^42^ MDA-MB 231 cells were treated with 10 and 20 μM **C2** for 2, 4, 16, and 24 h, respectively (Table 1). After 2 and 4 h, a 12- to 14-fold induction of lipid ROS was detected with both concentrations, whereas a strong drop to background levels was observed after 16 and 24 h.

**Table 1:** *x*-Fold Induction of MDA-MB 231 Cells with Lipid Peroxidation after Treatment with **C2** at 10 and 20 μM for 2, 4, 16, and 24 h, Respectively^a^

*x*-fold induction	2 h	4 h	16 h	24 h
10 μM **C2**	12	14	3	1
20 μM **C2**	13	12	2	1

a One representative experiment is depicted.

**Figure 9:**
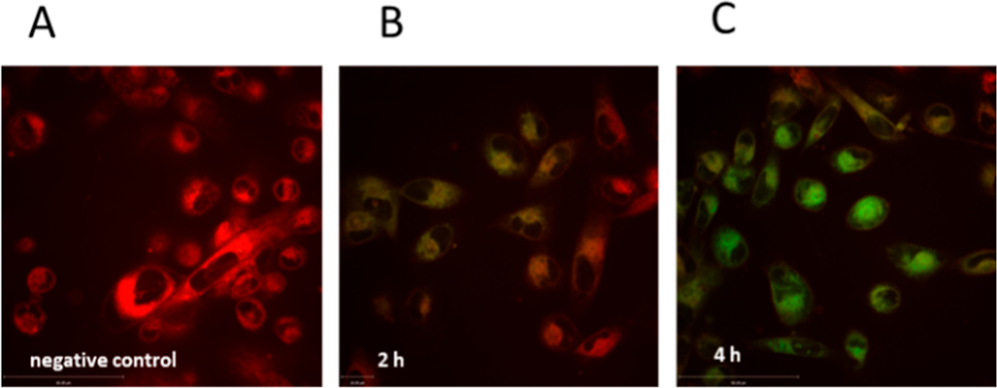
MDA-MB 231 cells imaged by live confocal microscopy after staining with BODIPY to visualize lipid peroxidation (A) untreated cells; (B) incubation with 10 μM **C2**; and (C) incubation with 10 μM **C2** for 4 h. One representative experiment is shown.

In addition, lipid peroxidation was visualized by live confocal microscopy utilizing also BODIPY 581/591 C11 dye. Due to lipid ROS formation, the fluorescence of this fluorophore shifts from red (unoxidized) to green (oxidized). The negative control (MDA-MB 231 without treatment) represents the red-colored BODIPY (Figure 9A). After 2 h **C2** treatment, cells exhibited additionally green fluorescence representing lipid ROS (Figure 9B), which was even more enhanced after 4 h **C2** incubation (Figure 9C). This indicates that **C2** caused strong lipid peroxidation that can terminate in ferroptosis.

## 3 Conclusions

Here, we report the design, synthesis, electrochemical, and biological evaluation of fluorescent chlorido[*N*,*N*′-bis(methoxy/hydroxy)salicylidene-1,2-bis(4-methoxyphenyl)ethylenediamine]iron(III) complexes. Cyclic voltammetry demonstrated the electrochemical potential of these complexes to participate in redox active reactions with the exception of **C6**, which was not stable under the conditions for electrochemical evaluation.

The complexes inhibited proliferation, migration, and metabolic activity. The methoxylated complexes were much more active than the hydroxylated ones. The efficacy was dependent on the position of the methoxy group and followed the rank order **C2** (position 4) > **C3** (position 5) > **C1** (position 3). The effectivity of **C2** to inhibit metabolic activity outperformed even cisplatin. In contrast, the complexes bearing a hydroxyl group were almost inactive in all assay systems tested. This missing biological activity was explained by their lack of cellular uptake, clearly demonstrated by GF-AAS and—due to their fluorescence—imaged via live confocal microscopy. Inhibition experiments with **C2** revealed that both ferroptosis and necroptosis are involved, whereby at higher complex concentrations, ferroptosis seems to be the main mode of cell death. The increased intracellular presence of ferrous iron and enhanced lipid peroxidation further proves this finding. In conclusion, **C2** was identified in this SAR study as a promising lead compound for the development of ferroptosis-inducing cancer drugs.

## 4 Experimental Section

### 4.1 General Materials and Instrumentation

Chemical reagents and solvents were purchased from commercial suppliers (Sigma-Aldrich, Fluka, Alfa Aesar, Cayman, and Acros) and used without further purification.

Analytical thin-layer chromatography was performed with Polygram SIL G/UV254 (Macherey-Nagel) plates (0.25 mm layer thickness) with a fluorescent indicator and Merck TLC Silica gel 60 F 254 aluminum backed plates. The spots were visualized with UV light (254 nm/365 nm).

NMR spectra were recorded on a Bruker Ultrashield 400 Plus spectrometer (^1^H NMR, 400 MHz; ^13^C NMR, 100 MHz). The centers of the solvent signal and the tetramethylsilane (TMS) signal were used as internal standards. Deuterated solvents used for the NMR spectra were purchased from Eurisotop. Chemical shifts are given in parts per million (ppm). Coupling constants are given in hertz (Hz). The following abbreviations are applied: s = singlet, d = doublet, dd = doublet of doublet, t = triplet, q = quartet, m = multiplet, and br = broad band shape.

Magnetic measurements of complexes **C1**–**C6** in solution were performed at room temperature by ^1^H NMR using the Evans method^28^ on a Bruker Avance 400 spectrometer operating at 400.14 MHz at a constant temperature of 298.15 K. The measurements of each compound were performed in standard 5 mm NMR tubes containing the paramagnetic samples dissolved in DMSO-*d*_6_ with an inert reference of 0.03% TMS, against a reference insert tube filled with the same solvent.

For mass spectra (MS), an Orbitrap Elite mass spectrometer (Thermo Fisher Scientific, Waltham, MA, USA) using direct infusion and electrospray ionization was used. MS data analysis was carried out using Xcalibur.

Elemental analysis on PerkinElmer Elementaranalysatoren 240 B and 240 C verified the ligands and the iron complexes.

HPLC: Shimadzu Nexera-i-LC-2040C-3D using a Kauner RP-18 end-capped 100–4.6 mm column. The purity of lead compounds was >99%.

FT-IR spectroscopy was recorded on a Bruker Alpha spectrometer with an ATR unit. FT-IR spectra were measured with 32 scans in the wavenumber range covering 4000–400 cm^–1^ and exerting a resolution of 1 cm^–1^. The following abbreviations are used for intensity specifications: w = weak, m = medium strong, s = strong, and br = broad band shape. The frequency (ν_max_) is given in cm^–1^.

Fluorescence was recorded with a Tecan Spark (Tecan, Grödig, Austria).

Cyclic voltammetry was performed with a BioLogic SP-150 as potentiostat. A conventional three electrode cell containing a platinum-wire counter-electrode, Ag/AgCl-electrode with saturated NaCl solution as a pseudo reference electrode, and a glassy carbon electrode as a working electrode was used. Ferrocene (2 mM) served as an internal standard. The supporting electrolyte Bu_4_NPF_6_ (TBAHPF, TCI) was utilized as received. EC-Lab V11.31 software was used to evaluate the data.

EPR spectra were recorded on a Magnettech 5000 X-band spectrometer in a frozen solution of DMSO in 3 mm o.d. fused silica tubes at 98 K.

GF-AAS analysis was performed with an M6 Zeeman GFAA-Spectrometer (Thermo Scientific).

Live confocal microscopy was performed with a Zeiss Axio Observer Z1 instrument (Zeiss, Oberkochen, Germany).

X-ray analysis for single crystals of **C2** and **C4** was performed with a Bruker D8 Quest diffractometer (Photon III C14). The instrument was equipped with an Incoatec Microfocus source generator (multi-layered optics monochromatized Mo Kα radiation, λ = 71.073 pm).

### 4.2 Chemistry

#### 4.2.1 General Procedure for the Synthesis of Ligands **L1**–**L6**

First, the meso 1,2-bis(4-methoxyphenyl)ethylenediamine moiety was synthesized. Therefore, 1 equiv of meso 1,2-bis(2-hydroxyphenyl)ethylenediamine and two equiv of 4-methoxybenzaldehyde were stirred in acetonitrile anhydrous (MeCN). This so formed diimine underwent an acid hydrolysis with a mixture of MeCN/HCl (ratio 4:1) to the meso 1,2-bis(4-methoxyphenyl)ethylenediamine. With this modified diamine, the yellow- to orange-colored ligands **L1–L6** were formed by reacting with the methoxylated or hydroxylated salicylic aldehydes. Before chelation, all ligands were analyzed via ^1^H and ^13^C NMR, MS, and FT-IR. CHN confirmed the elemental composition and purity >95%. Supporting Information (Figures S1–S6) shows the detailed characterization of ligands **L1–L6**.

#### 4.2.2 General Procedure for the Synthesis of Complex **C1–C6**

Complexes were synthesized from the ligands **L1–L6** (data in Supporting Information, Figures S1–S6) via chelation with iron(III)chloride. Briefly, a mixture of 1 equiv substituted 1,2-bis(4-methoxyphenyl)ethylenediamine ligands **L1–L6**, and 1 equiv iron(III)chloride anhydrous was dissolved in ethanol anhydrous (7 mL). A dark solution was formed immediately, and the reaction was allowed to complete for 0.5–2 h under reflux conditions. After cooling to room temperature, the mixture was precipitated. The solid was recrystallized and dried in vacuo. All synthesized complexes were characterized, and the purity was verified based on elemental analysis confirming purity >95%. All spectra are given in the Supporting Information (S7–S12).

Chlorido[*N*,*N*′-bis(3-methoxysalicylidene)-1,2-bis(4-methoxyphenyl)ethylenediamine]iron(III) (**C1**) was performed according to the general procedure. In brief, 1 equiv **L1** (135 mg; 0.25 mmol) and 1 equiv iron(III)chloride (40.6 mg; 0.25 mmol) refluxed in ethanol anhydrous to obtain after recrystallization **C1** as a dark solid (79.6 mg; yield 49%, mp 211 °C). FT-IR ν_max_: 2933 s; 2902 s; 2836 s (OCH_3_); 2572 s; 1629 s br; 1511 s; 1432 s; 1344 s; 1272 s br; 1179 s; 1023 s; 987 s; 839 m; 816 m; 739 s; 584 w cm^–1^. HR-MS (DMSO): *m*/*z* calcd for [M – Cl], 594.1447; found, 594.1451. CHN: calcd: C, 59.32; H, 4.98; N, 4.32. Found: C, 59.29; H, 5.24; N, 4.49. μ_eff_ (Evans method, DMSO-*d*_6_) = 5.22 μ_B_, EPR (9.5 GHz, 98 K) *g*_⊥_ = 4.22, *g*_∥_ = 8.08.

Chlorido[*N*,*N*′-bis(4-methoxysalicylidene)-1,2-bis(4-methoxyphenyl)ethylenediamine]iron(III) (**C2**) was performed according to the general procedure. In brief, 1 equiv **L2** (135.0 mg; 0.25 mmol) and 1 equiv iron(III)chloride (40.6 mg; 0.25 mmol) refluxed in ethanol anhydrous to obtain after recrystallization **C2** as a dark solid (42.72 mg; 26% yield, mp 223 °C). FT-IR: ν_max_ : 2936 s; 2836 m br (OCH_3_); 2572 w; 1610 s br (C=N); 1590 s br; 1511 s br; 1251 s; 1225 s; 1181 s; 1118 s; 1026 s; 974 m; 835 m; 740 w; 632 m; 608 m cm^–1^.HR-MS (DMSO): *m*/*z* calcd for [M – Cl], 594.1447; found, 594.1447. CHN: calcd: C, 59.32; H, 4.98; N, 4.32. Found: C, 59.28; H, 4.83; N 4.28. μ_eff_ (Evans method, DMSO-*d*_6_) = 5.57 μ_B_, EPR (9.5 GHz, 98 K) *g*_⊥_ = 4.23, *g*_∥_ = 8.38.

Chlorido[*N*,*N*′-bis(5-methoxysalicylidene)-1,2-bis(4-methoxyphenyl)ethylenediamine]iron(III) (**C3**) was performed according to the general procedure. In brief, 1 equiv **L3** (135 mg; 0.25 mmol) and 1 equiv iron(III)chloride (40.6 mg; 0.25 mmol) refluxed in ethanol anhydrous to obtain after recrystallization **C3** as a dark solid (108.5 mg; 67% yield, mp 204 °C). FT-IR: ν_max_: 2961 s; 2838 s (OCH_3_); 2573 m; 1600 s br; 1538 s br; 1507 s br; 1460 s br; 1360 m br; 1288 s br; 1247 s br; 1179 s; 1030 s; 828 s br; 741 m; 668 m; 592 w cm^–1^. HR-MS (DMSO): *m*/*z* calcd for [M −Cl], 594.1447; found, 594.1453. CHN: calcd: C, 59.32; H, 4.98; N, 4.32. Found: C, 58.99; H, 5.29; N, 4.59. μ_eff_ (Evans method, DMSO-*d*_6_) = 5.28 μ_B_, EPR (9.5 GHz, 98 K) *g*_⊥_ = 4.17, *g*_∥_ = 8.23.

Chlorido[*N*,*N*′-bis(3-hydroxysalicylidene)-1,2-bis(4-methoxyphenyl)ethylenediamine]iron(III) (**C4**) was performed according to the general procedure. In brief, 1 equiv **L4** (135 mg; 0.26 mmol) and 1 equiv iron(III)chloride (43 mg; 0.26 mmol) refluxed in ethanol anhydrous to obtain after recrystallization **C4** as a dark solid (115.1 mg; 72% yield, mp 201 °C). FT-IR: ν_max_: 3400 m br; 2907 s br; 2835 s br (OCH_3_); 2568 s br; 1611 s br; 1509 s; 1443 s; 1244 s br; 1226 s br; 1180 s; 1005 m; 846 m; 735 m; 640 w cm^–1^. HR-MS (DMSO): *m*/*z* calcd for [M – Cl], 566.1134; found, 566.1128. CHN: calcd: C, 56.49; H, 4.74; N, 4.39. Found: C, 56.78; H, 4.91; N, 4.49. μ_eff_ (Evans method, DMSO-*d*_6_) = 5.22 μ_B_, EPR (9.5 GHz, 98 K) *g*_⊥_ = 4.19, *g*_∥_ = 7.93.

Chlorido[*N*,*N*′-bis(4-hydroxysalicylidene)-1,2-bis(4-methoxyphenyl)ethylenediamine]iron(III) (**C5**) was performed according to the general procedure. In brief, 1 equiv **L5** (135 mg; 0.26 mmol) and 1 equiv iron(III)chloride (43 mg; 0.26 mmol) refluxed in ethanol anhydrous to obtain after recrystallization **C5** as a dark solid (78.2 mg; 48% yield, mp 224 °C). FT-IR: ν_max_: 3399 m br; 2922 m; 2852 m; 1611 s br; 1586 s br; 1511 s; 1463 m; 1227 s; 1177 s; 1125 m; 1024 m; 833 m; 633 m; 606 w. HR-MS (DMSO): *m*/*z* calcd for [M – Cl], 566.1134; found, 566.1138. CHN: calcd: C, 58.13; H, 4.55; N, 4.52. Found: C, 57.93; H, 4.58; N, 4.55. μ_eff_ (Evans method, DMSO-*d*_6_) = 5.45 μ_B_, EPR (9.5 GHz, 98 K) *g*_⊥_ = 4.21, *g*_∥_ = 8.31.

Chlorido[*N*,*N*′-bis(5-hydroxysalicylidene)-1,2-bis(4-methoxyphenyl)ethylenediamine]iron(III) (**C6**) was performed according to the general procedure. In brief, 1 equiv **L6** (135 mg; 0.26 mmol) and 1 equiv iron(III)chloride (43 mg; 0.26 mmol) refluxed in ethanol anhydrous to obtain after recrystallization **C6** as a dark solid (34.3 mg; 21% yield, mp 209 °C). FT-IR: ν_max_: 3436 s br; 3399 m; 2838 s br; 2567 w; 1599 s; 1465 m; 1244 s; 1179 m; 1030 m; 828 m; 670 w cm^–1^. HR-MS (DMSO): *m*/*z* calcd for [M – Cl], 566.1134; found, 566.1127. CHN: calcd: C, 56.49; H, 4.74; N, 4.39. Found: C, 56.50; H, 4.98; N, 4.22. μ_eff_ (Evans method, DMSO-*d*_6_) = 5.17 μ_B_, EPR (9.5 GHz, 98 K) *g*_⊥_ = 4.20, *g*_∥_ = 7.90.

#### 4.2.3 Evaluation of Fluorescence Properties

Fluorescence measurements were performed with a Tecan Infinite M200 spectrophotometer, Tecan Austria GmbH, Grödig, Austria.

#### 4.2.4 Cyclic Voltammetry

The complexes (1 mM) were dissolved in DMSO and adjusted with an appropriate amount of a 0.5 M TBAHFP solution. About 3 mL of the solution was transferred via a syringe to a micro cell. The solution was flushed with argon by inserting the argon line outlet into the solution. After a few minutes of flushing, the argon line outlet was placed on top of the solution to form an argon layer. Afterward, ferrocene was added, and resulting measurements were used for the calculations of the redox potentials. For every measurement, at least three cycles at a scan rate of 100 mV/s were conducted. Calculation of the anodic and cathodic standard potentials was done via EC-Lab software. The *x*-axis (*E* vs Fc) of the voltammograms was set to 0 V with the calculated anodic and cathodic peak potential of ferrocene.

### 4.3 Biological Assays

#### 4.3.1 Cell Lines and Compounds

The mammary carcinoma cell line MDA-MB 231 and the acute myeloid leukemia cell line HL-60 were purchased from the German Collection of Microorganisms and Cell Cultures (DSMZ, Braunschweig, Germany) and authenticated by typing short tandem repeats. The cell lines were grown in RPMI 1640 without phenol red (BioWhittaker, Lonza, Walkersville, MD, USA), supplemented with a solution of glutamine (2 mM), penicillin (100 U/mL), streptomycin (100 μg/mL; Invitrogen Corporation, Gibco, Paisley, Scotland), and FBS (10%; Biowest, Nuaillé, France) at 37 °C in a 5% CO_2_/95% air atmosphere and fed twice weekly.

Ferrostatin-1 and necrostatin-1 were purchased from Sigma-Aldrich (Vienna, Austria), dissolved in DMSO to reach a stock solution of 10 mM and stored at −20 °C. The compounds **C1–C6** were also dissolved in DMSO to prepare a stock solution of 10 mM and stored at room temperature. Cisplatin, dissolved in DMF (10 mM), was used as a reference. On the day of compound addition, the stock solution was diluted with RPMI 1640 without FBS to reach the test concentrations.

#### 4.3.2 Analysis of Antiproliferative Activity

Logarithmically growing MDA-MB 231 cells were resuspended in culture medium at 1 × 10^5^ cells/mL. Thereof 100 μL was plated in triplicate in flat-bottomed 96-well plates (Falcon, Becton Dickinson, Franklin Lakes, NJ, USA) and incubated at 37 °C in a 5% CO_2_/95% air atmosphere for 24 h. Thereafter, compounds were added to reach the final concentrations in a total volume of 150 μL. Exponentially growing HL-60 cells were seeded into U-bottomed 96-well plates with a density of 5 × 10^4^ cells/100 μL per well. After incubation for 2 h, the compounds were added to achieve the test concentrations also in a total volume of 150 μL. All cell lines were incubated with the compounds in total for 72 h.

For the last 12–16 h of incubation, each well was exposed to [^3^H]-thymidine (2 Ci mmol^–1^, Hartmann Analytic, Braunschweig, Germany). Cells were harvested using a semiautomated device, and [^3^H]-thymidine uptake expressed in counts per minute (cpm) was measured in a scintillation counter (Microbeta Trilux, PerkinElmer, Waltham, USA). The proliferation in the absence of the complexes was set to 100%, and the compound’s activity was calculated as percentage of the cell control without compound.

#### 4.3.3 Analysis of Metabolic Activity

Seeding of the cell lines was performed according to the proliferation assay. After the 3 day culture at 37 °C in a 5% CO_2_/95% air atmosphere, cultures were analyzed for metabolic activity using a modified MTT assay (EZ4U kit; Biomedica, Vienna, Austria), according to the manufacturer’s instructions. The evaluation of the metabolic activity was performed according to the proliferation experiments.

#### 4.3.4 Scratch Assay/Wound Healing Assay

Logarithmically growing MDA-MB 231 cells (1 × 10^6^) were resuspended in 4 mL of culture medium, plated in 12-well plates (Greiner bio-one, Kremsmünster, Austria) and incubated at 37 °C in a 5% CO_2_/95% air atmosphere for 24 h. Thereafter, a scratch was performed in the confluent cell layer with a 200 μL pipet tip. The compounds were added immediately at a concentration of 20 μM, and the cells were further incubated for 72 h at 37 °C in a 5% CO_2_/95% air atmosphere. Pictures were taken with a live-cell imaging microscope (Juli Smart Fluorescent Cell Analyzer, Digital Bio/NanoENTek, Seoul, South Korea) every 24 h to document the closing of the scratch.

### 4.4 Cellular Uptake

#### 4.4.1 Quantification of Iron by GF-AAS

MDA-MB 231 cells (0.5 × 10^6^) were seeded in 25 cm^2^ flasks. After reaching 70–80% of confluence, the cell-culture medium was replaced by 3 mL of RPMI + 10% FBS containing the complexes at a final concentration of 5 μM. The flasks were incubated for 20 min, 1, 4, and 24 h, respectively. Thereafter, the cells were washed twice with 1 mL of phosphate-buffered saline (PBS) and treated with accutase (Sigma) for 5 min. As soon as all cells detached from the bottom of the flask, 1 mL of complete medium was added, and the mixture was transferred to a 1.5 mL Eppendorf tube and centrifuged at 2300*g* for 3 min at 4 °C. The cell pellet was washed twice with 1 mL of PBS and stored at −20 °C until analysis. The cell pellets were resuspended in Milli-Q water directly after thawing and lysed by sonication in a cup booster (Sonopuls, Bandelin) three times for 120 s, with cooling at 4 °C, cycle 8, and 65% power.

The iron content of the cell pellets was determined by GF-AAS (M6 Zeeman GFAA-Spectrometer; Thermo Scientific) at 248.3 nm and Zeeman background correction using a 1100 °C ash temperature and a 2100 °C atomization temperature under an argon atmosphere.

The intracellular uptake is presented as the amount of pg Fe/μg protein referred to the cellular protein mass (μg) determined by a classical Bradford assay.

#### 4.4.2 Live Confocal Microscopy

Cells were seeded with a density of 2 × 10^4^ cells in 200 μL per well for 24 h on eight-well chamber slides (ibiTreat, ibidi, Gräfelfing, Germany). Thereafter, the whole medium was replaced with 200 μL of cell-culture medium containing the compounds in the respective final concentration. After a further 24 h incubation, cells were analyzed by confocal microscopy utilizing an inverted microscope (Zeiss Axio Observer Z1, Zeiss, Oberkochen, Germany) in arrangement with a spinning disk confocal system (UltraVIEW VoX, PerkinElmer, Waltham, MA, USA). All the images were generated using a 40× water immersion objective (Zeiss, Vienna, Austria).

#### 4.4.3 Lipid Peroxidation Staining with BODIPY 581/591 C11

MDA-MB 231 cells (2.5 × 10^5^) were seeded in a 24-well plate (Greiner Bio-One International GmbH, Kremsmünster, Austria) in RPMI + 10% FCS. After incubation at 37 °C overnight under a 5% CO_2_/95% air atmosphere, **C2** was added at concentrations of 10 and 20 μM. After 2, 4, 16, and 24 h, the cells were detached with accutase and centrifuged at 200*g* for 5 min. A 2.5 μM BODIPY 581/591 C_11_ staining solution (Invitrogen by Thermo Fisher Scientific, Eugene, OR, USA) was prepared in PBS (Lonza, Verviers, Belgium). Cells were resuspended in 100 μL of the staining solution and incubated for 30 min at 37 °C in the dark. After centrifugation for 10 min at 200*g* and 4 °C, the pellet was resuspended in 200 μL of PBS and immediately analyzed by flow cytometry on a FACSCanto II (Becton Dickinson, Franklin Lakes, NJ, USA).

### 4.5 Statistical Analysis

The Mann–Whitney U test was used to analyze the differences between proliferation and metabolic activity in the absence and the presence of a variable concentration of the test compounds (NCSS software, Kaysville, UT, USA).

IC_50_ values were calculated with Quest Graph IC50 Calculator from AAT Bioquest, Inc.

## Data Availability

The authors will release the atomic coordinates upon article publication.

## Supporting Information Available

^1^H, ^13^C NMR, and FT-IR spectra of **L1**–**L6**; FT-IR, Evans ^1^H NMR, EPR spectra, and cyclic voltammetry of **C1**–**C6**; HPLC of **C2** and **C6** and crystallographic; and crystallographic data of **C2** and **C4**: PDP IDs 2300982 (**C2**) and 2300983 (**C4**) (PDF)
Molecular formula strings of **C1**–**C6** with IC_50_ values in MDA-MB-231 and HL-60 cell lines (CSV)
Datablock **C2** and **C4** (PDF)

## Abbreviations

DMSO: dimethyl sulfoxide
equiv: equivalent
ESI: electrospray ionization
EtOH: ethanol
EPR: electron paramagnetic resonance
FBS: fetal bovine serum
Fer-1: ferrostatin-1
FT-IR: Fourier transform infrared
GFAAS: graphite furnace atomic absorption spectrometry
HPLC: high-performance liquid chromatography
HR MS: high-resolution mass spectrometry
MeCN: acetonitrile
mp: melting point
MTT: 3-(4,5-dimethylthiazol-2-yl)-2,5-diphenyltetrazolium bromide
Nec-1: necrostatin-1
NMR: nuclear magnetic resonance
PBS: phosphate-buffered saline
ROS: reactive oxygen species
RPMI: Rosewell Park Memorial Institute medium
rt: room temperature
SAR: structure–activity relationship
SE: standard error
