# Supplementary material for: Design, Synthesis, Electrochemical, and Biological Evaluation of Fluorescent Chlorido[N,N′-bis(methoxy/hydroxy)salicylidene-1,2-bis(4-methoxyphenyl)ethylenediamine]iron(III) Complexes as Anticancer Agents
Source: J Med Chem. 2023 Nov 28;66(23):15916–25. doi: 10.1021/acs.jmedchem.3c01359 (PMC10726350; doi:10.1021/acs.jmedchem.3c01359)
Supplement: Supplementary file 1 — jm3c01359_si_001.pdf [file jm3c01359_si_001.pdf]

# Supporting Information

# Design, synthesis, electrochemical, and biological evaluation of fluorescent chlorido[*N,N'* -bis(methoxy/hydroxy)salicylidene-1,2-bis(4-methoxyphenyl)ethylenediamine]iron(III) complexes as anticancer agents

Astrid Dagmar Bernkop-Schnürch[1], Donja Chavooshi[1,2], Hubert Aaron Descher[1], Daniel Leitner[3], Heribert Talasz[4], Martin Hermann[5], Klaus Wurst[3], Stephan Hohloch[3], Ronald Gust[*‡1] Brigitte Kircher[*‡2,6]

[1]Department of Pharmaceutical Chemistry, Institute of Pharmacy, CMBI—Center for Molecular Biosciences Innsbruck, CCB—Center for Chemistry and Biomedicine, University of Innsbruck, Innrain 80-82, 6020 Innsbruck, Austria
[2]Immunobiology and Stem Cell Laboratory, Department of Internal Medicine V (Hematology and Oncology), Medical University of Innsbruck, Anichstraße 35, 6020 Innsbruck, Austria
[3]Department of General, Inorganic and Theoretical Chemistry, University of Innsbruck, Innrain 80-82, Innsbruck 6020 Austria
[4]Biocenter, Institute of Medical Biochemistry, Protein Core Facility, Medical University of Innsbruck, Innrain 80-82, 6020 Innsbruck, Austria
[5]Department of Anesthesiology and Critical Care Medicine, Medical University of Innsbruck, Anichstraße 35, 6020 Innsbruck, Austria
[6]Tyrolean Cancer Research Institute, Innrain 66, 6020 Innsbruck, Austria
‡These authors contributed equally.

*Correspondence: Assoc. Prof. Dr. Brigitte Kircher, Department of Internal Medicine V (Hematology and Oncology), Medical University of Innsbruck, Anichstraße 35, 6020 Innsbruck, Austria, brigitte.kircher@i-med.ac.at; Univ. Prof. Dr. Ronald Gust, Department of Pharmaceutical Chemistry, Institute of Pharmacy, University of Innsbruck, Innrain 80-82, 6020 Innsbruck, Austria, rgust@zedat.fu-berlin.de

# Table of Contents


1. Characterization ..... S3

1.1 Characterization of the ligands **L1** - **L6** ..... S3

1.1.1 Characterization of **L1** ..... S3

1.1.2 Characterization of **L2** ..... S5

1.1.3 Characterization of **L3** ..... S7

1.1.4 Characterization of **L4** ..... S9

1.1.5 Characterization of **L5** ..... S11

1.1.6 Characterization of **L6** ..... S13

1.2 Characterization of the iron(III)complexes **C1 – C6** ..... S15

1.2.1 FT-IR spectra ..... S15

1.2.2 HPLC chromatograms ..... S18

1.2.3 Evans $^{1}$H-NMR ..... S19

1.2.4 EPR spectra ..... S25

1.2.5 Crystallographic data of **C2** and **C4** ..... S26

1.2.6 Cyclic voltammetry ..... S30

2. Biological activity ..... S31

2.1 Proliferation ..... S31

2.2 Metabolic activity ..... S31

2.3 Scratch assay ..... S32

2.4 Live confocal microscopy ..... S33

# 1. Characterization

## 1.1 Characterization of the ligands **L1** - **L6**

### 1.1.1 Characterization of **L1**

**[*N,N′*-bis-3-methoxysalicylidene-1,2-bis(4-methoxyphenyl)ethylenediamine]**
Chemical Formula: $C_{32}H_{32}N_2O_6$
Yield: 92 %, mp. 212°C, yellow powder
**HR-MZ:** (DMSO $d_6$): m/z calculated for $[M+H]^+$ 541.2333 = found: 541.2312
**CHN:** calculated: C 71.09 H 5.97 N 5.18 found: C 70.98 H 5.96 N 5.16

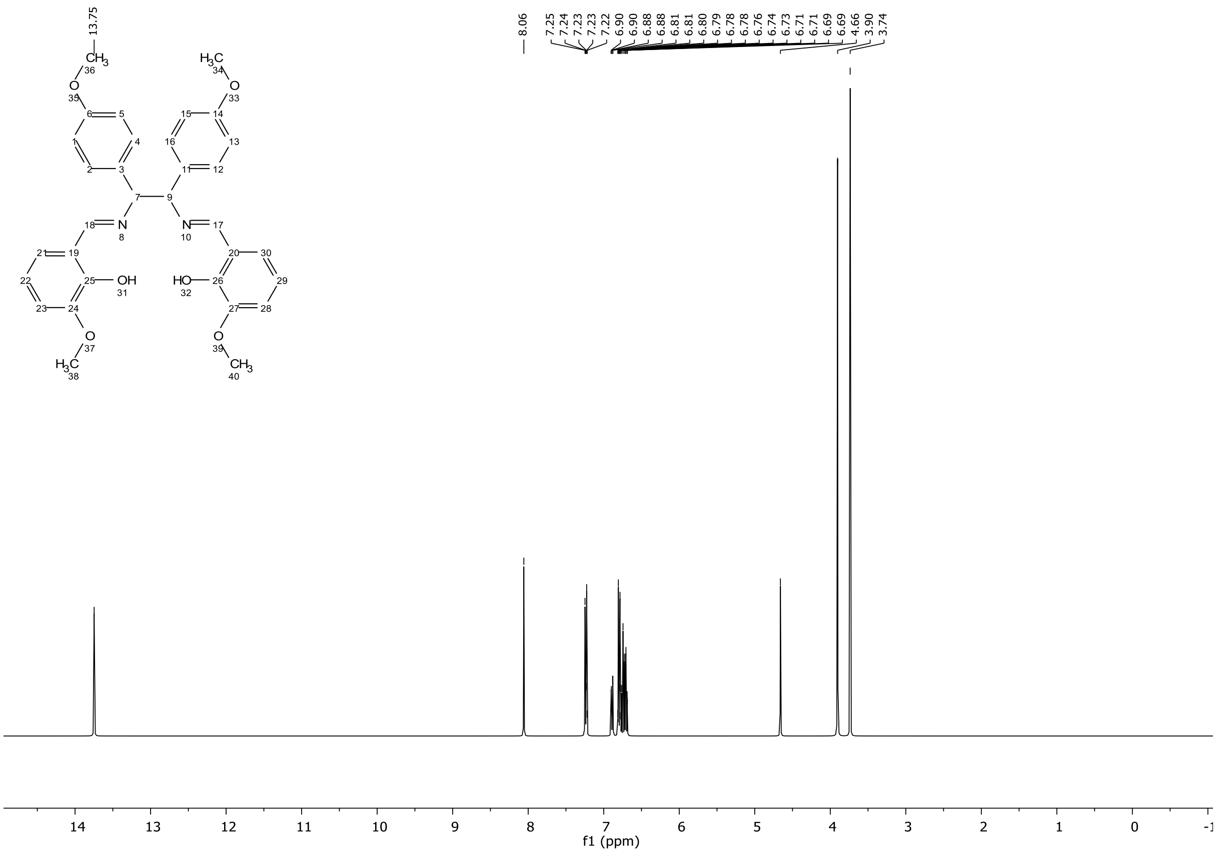


**Figure S1A:**
**$^1$H NMR:** (400 MHz, Chloroform-*d*) δ 13.75 (s, 2H, OH), 8.06 (s, 2H, C(17,18)H), 7.28 – 7.20 (m, 4H, C(22,23,28,29)H), 6.89 (dd, *J* = 7.8, 1.7 Hz, 2H, C(2,4)H), 6.84 – 6.75 (m, 4H, C(1,5,13,15)H), 6.74 (t, *J* = 7.8 Hz, 2H, C(12,16)H), 6.70 (dd, *J* = 7.8, 1.7 Hz, 2H, C(21,30)H), 4.66 (s, 2H, C(7,9)H), 3.90 (s, 6H, OC(34,36)$H_3$), 3.74 (s, 6H, OC(38,40)$H_3$).

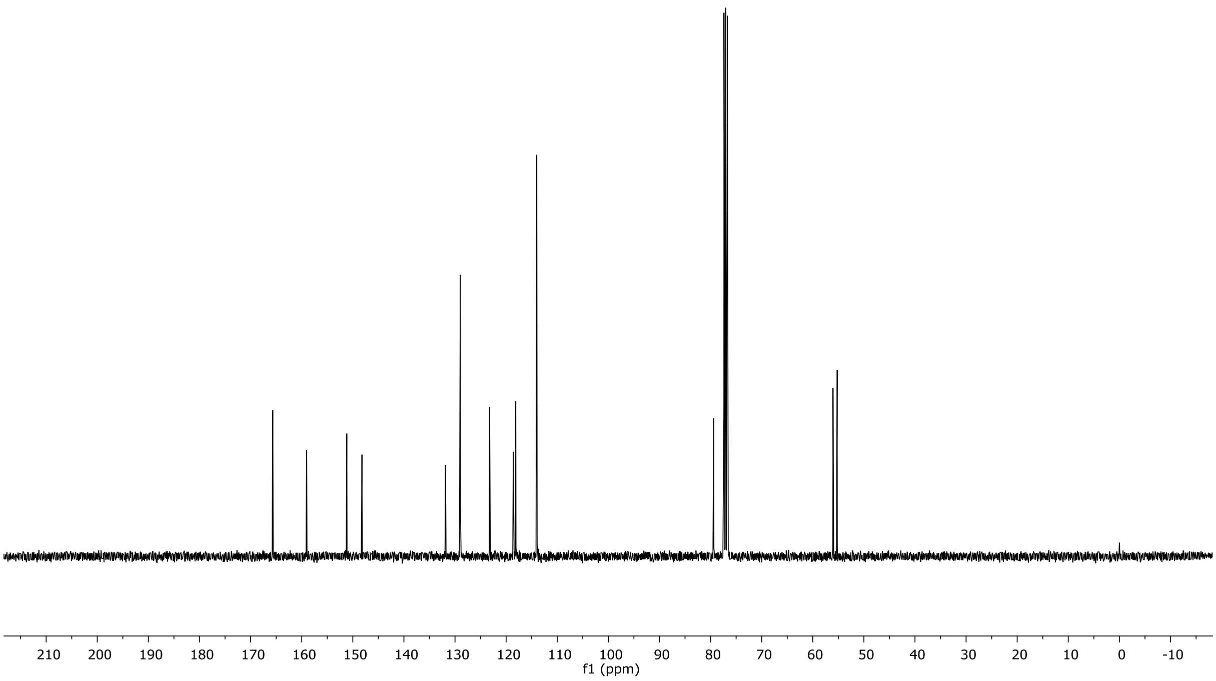


**Figure S1B:**

**$^{13}$C NMR:** (101 MHz, Chloroform-*d*) δ 165.66, 159.03, 151.17, 148.21, 131.86, 128.97, 123.23, 118.60, 118.11, 114.00, 79.39, 56.04, 55.23.

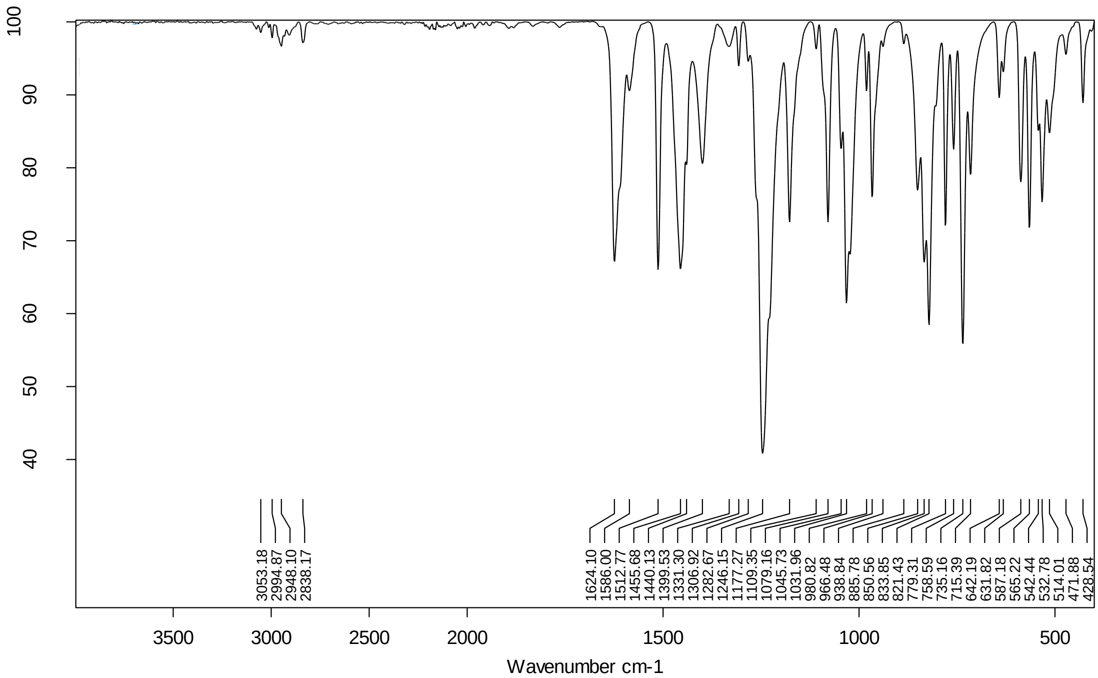


**Figure S1C:**

**FT-IR:** vmax = 3053 w br; 2994 w br; 2948 w; 2838 w; 1624 m; 1586 m; 1512 m; 1455 s; 1440 s; 1399 m; 1331 w; 1306 w; 1282 w; 1246 s; 1177 m; 1109 m; 1079 m; 1045 m: 1031 m; 980 m; 966 m; 936 m; 885 m; 850 m; 833 m; 821 m; 779 m; 758 m; 735 m; 715 m; 642 m; 631 m; 587 m; 566 m; 542 m; 532 m; 514 m; 471 m; 428 m $cm^{-1}$

## 1.1.2 Characterization of **L2**

**[*N,N′*-bis-4-methoxysalicylidene-1,2-bis(4-methoxyphenyl)ethylenediamine]**
Chemical Formula: $C_{32}H_{32}N_2O_6$
Yield: 90 %, mp. 201°C, yellow powder
**HR-MZ:** (DMSO $d_6$): m/z calculated for $[M+H]^+$ 541.2333 = found: 541.2316
**CHN:** calculated C 71.09 H 5.97 N 5.18 found: C 71.02 H 5.94 N 5.13

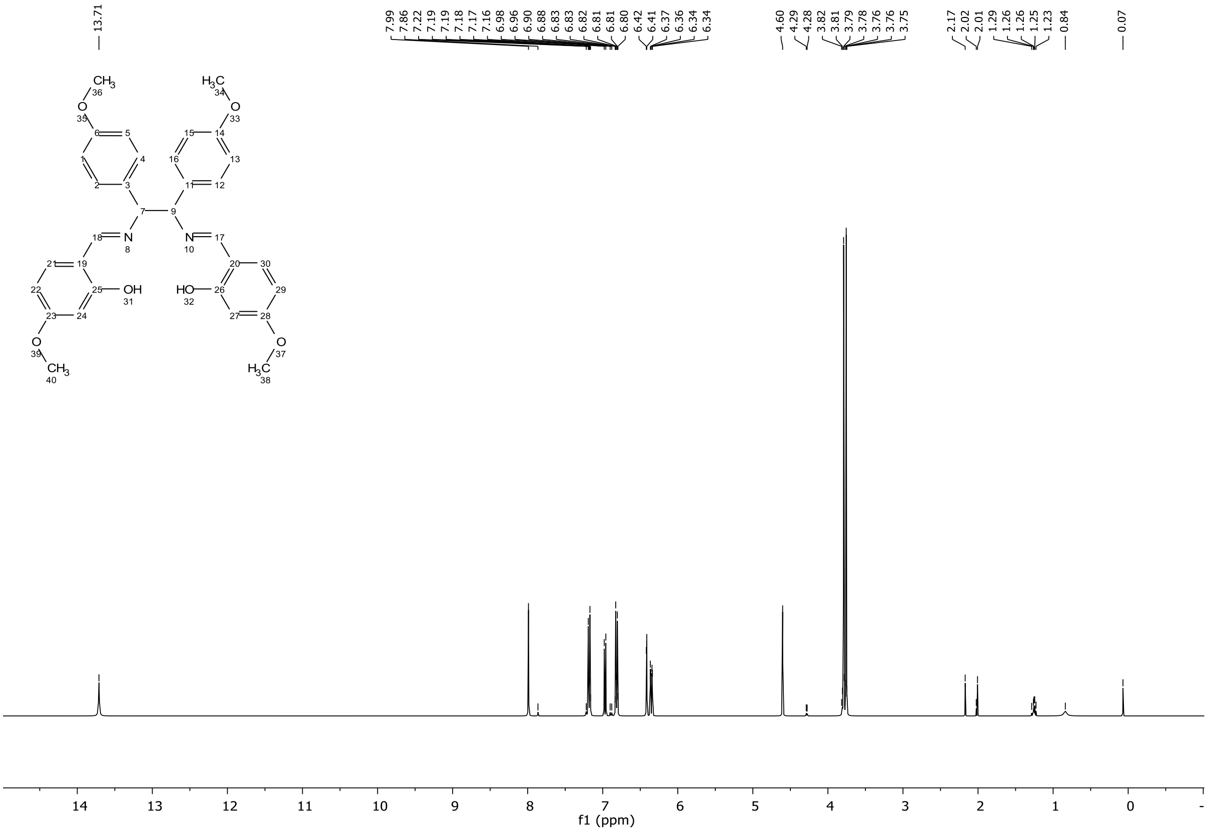


**Figure S2A:**
**$^1$H NMR:** (400 MHz, Chloroform-*d*) δ 13.71 (s, 2H, OH), 7.99 (s, 2H, C(17,18)H), 7.24 – 7.14 (m, 4H, C(2,4,16,12)H), 6.97 (d, *J* = 8.6 Hz, 2H, C(30, 21)H), 6.86 – 6.78 (m, 4H, C(1,5,13,15)H), 6.42 (d, *J* = 2.5 Hz, 2H, C(24,27)H), 6.35 (dd, *J* = 8.5, 2.5 Hz, 2H, C(22,29)H), 4.60 (s, 2H, C(7,9)H), 3.77 (d, *J* = 13.7 Hz, 12H, OC(34,36,38,40)$H_3$).

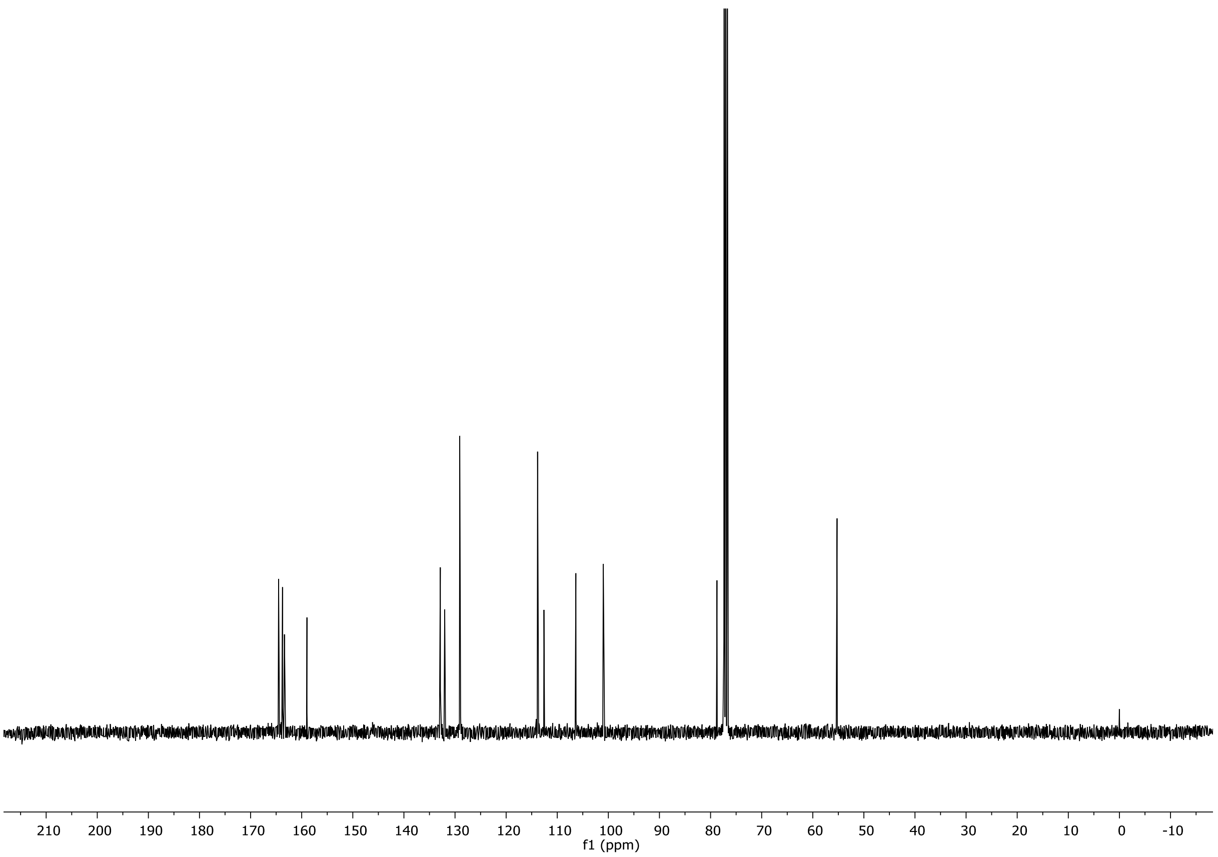

**Figure S2B:**

**$^{13}$C NMR:** (101 MHz, Chloroform-*d*) δ 164.52, 163.76, 163.38, 158.97, 132.87, 132.02, 129.07, 113.85, 112.60, 106.35, 100.99, 78.74, 55.36, 55.24.

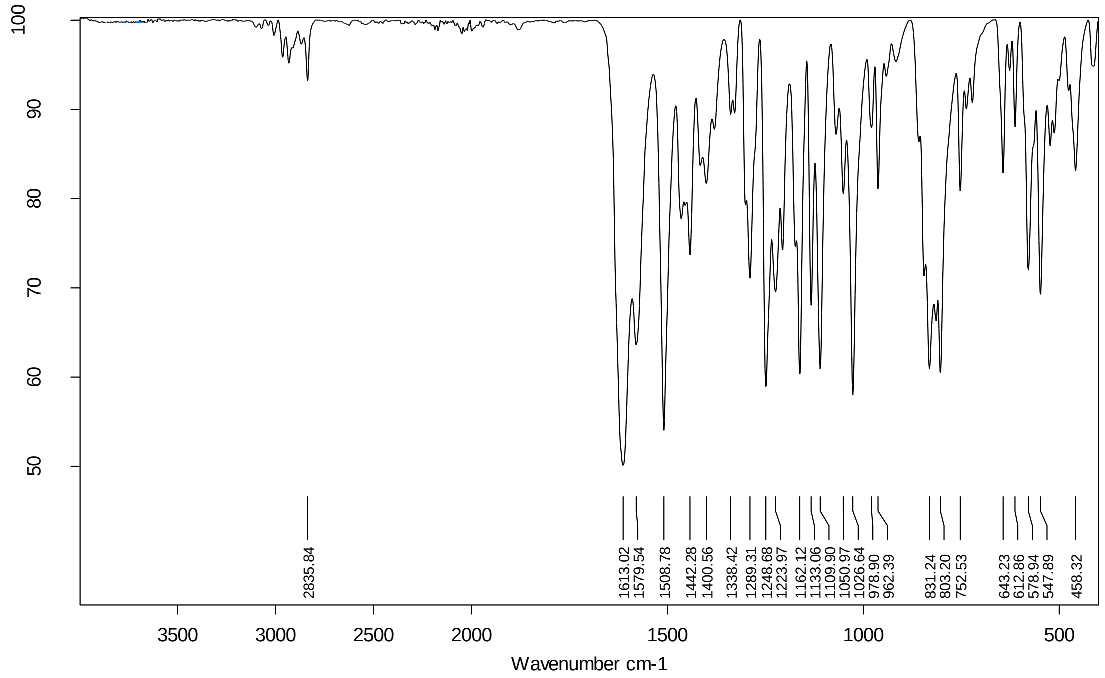

**Figure S2C:**

**FT-IR:** vmax = 2835 w br; 1613 s; 1579 s; 1508 s; 1442 m; 1400 m; 1338 m; 1289 m; 1248 s; 1223 m; 1162 s; 1133 s; 1109 s; 1050 m; 1026 s; 978 m; 962 m; 831 s; 803 s; 752 m; 643 m; 612 m; 578 m; 547 m; 458 m $cm^{-1}$

## 1.1.3 Characterization of **L3**

**[*N,N′*-bis-5-methoxysalicylidene-1,2-bis(4-methoxyphenyl)ethylenediamine]**
Chemical Formula: $C_{32}H_{32}N_2O_6$
Yield: 87 %, mp. 174°C, yellow powder
**HR-MZ:** (DMSO *d*$_6$): m/z calculated for $[M+H]^+$ 541.2333 = found: 541.2315
**CHN:** calculated: C 71.09 H 5.97 N 5.18 found: C 71.43 H 5.88 N 5.41

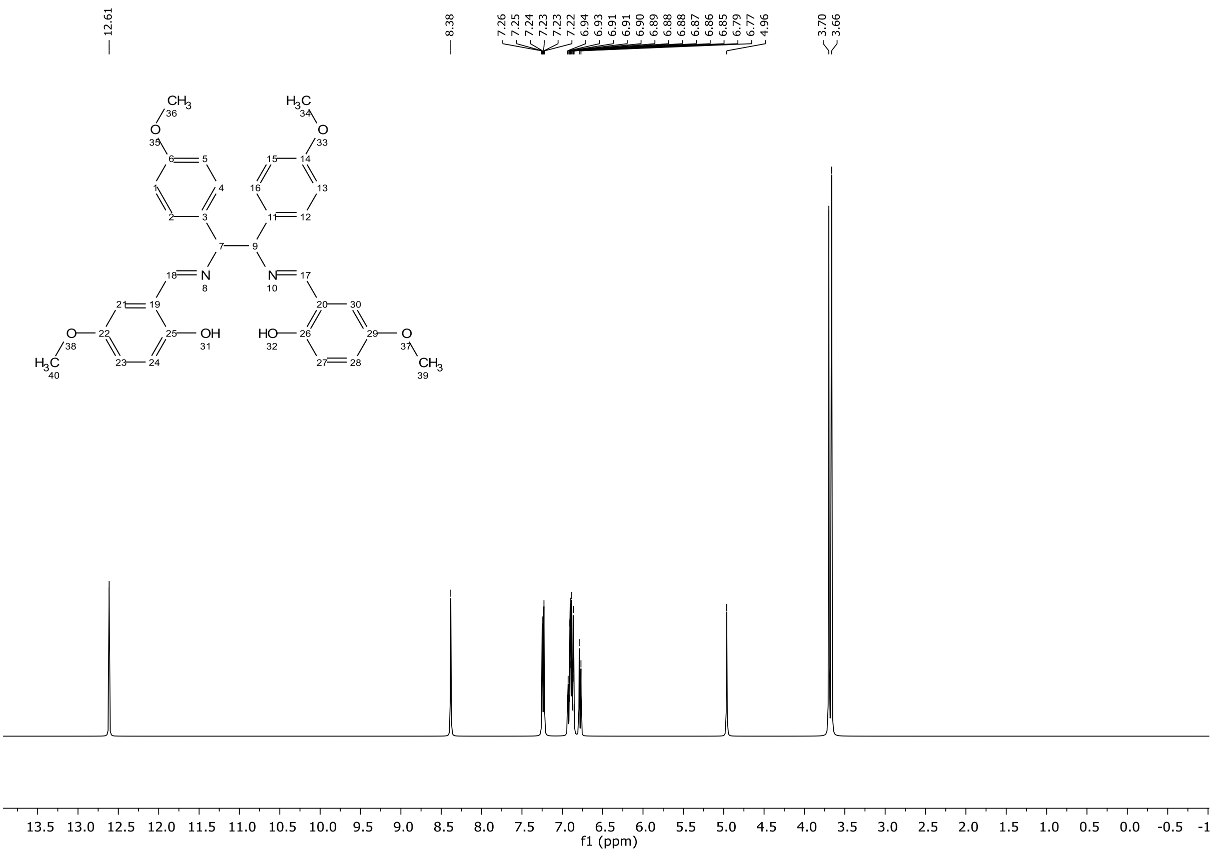


**Figure S3A:**

**$^1$H NMR:** (400 MHz, DMSO-*d*$_6$) δ 12.61 (s, 2H, OH), 8.38 (s, 2H, C(17,18)H), 7.28 – 7.20 (m, 4H, C(2,4,21,30)H), 6.96 – 6.83 (m, 8H, C(1,5,12,13,15,16,23,28)H), 6.78 (d, *J* = 8.6 Hz, 2H, C(24,27)H), 4.96 (s, 2H, C(7,9)H), 3.70 (s, 6H, OC(39,40)H$_3$), 3.66 (s, 6H, OC(34,36)H$_3$).

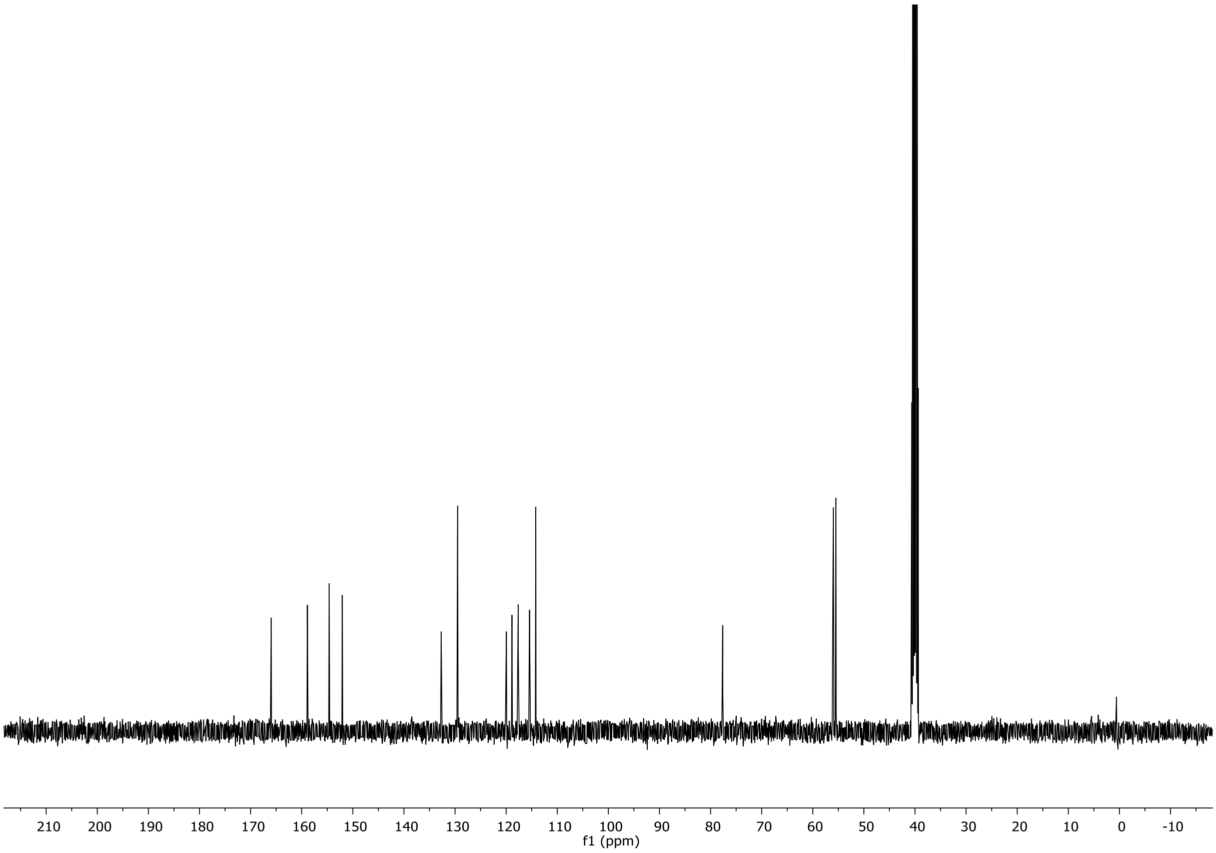


**Figure S3B:**

**$^{13}$C NMR:** (101 MHz, DMSO-$d_6$) δ 165.97, 158.89, 154.63, 152.06, 132.71, 129.49, 119.95, 118.86, 117.64, 115.42, 114.19, 77.63, 55.98, 55.47.

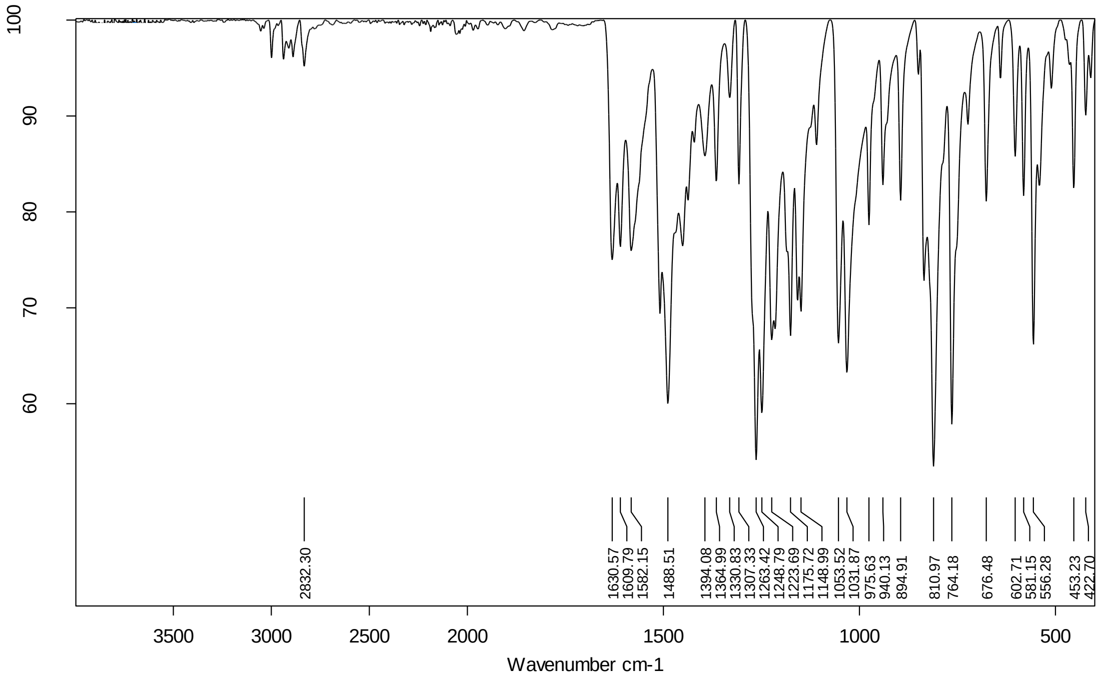


**Figure S3C:**

**FT-IR**: vmax = 2832 w br; 1630 m; 1609 m; 1582 m; 1488 s; 1394 m; 1364 m; 1330 m; 1307m; 1263 s; 1248 s; 1223 s; 1175 m; 1148 m; 1053 s; 1031 s; 975 m; 940 m; 894 m; 810 s; 764 s; 676 m; 602 m; 581 m; 556 s; 453 m; 422 m cm$^{-1}$

## 1.1.4 Characterization of **L4**

**[*N,N′*-bis-3-hydroxysalicylidene-1,2-bis(4-methoxyphenyl)ethylenediamine]**
Chemical Formula: $C_{30}H_{28}N_2O_6$
Yield: 97%, mp. 214°C, yellow powder
**HR-MZ:** (DMSO $d_6$): m/z calculated for $[M+H]^+$ 513.2020 = found: 513.1994
**CHN:** calculated: C 70.30 H 5.51 N 5.47 found: C 70.06 H 5.55 N 5.64

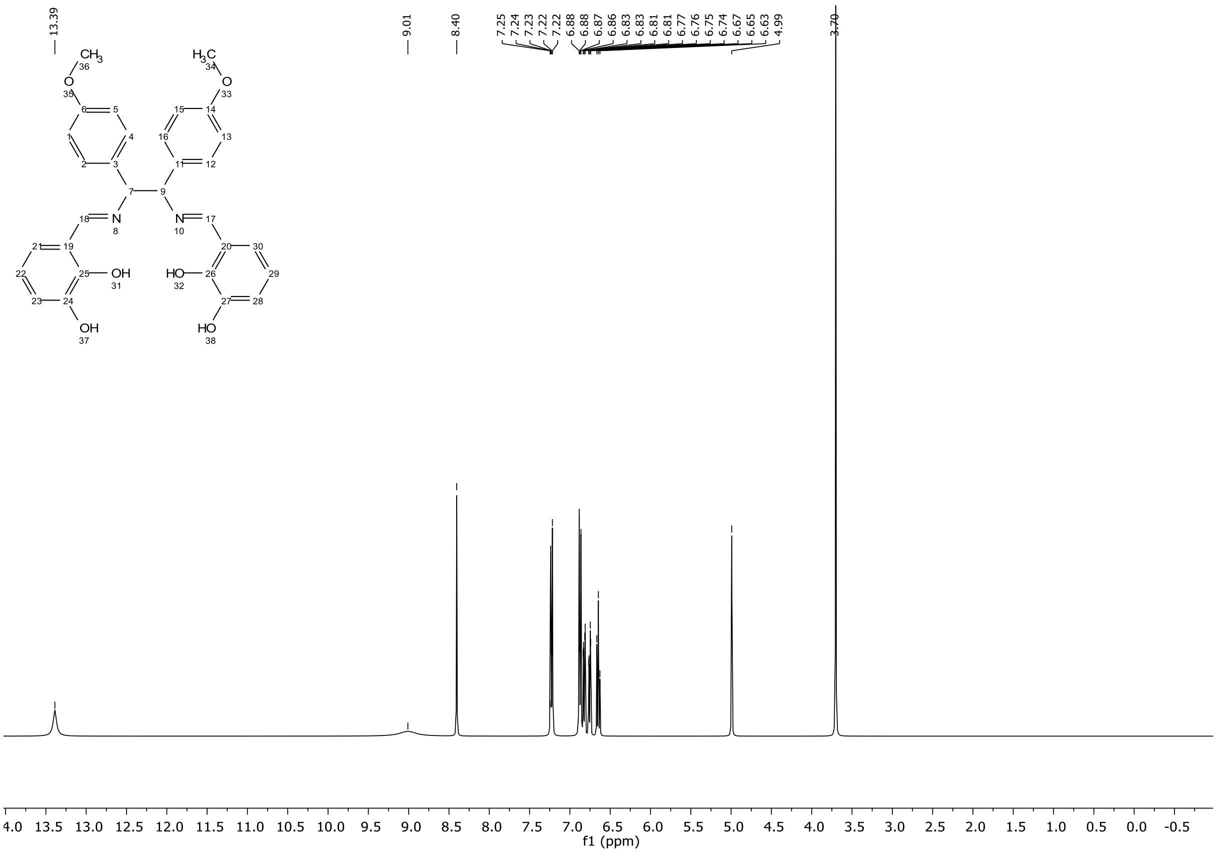


**Figure S4A:**
**$^1$H NMR:** (400 MHz, DMSO-$d_6$) δ 13.39 (s, 2H, O(31,32)H), 9.01 (s, 2H, O(37,38)H), 8.40 (s, 2H, C(17,18)H), 7.27 – 7.19 (m, 4H, C(1,5,13,15)H), 6.91 – 6.84 (m, 4H, C(2,4,23,28)H), 6.82 (dd, $J$ = 7.7, 1.6 Hz, 2H, C(12,16)H), 6.75 (dd, $J$ = 7.9, 1.7 Hz, 2H, C(21,30)H), 6.65 (t, $J$ = 7.7 Hz, 2H, C(22,29)H), 4.99 (s, 2H, C(7,9)H), 3.70 (s, 6H, OC(34,36)$H_3$).

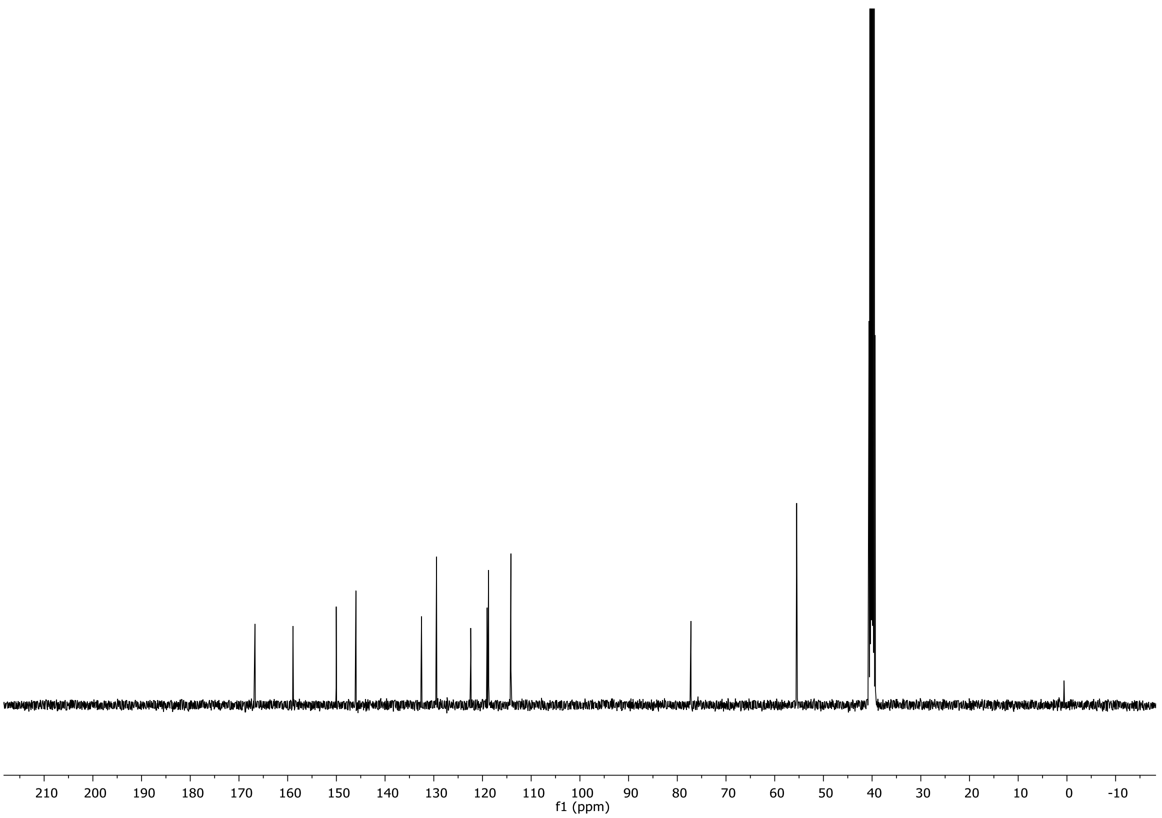


**Figure S4B:**

**$^{13}$C NMR:** (101 MHz, DMSO-$d_6$) δ 166.72, 158.91, 150.03, 145.99, 132.52, 129.44, 122.41, 119.04, 118.75, 114.18, 77.21, 55.49.

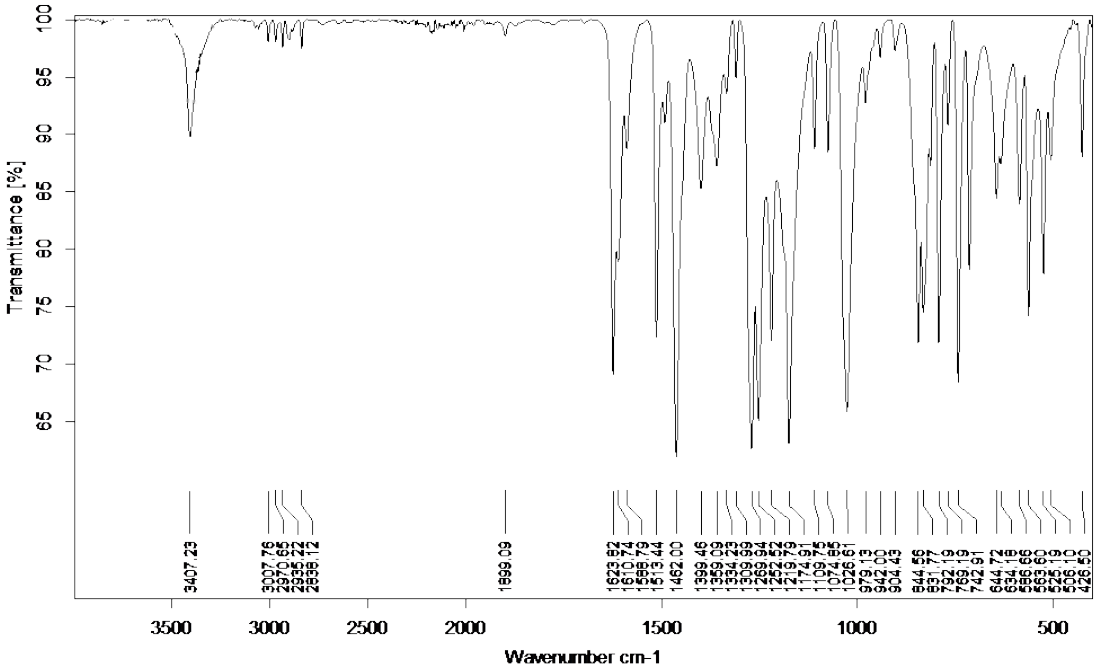


**Figure S4C:**

**FT-IR:** νmax = 3407 m; 3007 w br; 2970 w br; 2935 w br; 2838 w br; 1899 w br; 1623 s; 1610 s; 1588 m; 1513 s; 1462 s; 1399 m; 1359 m; 1334 m; 1309 m; 1269 s; 1252 s; 1219 s; 1174 s; 1109 m; 1074 m; 1026 s; 979 m; 942 m; 904 m; 844 s; 831 s; 792 s; 769 m; 742 s; 644 m; 634 m; 586 m; 563 m; 525 m; 506m; 426 m cm$^{-1}$

## 1.1.5 Characterization of **L5**

**[*N,N'*-bis-4-hydroxysalicylidene-1,2-bis(4-methoxyphenyl)ethylenediamine]**
Chemical Formula: $C_{30}H_{28}N_2O_6$
Yield: 96%, mp. 260°C, light yellow powder
**HR-MZ:** (DMSO $d_6$): m/z calculated for $[M+H]^+$ 513.2020 = found: 513.1996
**CHN:** calculated: C 70.30 H 5.51 N 5.47 found: C 70.27 H 5.44 N 5.43

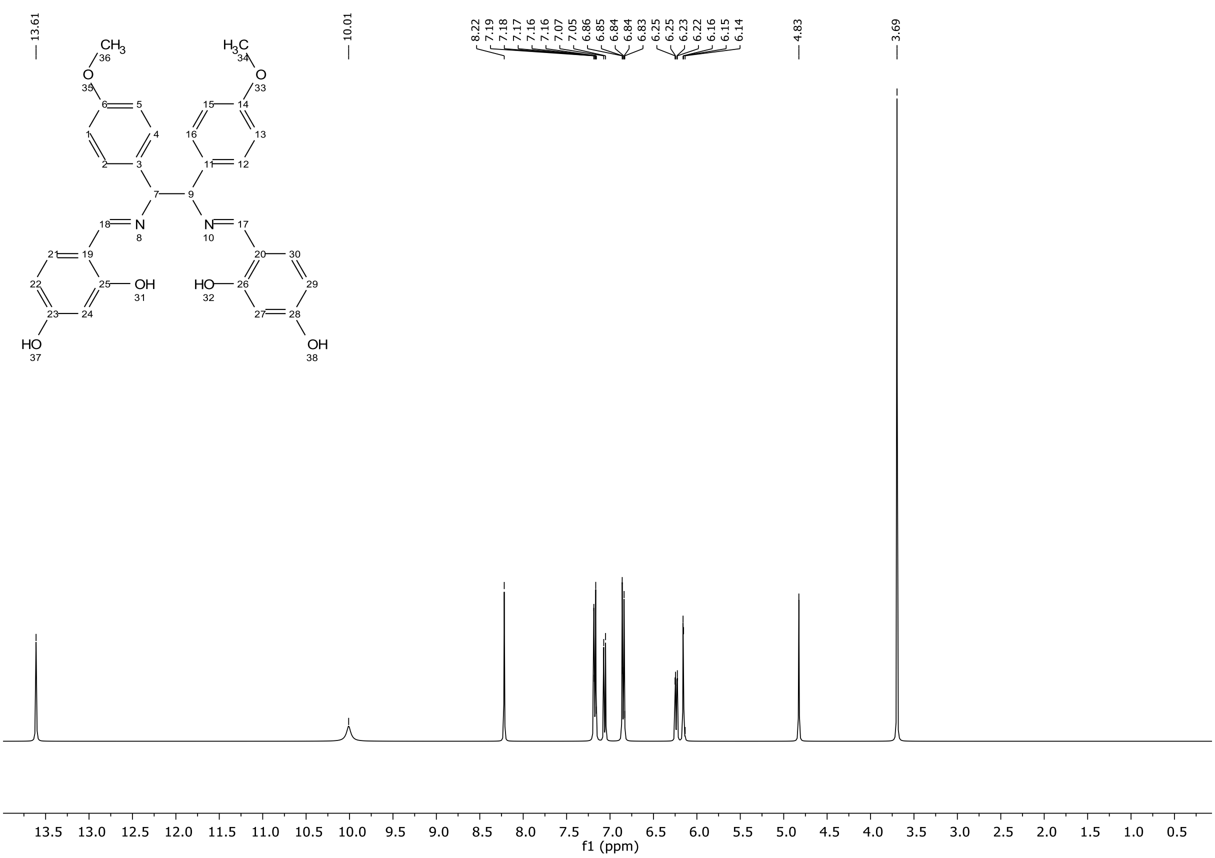


**Figure S5A:**
**$^1$H NMR:** (400 MHz, DMSO-$d_6$) δ 13.61 (s, 2H, O(37,38)H), 10.01 (s, 2H, O(31,32)H), 8.22 (s, 2H, C(17,18)H), 7.21 – 7.13 (m, 4H, C(2,4,12,16)H), 7.06 (d, *J* = 8.5 Hz, 2H, C(30,21)H), 6.89 – 6.81 (m, 4H, C(1,5,13,15)H), 6.24 (dd, *J* = 8.4, 2.3 Hz, 2H, C(22,29)H), 6.16 (d, *J* = 2.2 Hz, 2H, C(24,27)H), 4.83 (s, 2H, C(7,9)H), 3.69 (s, 6H, $OC(34,36)H_3$).

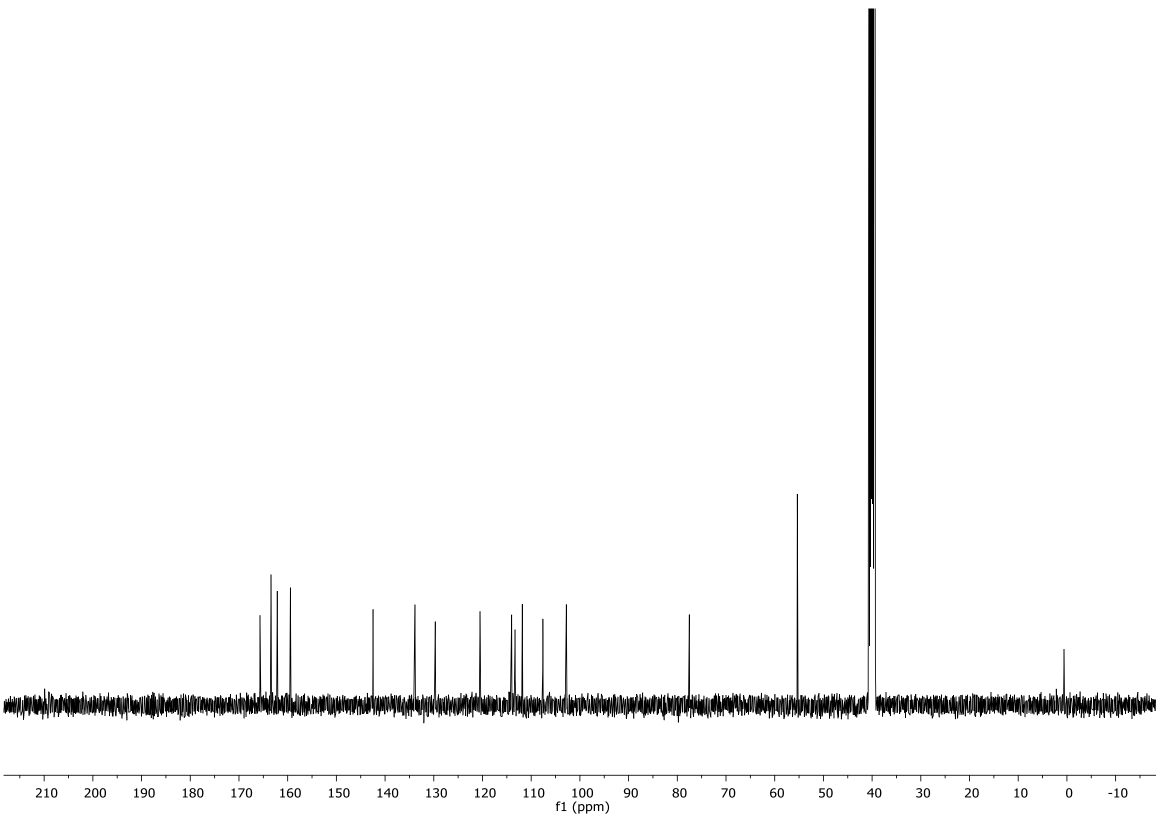

**Figure S5B:**

**$^{13}$C NMR:** (101 MHz, DMSO-$d_6$) δ 165.68, 163.40, 162.13, 159.44, 142.45, 133.88, 129.69, 114.02, 113.30, 111.82, 102.78, 77.53, 55.35.

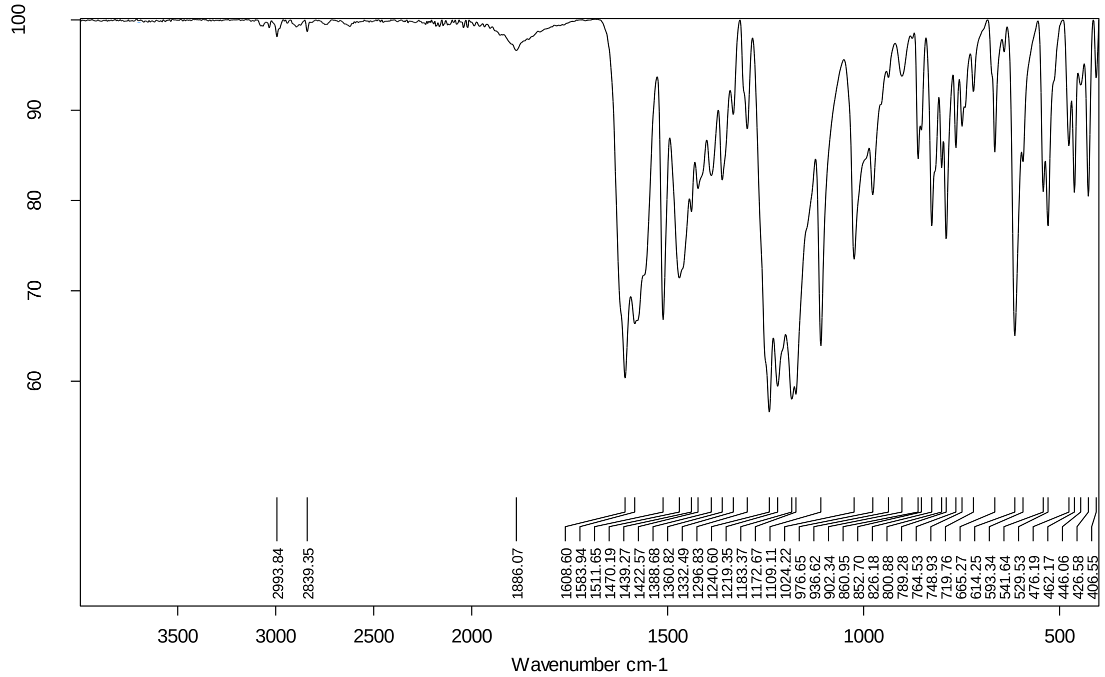

**Figure S5C:**

**FT-IR:** vmax = 2993 w br; 2839 w br; 1886 w; 1608 s; 1583 s; 1511 s; 1470 m; 1439 m; 1422 m; 1388 m; 1360 m; 1332 m; 1296m; 1240 s; 1219 s; 1183 s; 1172 s; 1109 s; 1024m; 976 m; 936 m; 902 m; 860 m; 852 m; 826 m; 800 m; 789 m; 764 m; 748 m; 719 m; 665 m; 614 s; 593 m; 541 m; 529 m; 476 m; 462 m; 446 m; 426 m; 406 m $cm^{-1}$

## 1.1.6 Characterization of **L6**

**[*N,N'*-bis-5-hydroxysalicylidene-1,2-bis(4-methoxyphenyl)ethylenediamine]**
Chemical Formula: $C_{30}H_{28}N_2O_6$
Yield: 93%, mp. 223°C, orange powder
**HR-MZ:** (DMSO $d_6$): m/z calculated for $[M+H]^+$ 513.2020 = found: 513.1998
**CHN:** calculated: C 70.30 H 5.51 N 5.47 found: C 70.29 H 5.87 N 5.43

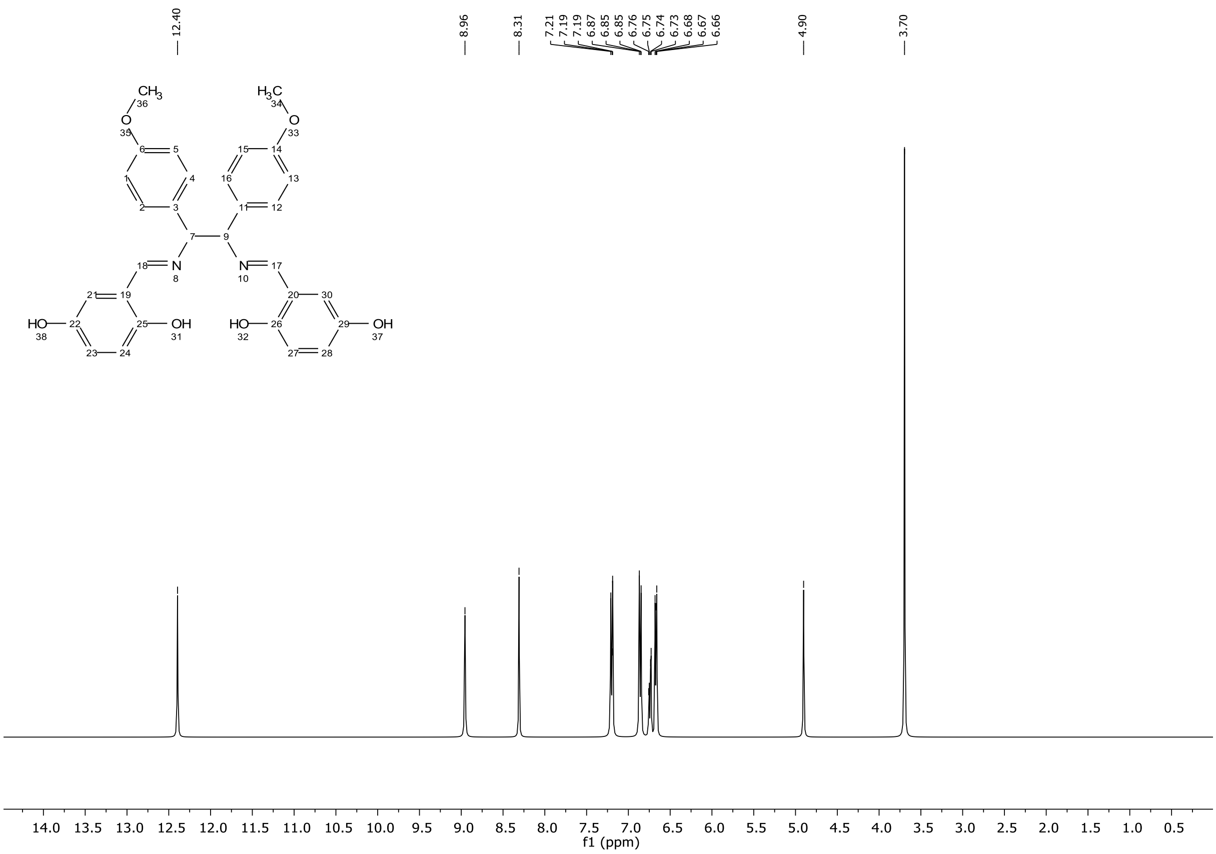


**Figure S6A:**
**$^1$H NMR:** (400 MHz, DMSO-$d_6$) δ 12.40 (s, 2H, O(31,32)H), 8.96 (s, 2H, C(17,18)H), 8.31 (s, 2H, O(37,38)H ), 7.20 (d, $J$ = 8.5 Hz, 4H, C(2,4,21,30)H), 6.86 (d, $J$ = 8.5 Hz, 4H, C(1,5,13,15)H), 6.74 (dd, $J$ = 8.8, 2.9 Hz, 2H, C(12,16)H), 6.70 – 6.64 (m, 4H, C(23,24,27,28)H), 4.90 (s, 2H, C(7,9)H), 3.70 (s, 6H, OC(34,36)$H_3$).

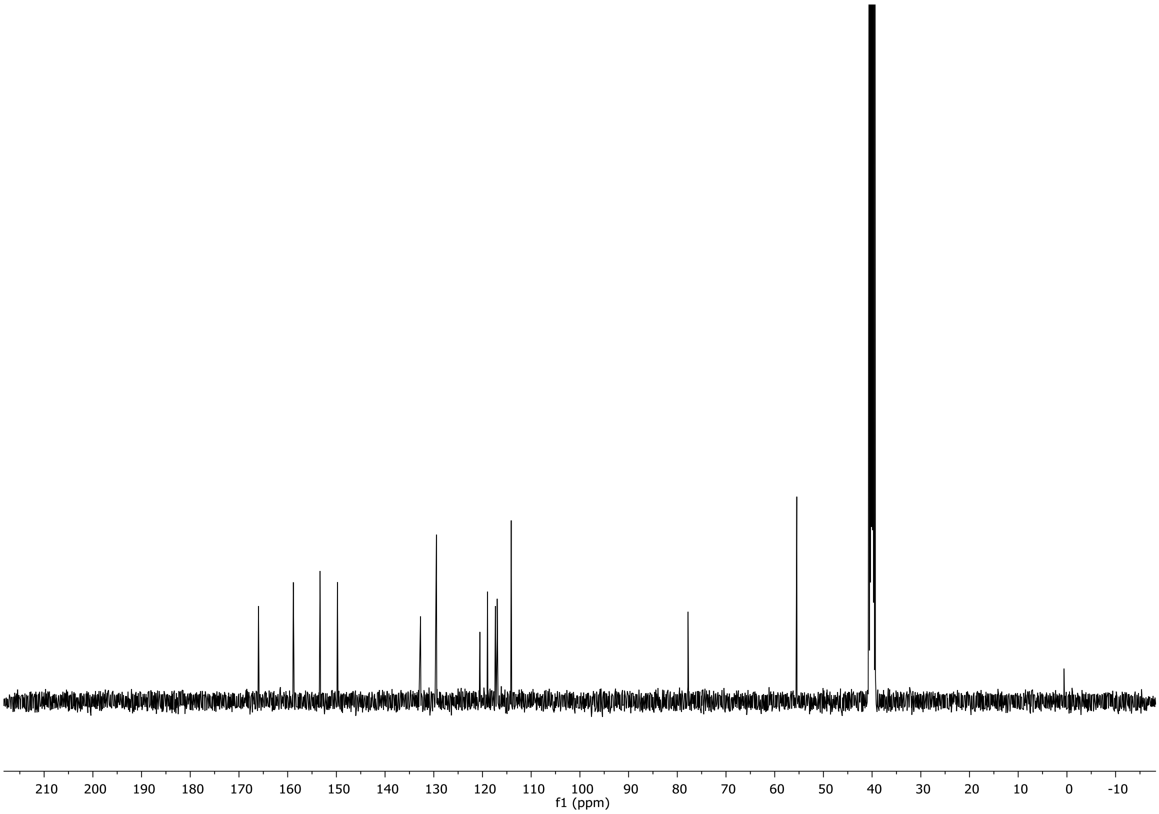


**Figure S6B:**
**$^{13}$C NMR:** (101 MHz, DMSO-$d_6$) δ 165.99, 158.84, 153.34, 149.78, 132.73, 129.46, 120.55, 118.97, 117.35, 116.97, 114.09, 77.79, 55.47.

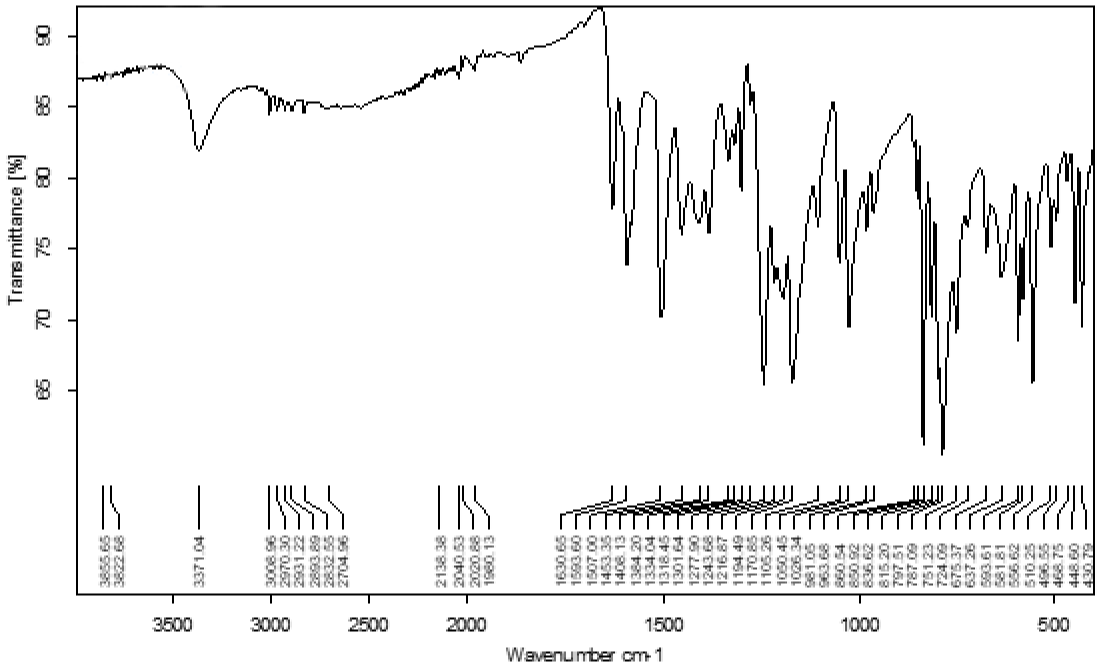


**Figure S6C:**
**FT-IR**: vmax = 3371 w br; 3008 w br; 2970 w br; 2931 w br; 2893 w br; 2832 w br; 2704 w br; 1630 m; 1593 m; 1507 m; 1453 m; 1408 m; 1384 m; 1334 m; 1318 m; 1301 m; 1277 m; 1243 m; 1216 m; 1194 m; 1170 s; 1105 m; 1050 m; 1026 m; 981 m; 963 m; 860 m; 850 m; 836 m; 815 s; 797 m; 787 s; 751 m; 724 m; 675 m; 637 m; 593 m; 581 m; 556 m; 510 m; 496 m; 468 m; 448 m; 430 m cm$^{-1}$

## 1.2 Characterization of the iron(III)complexes **C1** – **C6**

### 1.2.1 FT-IR spectra

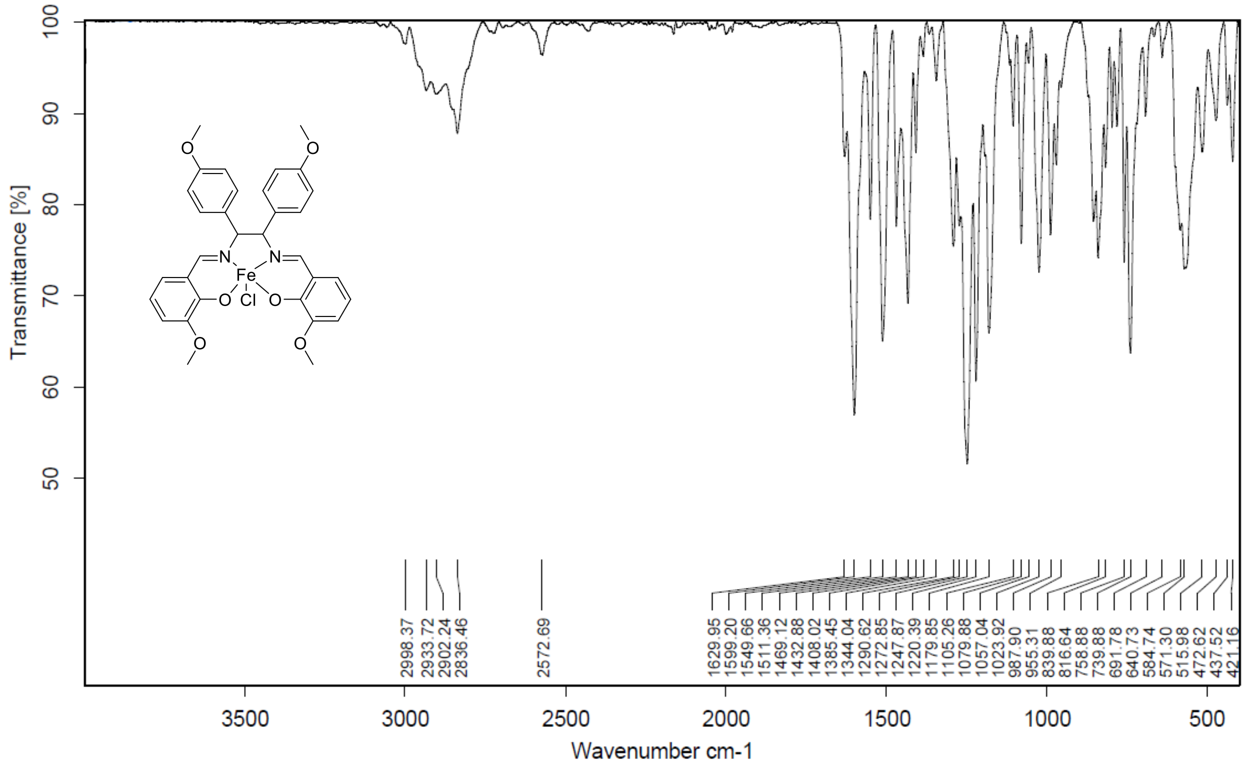


**Figure S7:** FT-IR spectrum of **C1**

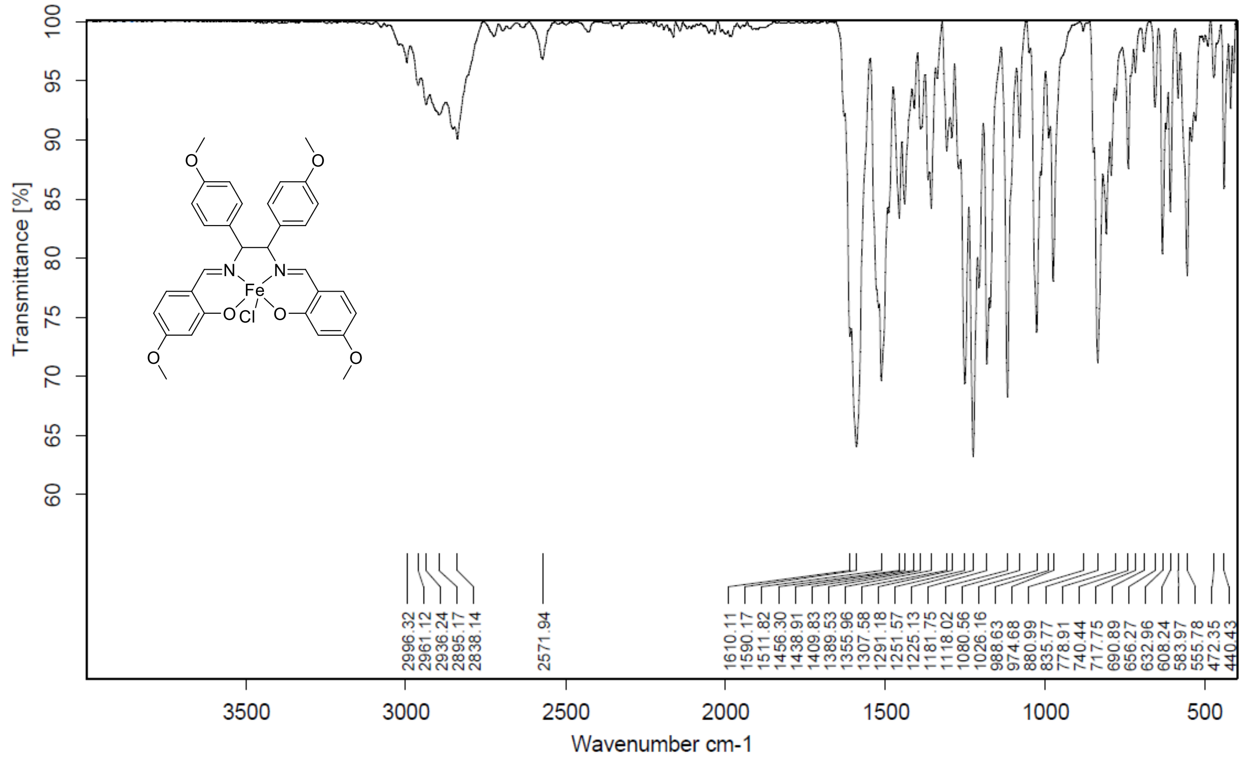


**Figure S8:** FT-IR spectrum **of C2**

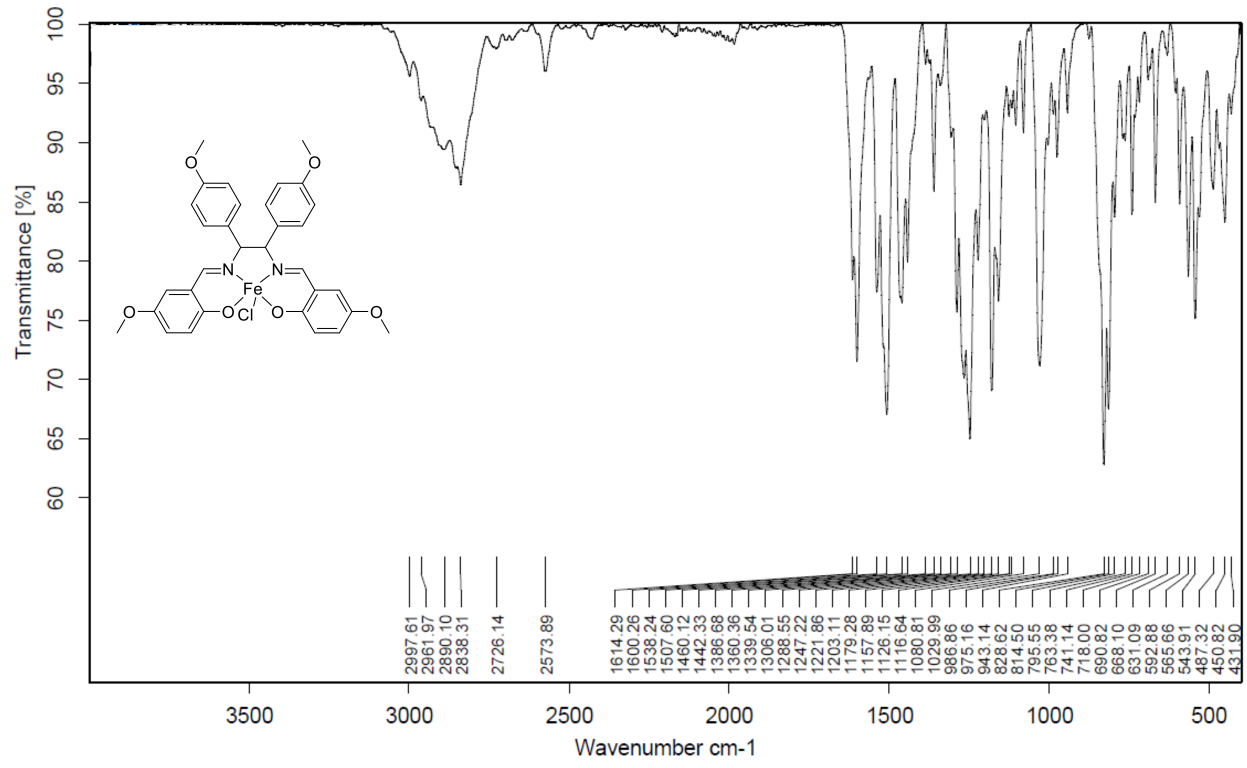


**Figure S9:** FT-IR spectrum **of C3**

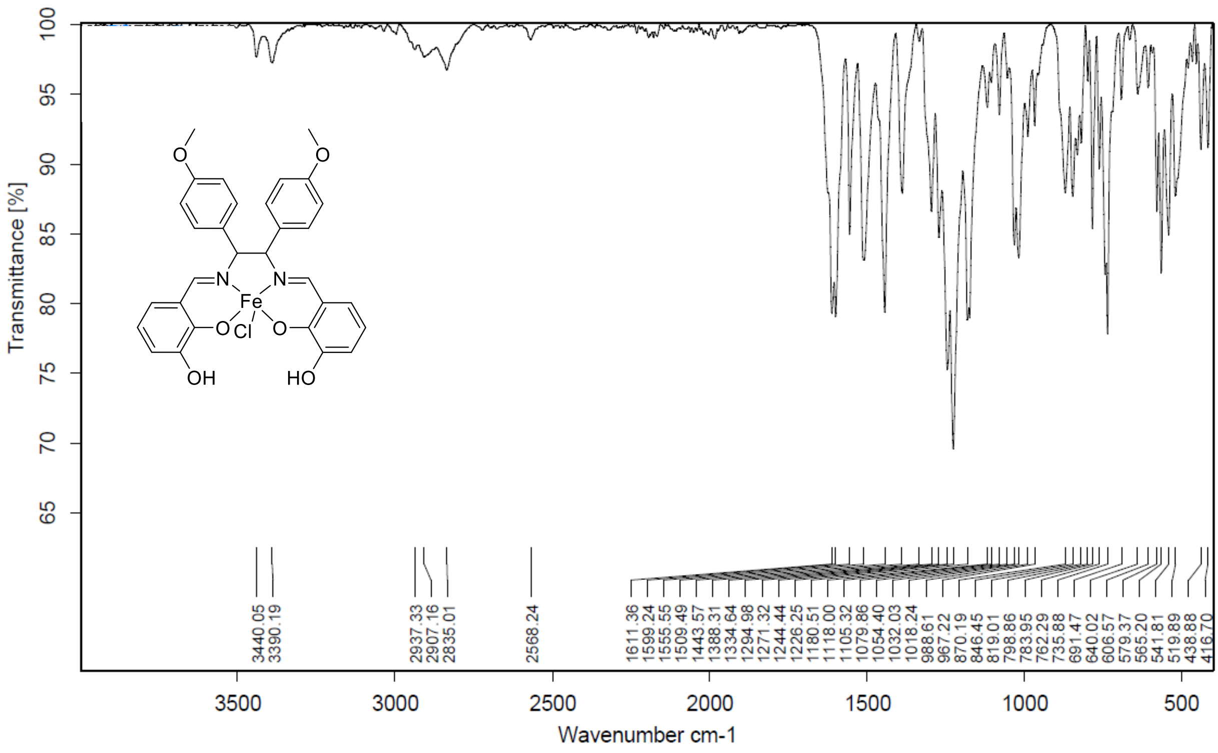


**Figure S10:** FT-IR spectrum **of C4**

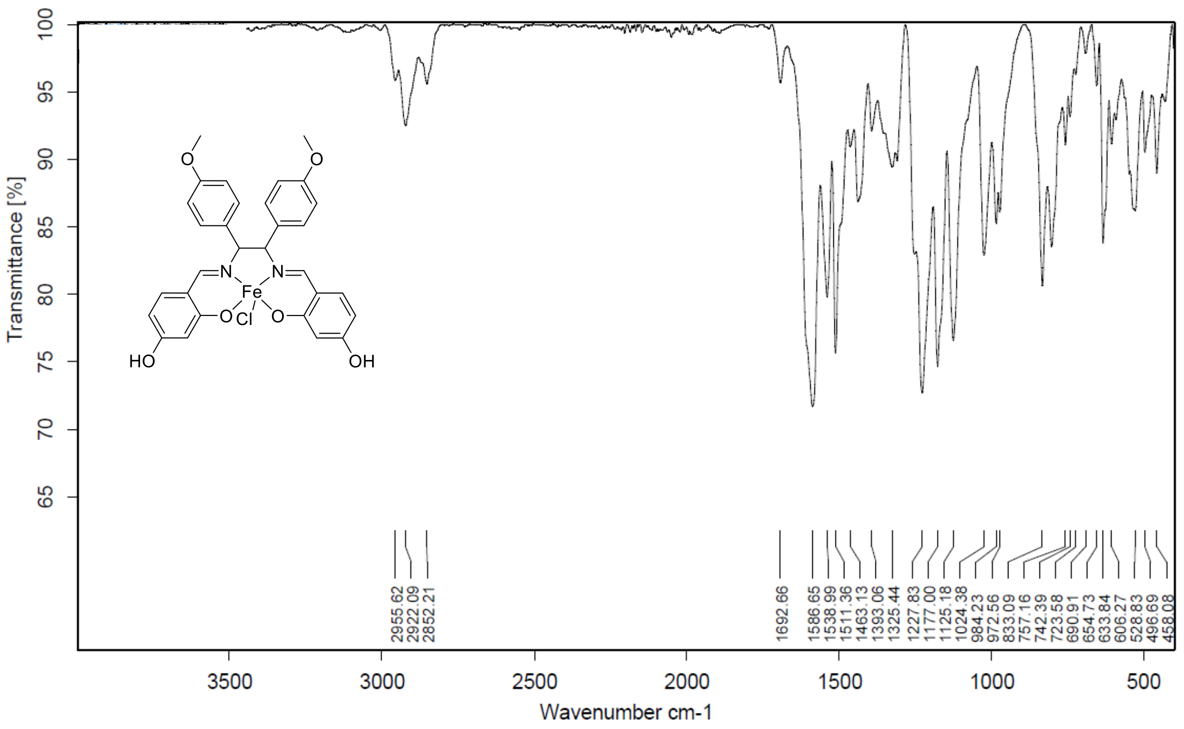


**Figure S11:** FT-IR spectrum **C5**

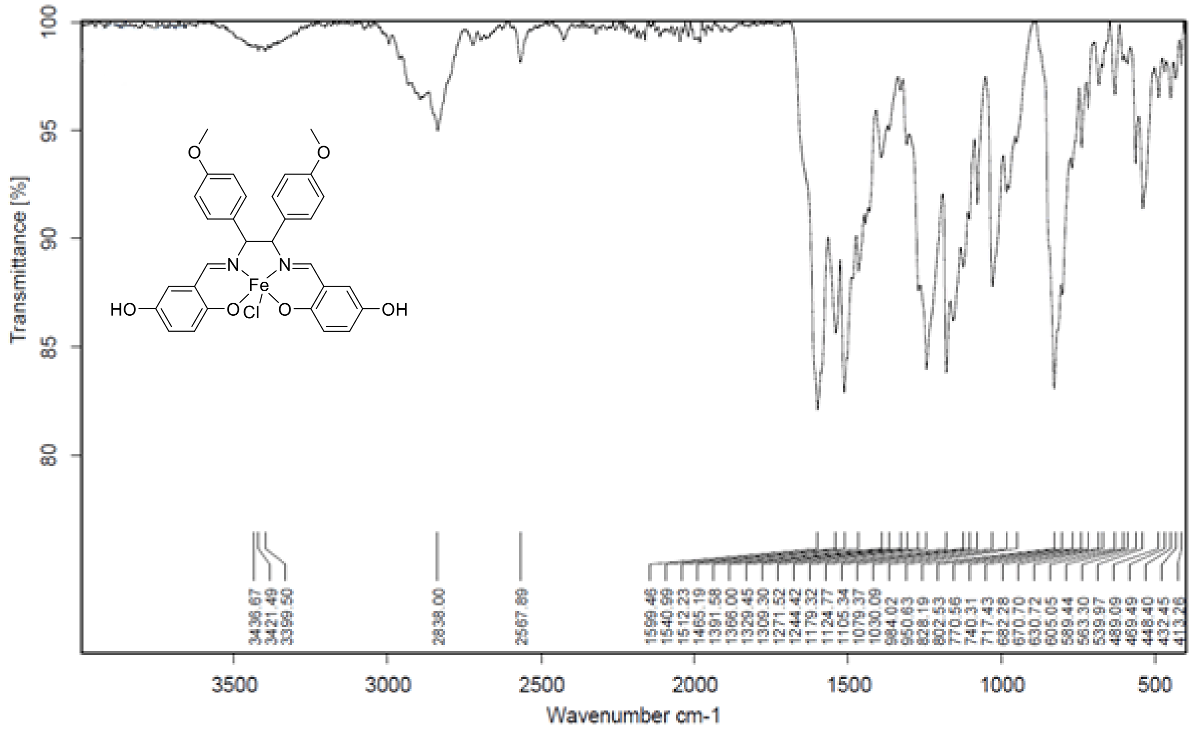


**Figure S12:** FT-IR spectrum of **C6**

## 1.2.2 HPLC chromatograms

Lead complexes **C2** and **C6** (approximately 0.5 mg) were dissolved in 0.75 ml Methanol (HPLC-grade) and 30 µl were injected to a Shimadzu Nexera-i-LC-2040C-3D equipped with a KNAUER column RP-18 end capped 100–4.6 mm column. Gradient elution (85/15 (v/v) to 90/10 (v/v)) of MeOH/water was used and the software LabSolutions assisted for data processing. Purity was calculated using the peak area %.

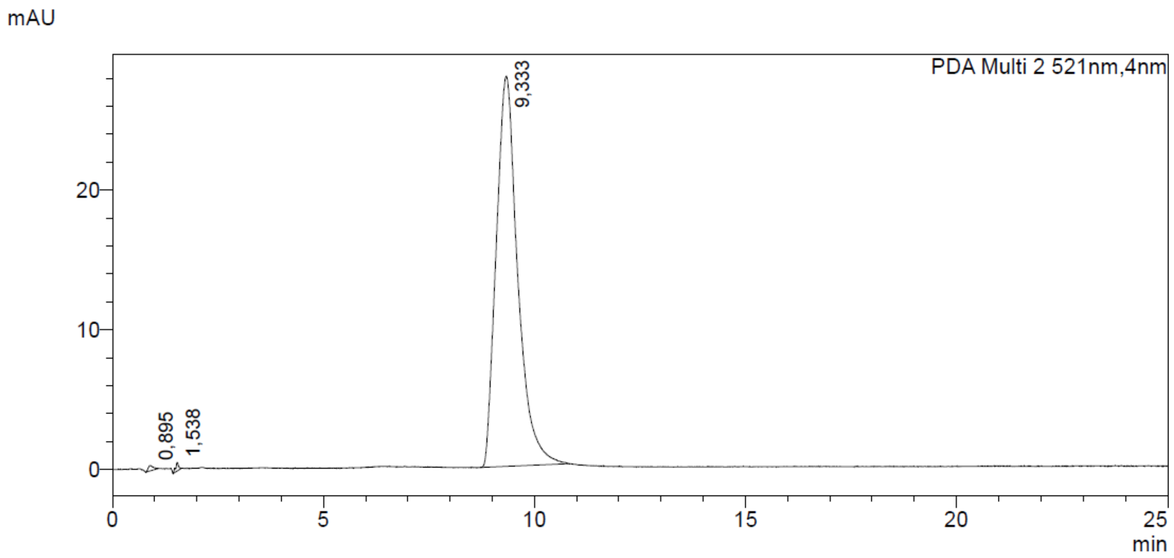


**Figure S13:** HPLC chromatogram of **C2** dissolved in MeOH (purity: 99.4%).

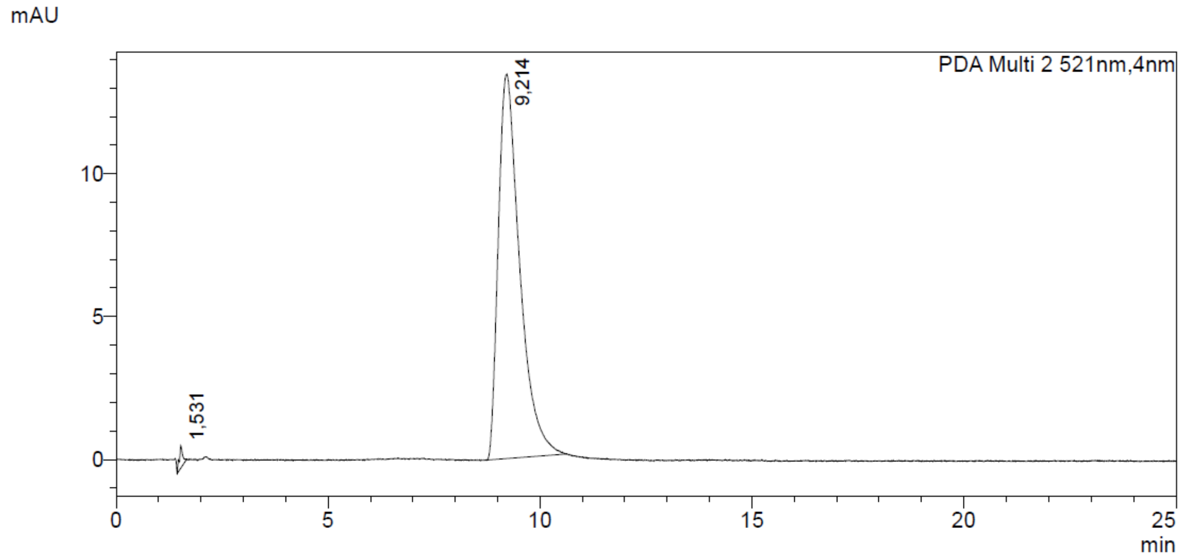


**Figure S14:** HPLC chromatogram of **C6** dissolved in MeOH (purity: 99.1%).

### 1.2.3 Evans $^1$H-NMR

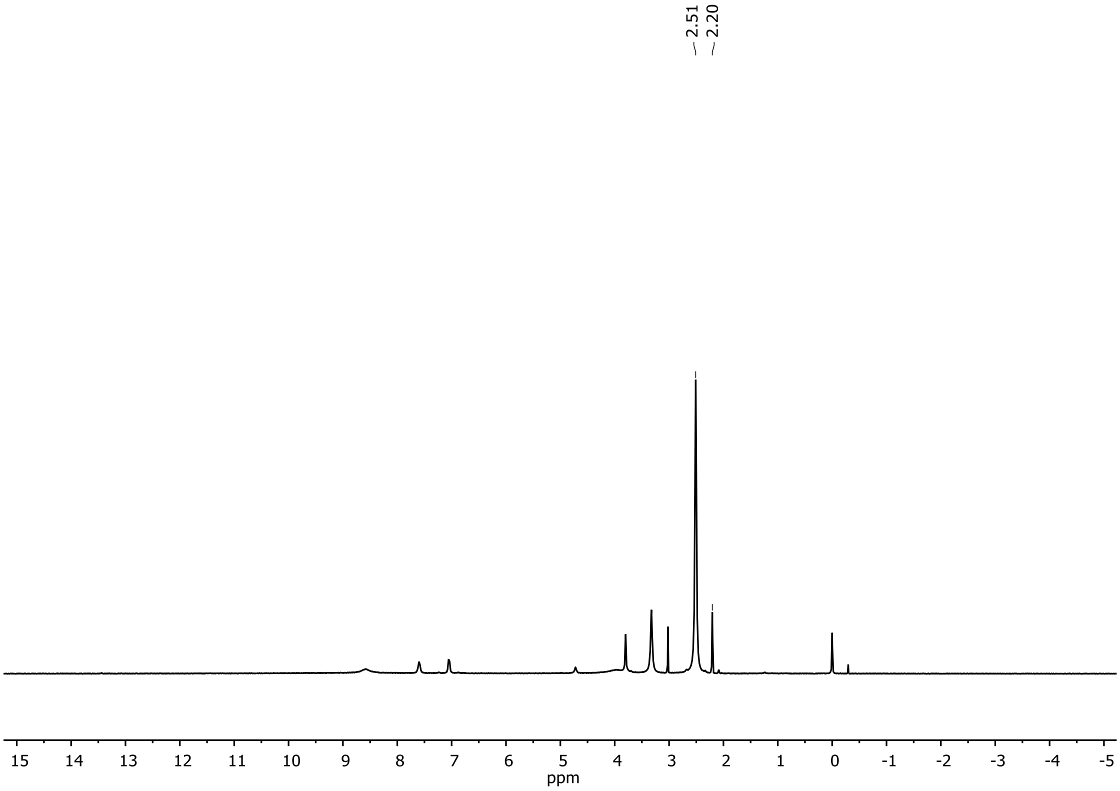


**Figure S15:** Evans $^1$H-NMR (400 MHz) of **C1** in DMSO-$d_6$

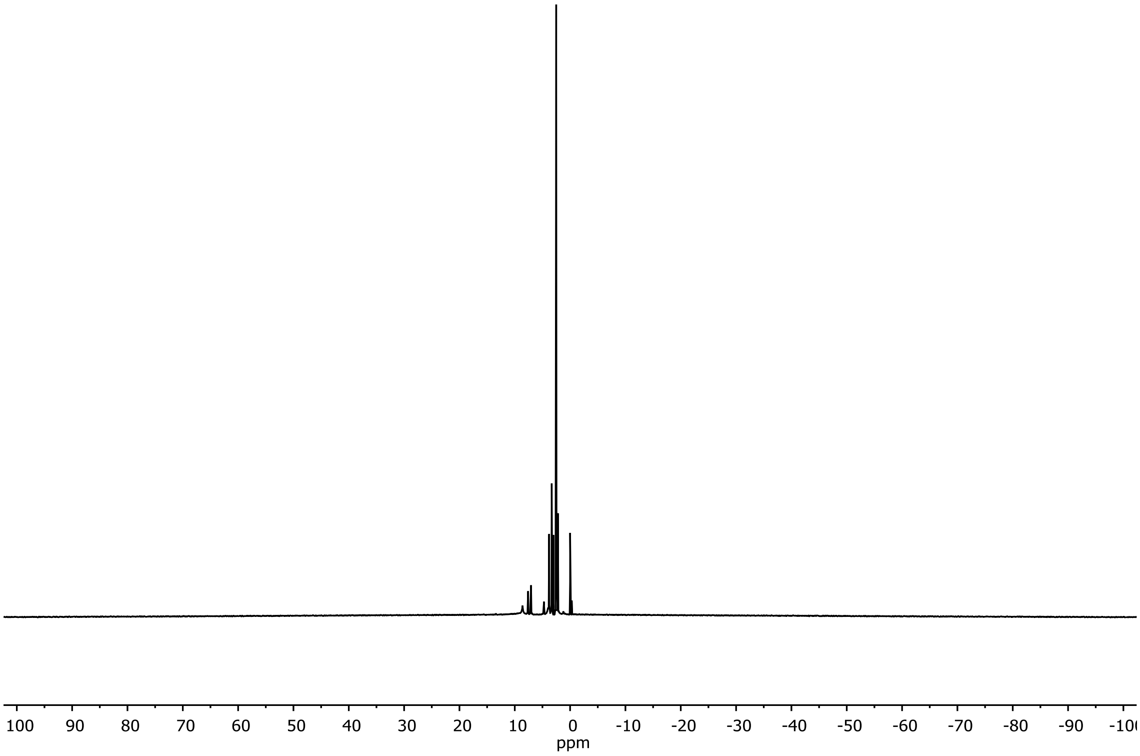


**Figure S16:** Evans $^1$H-NMR (400 MHz) of **C1** in DMSO-$d_6$ between -100 and 100 ppm

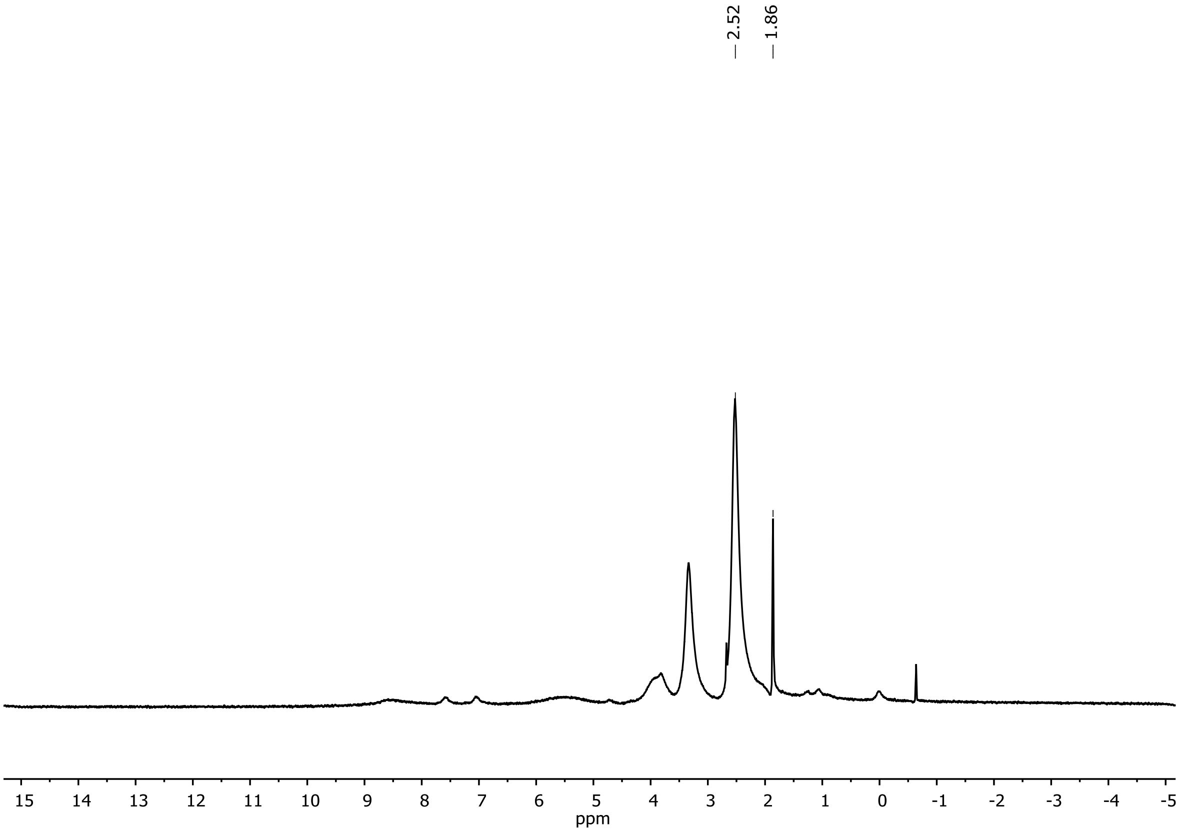


**Figure S17:** Evans $^{1}$H-NMR (400 MHz) of **C2** in DMSO-$d_6$

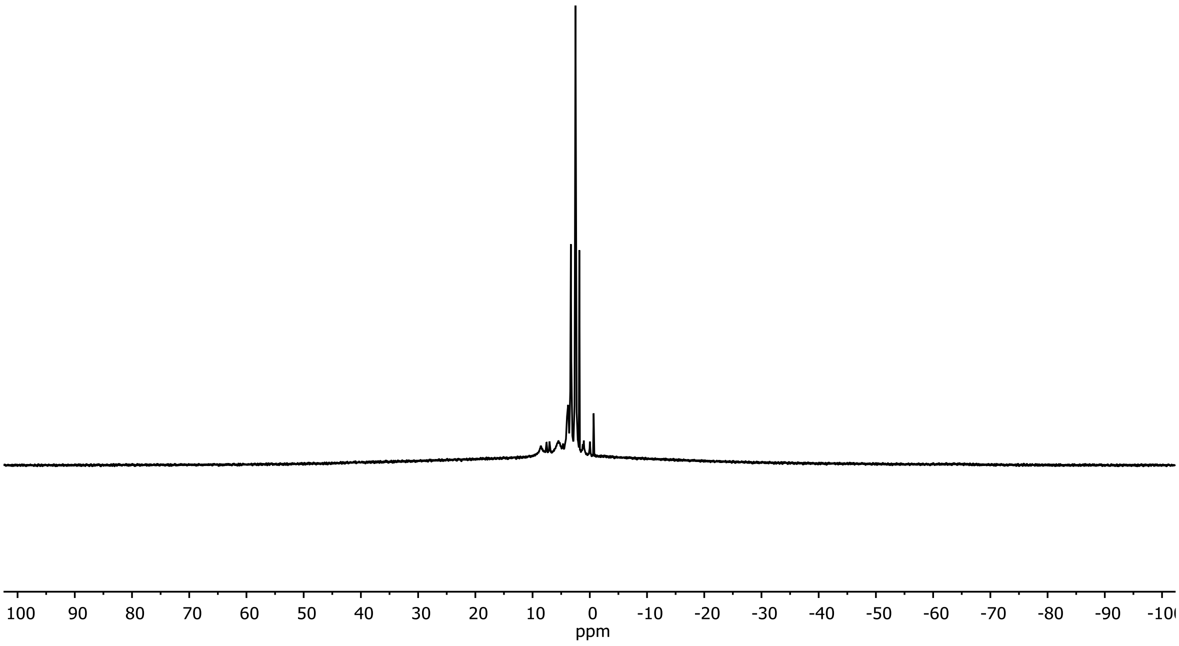


**Figure S18:** Evans $^{1}$H-NMR (400 MHz) of **C2** in DMSO-$d_6$ between -100 and 100 ppm

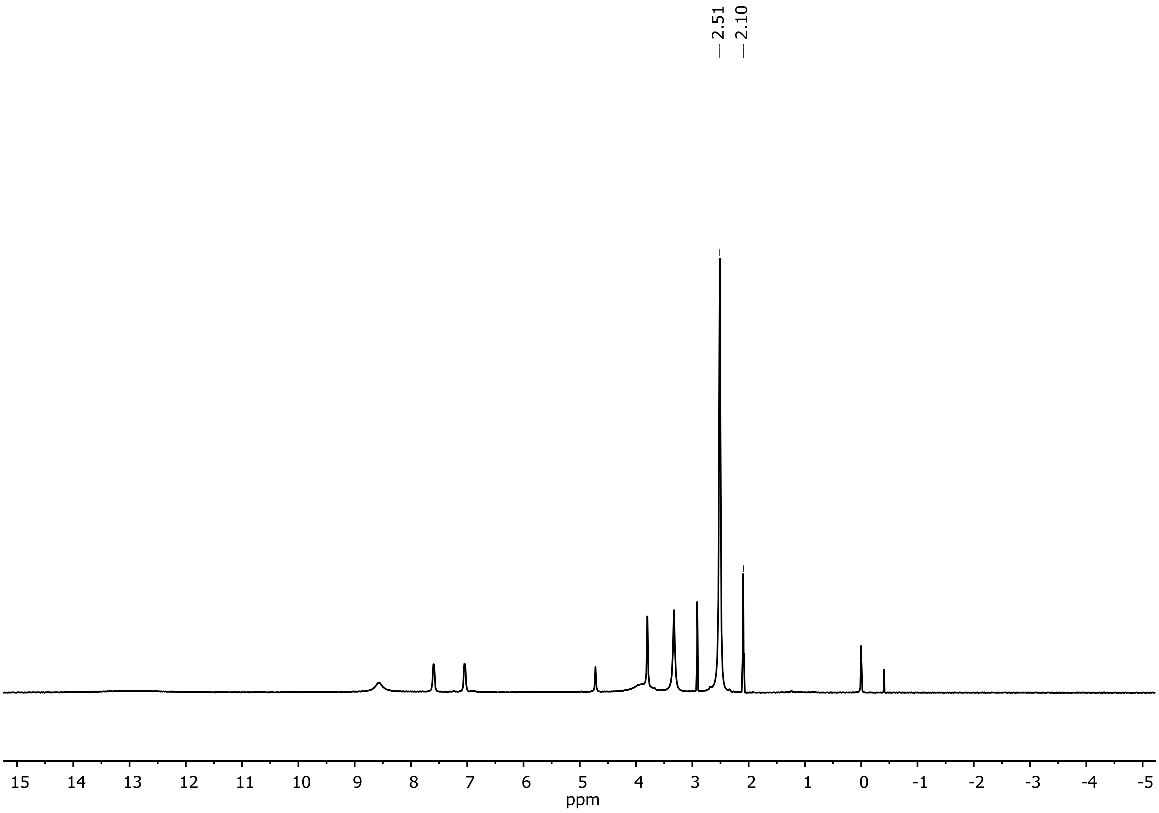


**Figure S19:** Evans $^{1}$H-NMR (400 MHz) of **C3** in DMSO-$d_6$

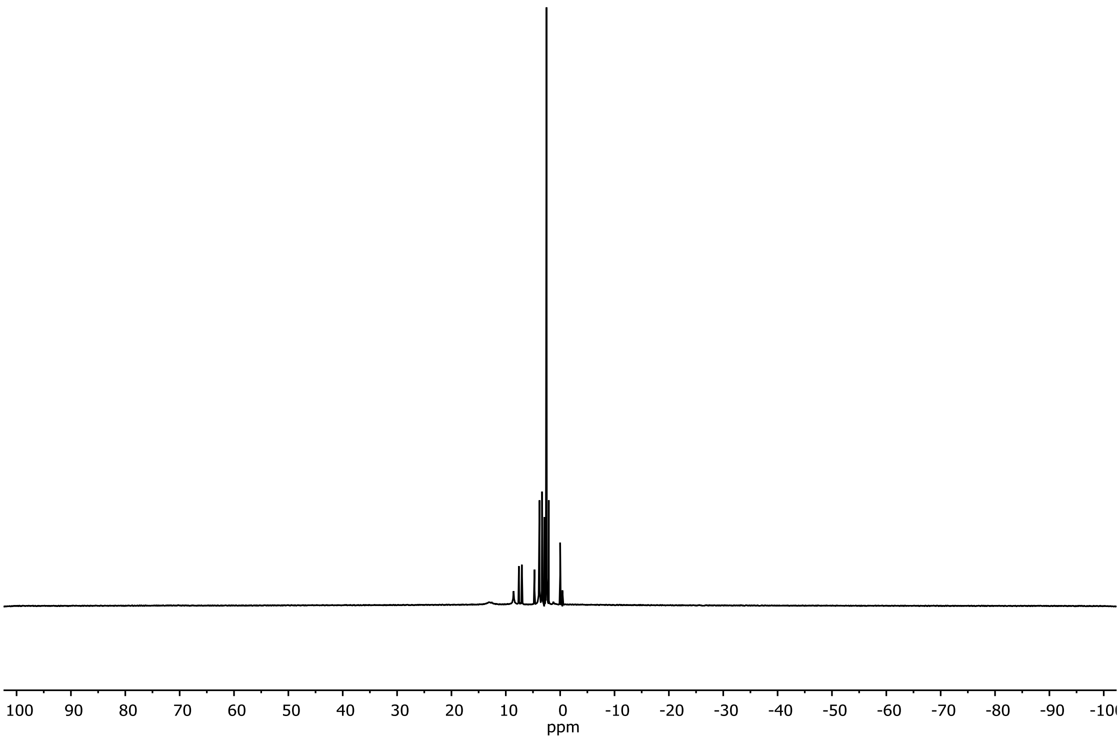


**Figure S20:** Evans $^{1}$H-NMR (400 MHz) of **C3** in DMSO-$d_6$ between -100 and 100 ppm

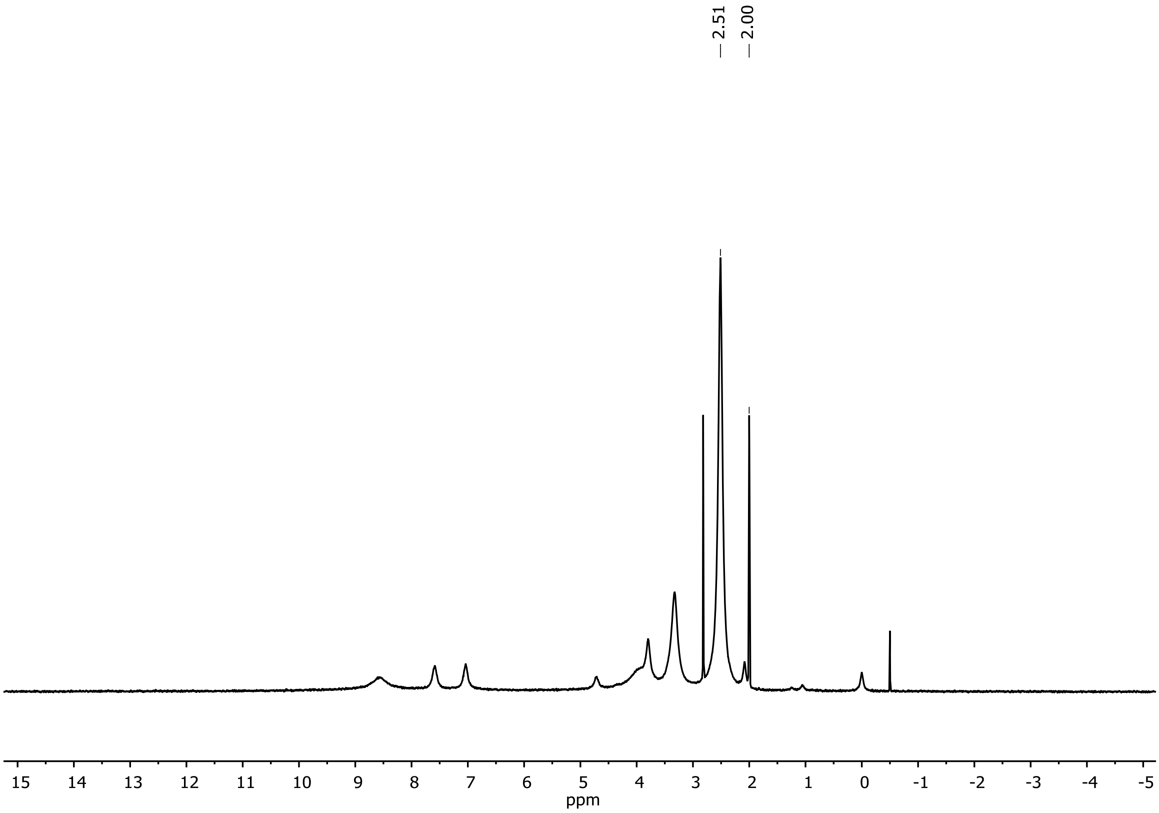


**Figure S21:** Evans $^{1}$H-NMR (400 MHz) of **C4** in DMSO-$d_6$

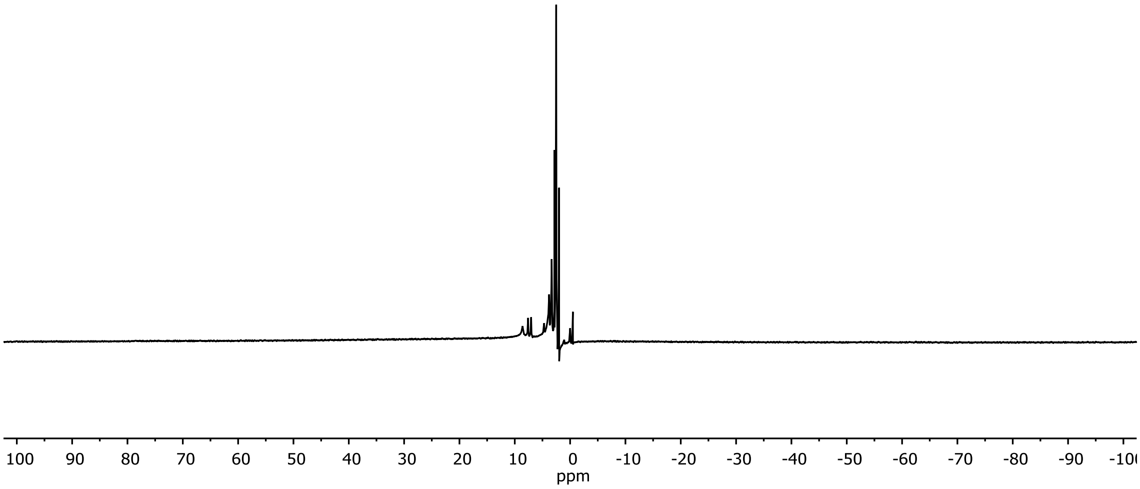


**Figure S22:** Evans $^{1}$H-NMR (400 MHz) of **C4** in DMSO-$d_6$ between -100 and 100 ppm

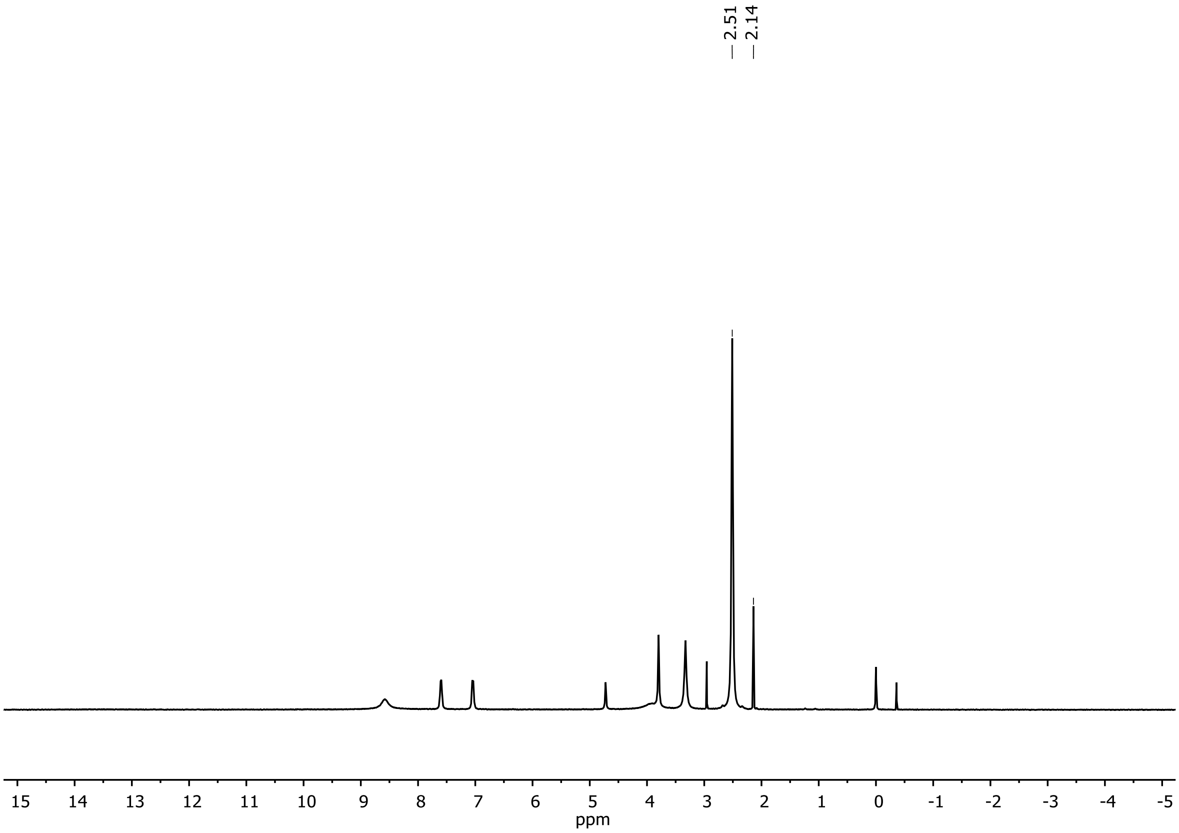


**Figure S23:** Evans $^{1}$H-NMR (400 MHz) of **C5** in DMSO-$d_6$

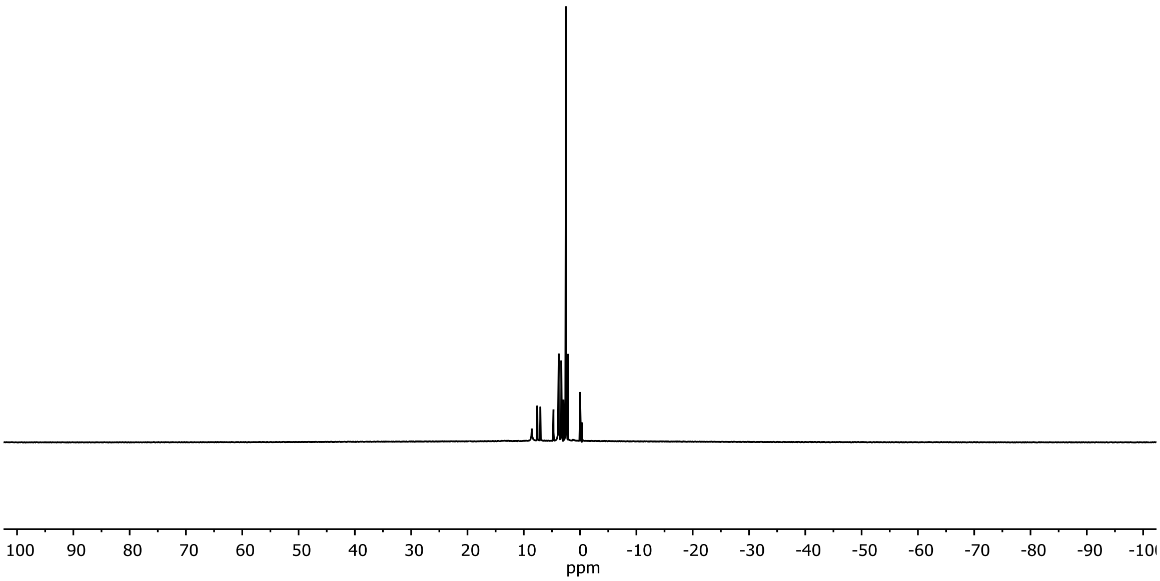


**Figure S24:** Evans $^{1}$H-NMR (400 MHz) of **C5** in DMSO-$d_6$ between -100 and 100 ppm

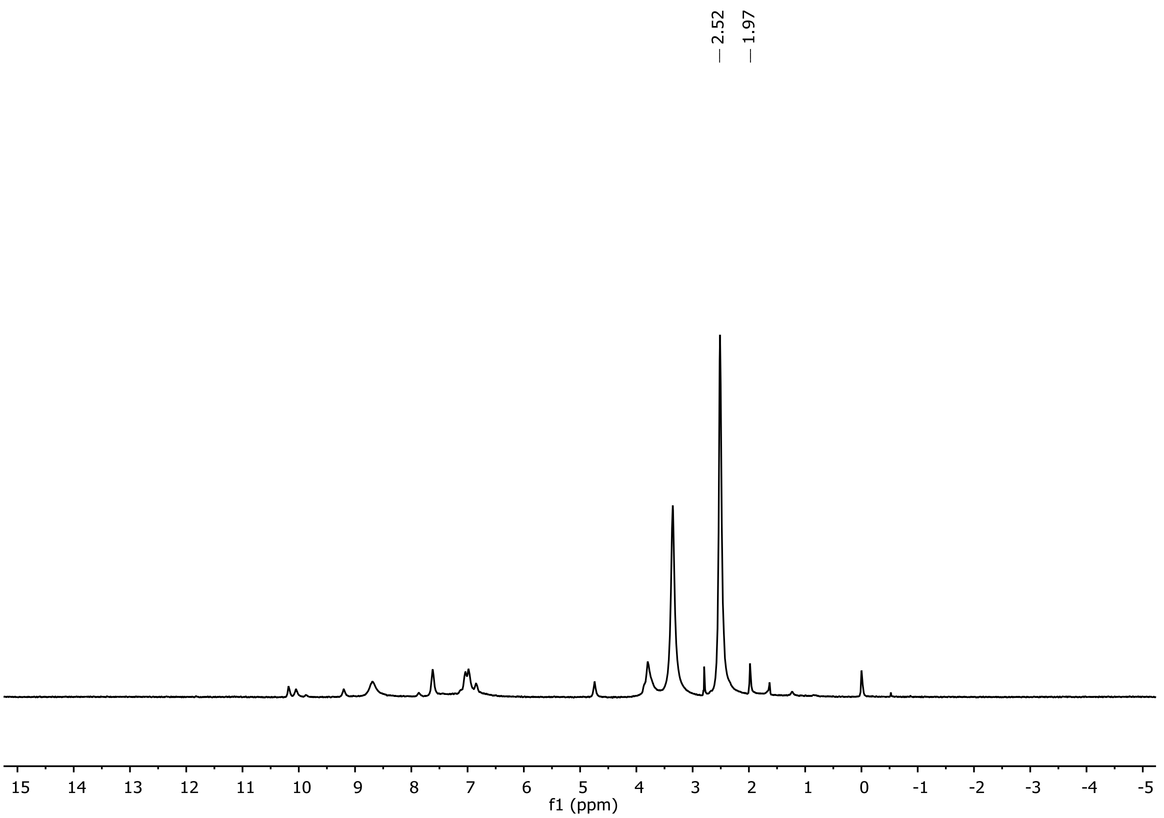


**Figure S25:** Evans $^{1}$H-NMR (400 MHz) of **C6** in DMSO-$d_6$

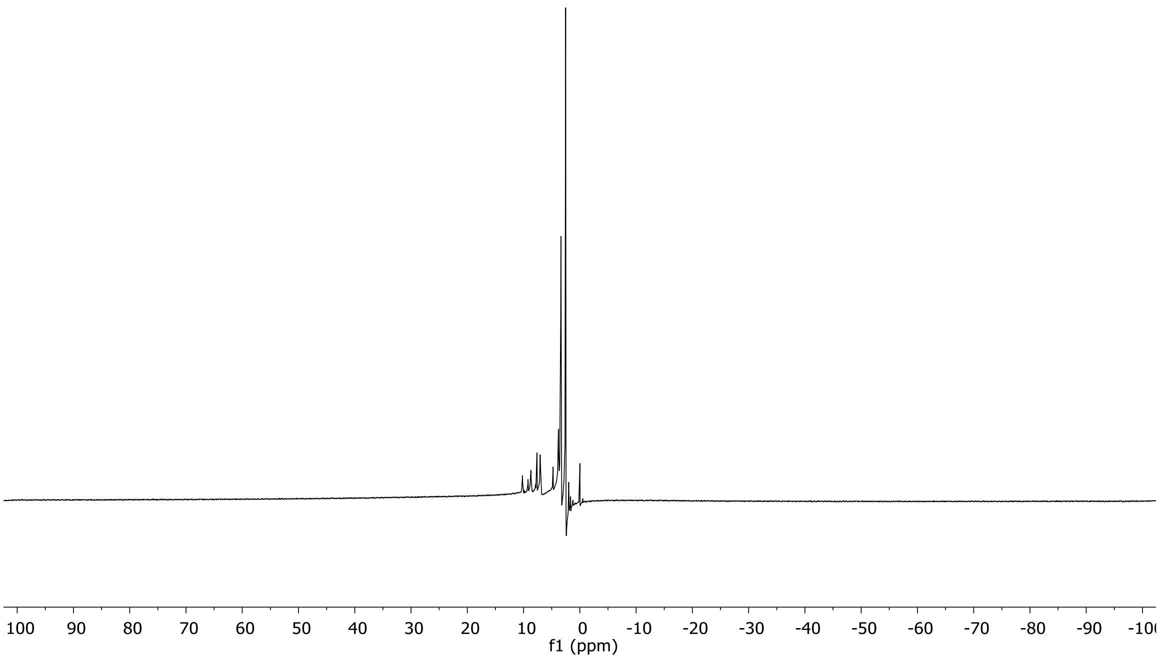


**Figure S26:** Evans $^{1}$H-NMR (400 MHz) of **C6** in DMSO-$d_6$ between -100 and 100 ppm

### 1.2.4 EPR spectra

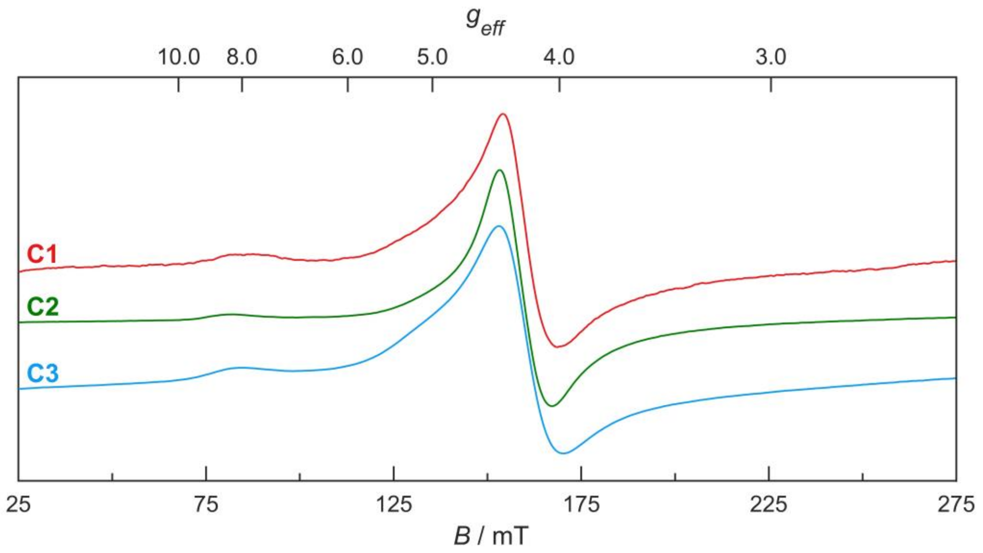


**Figure S27:** EPR spectrum of **C1**, **C2** and **C3** in DMSO at 298 K.

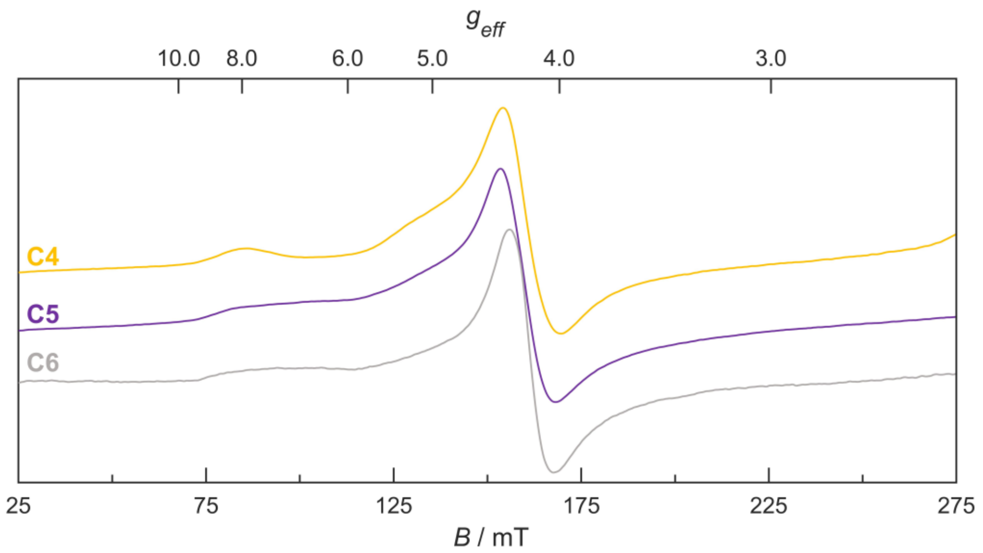


**Figure S28:** EPR spectrum of **C4**, **C5** and **C6** in DMSO at 298 K.

### 1.2.5 Crystallographic data of **C2** and **C4**

**Table S1:** Crystal data and structure refinement for **C2**

| | | |
|---|---|---|
| Empirical formula | $C_{32} H_{30} Cl Fe N_2 O_6$ | |
| Formula weight | 629.88 | |
| Temperature | 173.00 K | |
| Wavelength | 0.71073 Å | |
| Crystal system | Triclinic | |
| Space group | P-1 (no. 2) | |
| Unit cell dimensions | a = 9.5071(4) Å | α = 84.215(2)°. |
| | b = 10.9827(5) Å | β = 77.7820(10)°. |
| | c = 14.9725(6) Å | γ = 71.3800(10)°. |
| Volume | 1447.02(11) $Å^3$ | |
| Z | 2 | |
| Density (calculated) | 1.446 $Mg/m^3$ | |
| Absorption coefficient | 0.662 $mm^{-1}$ | |
| F(000) | 654 | |
| Crystal size | 0.32 x 0.16 x 0.08 $mm^3$ | |
| Theta range for data collection | 1.958 to 28.377°. | |
| Index ranges | -12<=h<=12, -14<=k<=14, -19<=l<=20 | |
| Reflections collected | 75964 | |
| Independent reflections | 7219 [R(int) = 0.0377] | |
| Completeness to theta = 25.242° | 99.9 % | |
| Absorption correction | Semi-empirical from equivalents | |
| Max. and min. transmission | 0.8496 and 0.7458 | |
| Refinement method | Full-matrix least-squares on $F^2$ | |
| Data / restraints / parameters | 7219 / 0 / 383 | |
| Goodness-of-fit on $F^2$ | 1.047 | |
| Final R indices [I>2sigma(I)] | R1 = 0.0295, wR2 = 0.0800 | |
| R indices (all data) | R1 = 0.0317, wR2 = 0.0823 | |
| Extinction coefficient | n/a | |
| Largest diff. peak and hole | 0.394 and -0.301 e.$Å^{-3}$ | |

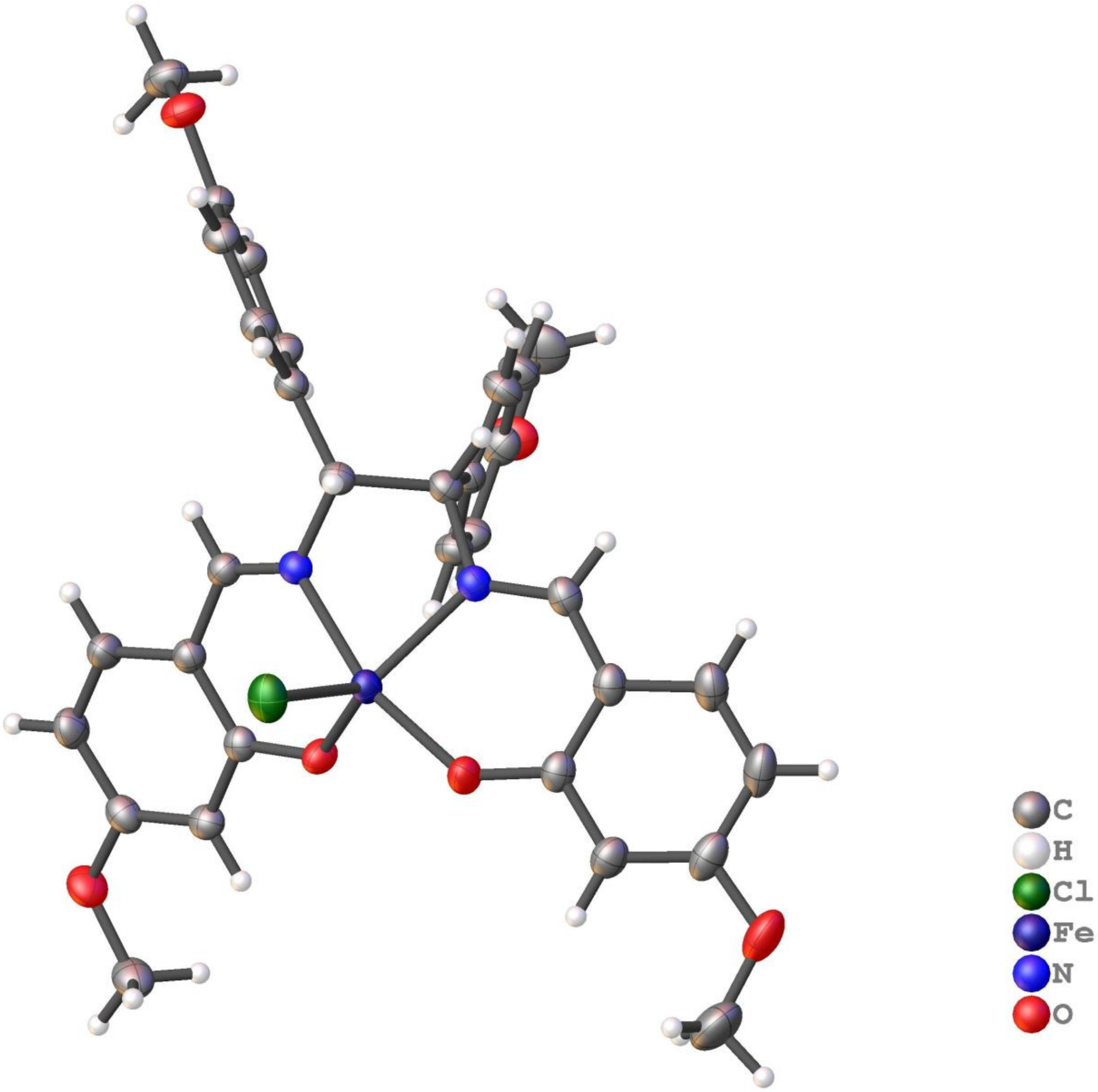


**Figure S29:** ORTEP of **C2** (PDP ID 2300982)

**Table S2:** Crystal data and structure refinement for **C4**

| | | |
|---|---|---|
| Empirical formula | $C_{30} H_{26} Cl Fe N_2 O_6$ | |
| Formula weight | 601.83 | |
| Temperature | 153.00 K | |
| Wavelength | 0.71073 Å | |
| Crystal system | Triclinic | |
| Space group | P-1(no. 2) | |
| Unit cell dimensions | a = 9.331(4) Å | α = 88.947(14)°. |
| | b = 10.765(5) Å | β = 79.702(15)°. |
| | c = 13.967(8) Å | γ = 69.113(15)°. |
| Volume | 1288.2(11) $Å^3$ | |
| Z | 2 | |
| Density (calculated) | 1.552 $Mg/m^3$ | |
| Absorption coefficient | 0.740 $mm^{-1}$ | |
| F(000) | 622 | |
| Crystal size | 0.16 x 0.06 x 0.04 $mm^3$ | |
| Theta range for data collection | 2.027 to 25.418°. | |
| Index ranges | -11<=h<=11, -12<=k<=12, -16<=l<=16 | |
| Reflections collected | 28884 | |
| Independent reflections | 4741 [R(int) = 0.0619] | |
| Completeness to theta = 25.242° | 100.0 % | |
| Absorption correction | Semi-empirical from equivalents | |
| Max. and min. transmission | 0.9582 and 0.8422 | |
| Refinement method | Full-matrix least-squares on $F^2$ | |
| Data / restraints / parameters | 4741 / 2 / 371 | |
| Goodness-of-fit on $F^2$ | 1.017 | |
| Final R indices [I>2sigma(I)] | R1 = 0.0428, wR2 = 0.1081 | |
| R indices (all data) | R1 = 0.0610, wR2 = 0.1193 | |
| Extinction coefficient | n/a | |
| Largest diff. peak and hole | 0.427 and -0.507 e.$Å^{-3}$ | |

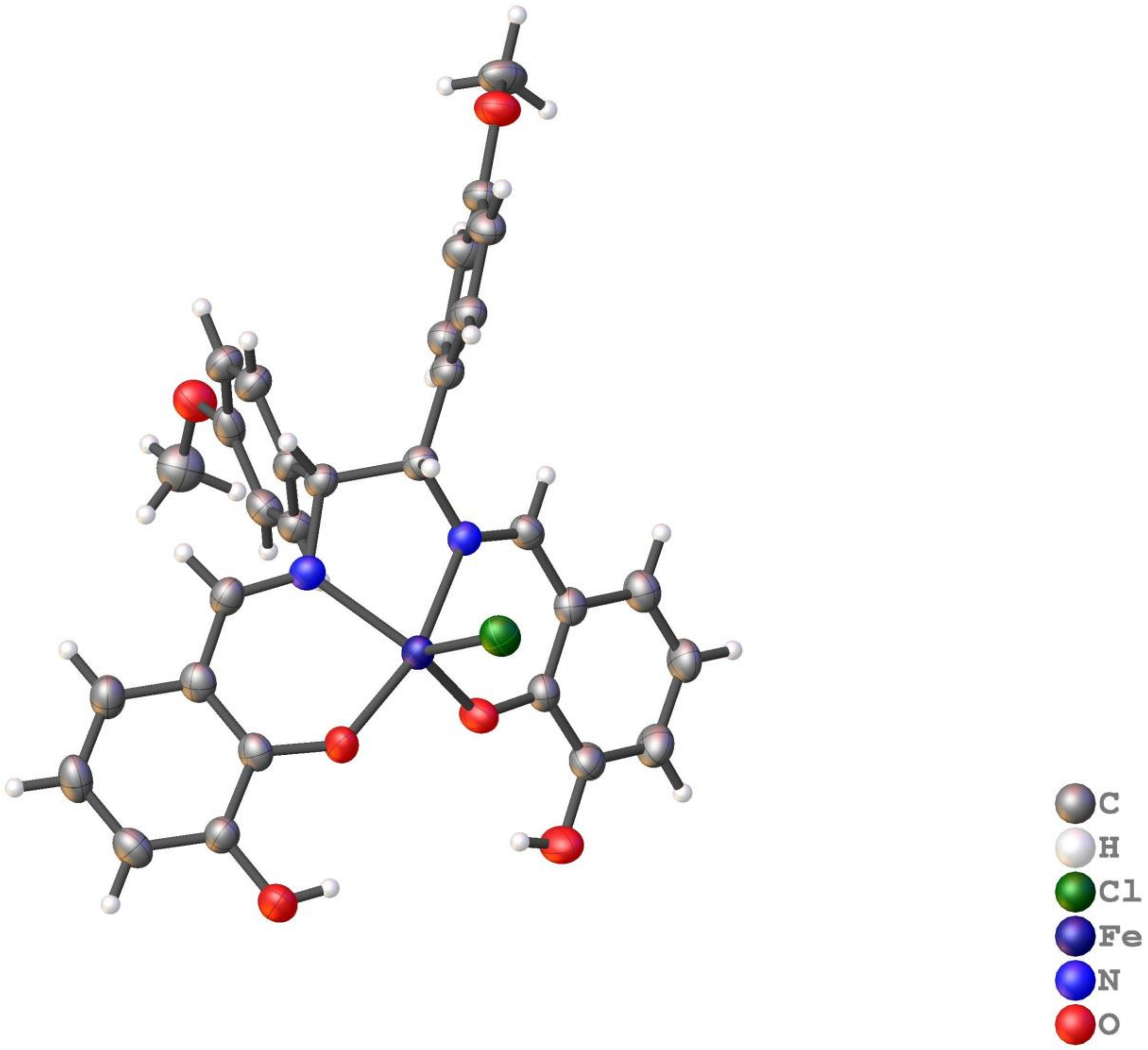


**Figure S30:** ORTEP of **C4** (PDP ID 2300983)

## 1.2.6 Cyclic voltammetry

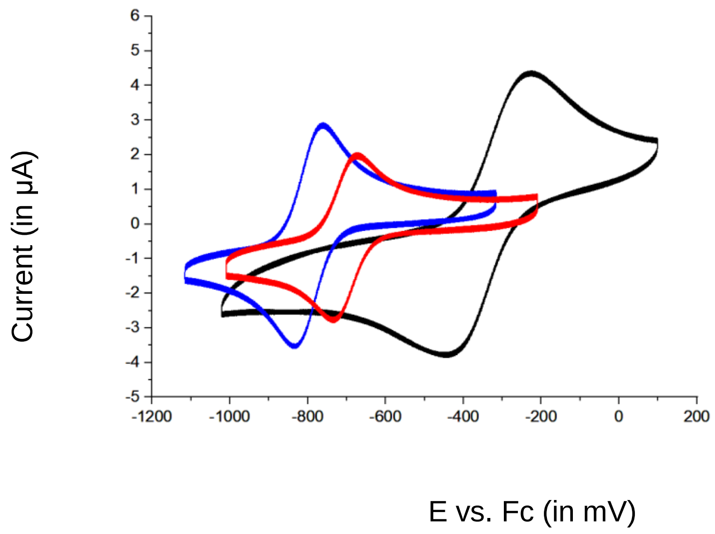


**Figure S31:** Overview of cyclic voltammograms of **C4** (red), **C5** (blue), **C6** (black) in DMSO

**Table S3:** Standard potential of **C1 – C6**, Ref. 1 and Ref. 2 in DMSO

| Compound | E vs Fc. |
|---|---|
| **Ref. 1** | -764 mV |
| **Ref. 2** | -803 mV |
| | |
| **C1** | -712 mV |
| **C2** | -754 mV |
| **C3** | -757 mV |
| **C4** | -694 mV |
| **C5** | -794 mV |
| **C6** | -333 mV |

# 2. Biological activity

## 2.1 Proliferation

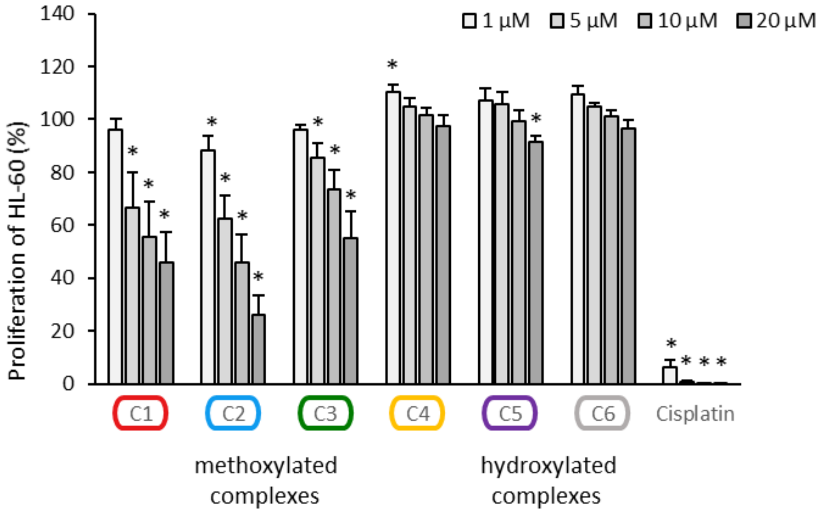


**Figure S32:** Proliferation of HL-60 treated for 72 h with the complexes **C1** – **C6** and cisplatin at concentrations 1 µM, 5 µM, 10 µM and 20 µM, respectively. Proliferation in the absence of the compounds was set at 100% (data not shown). Data are expressed as mean + SE of five experiments. * p<0.05 against no compound.

## 2.2 Metabolic activity

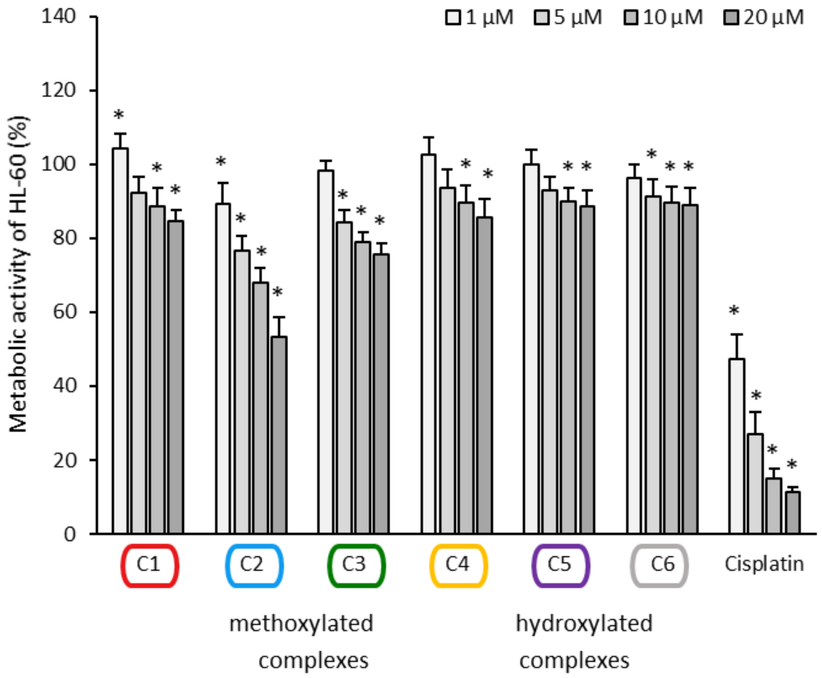


**Figure S33:** Metabolic activity of HL-60 treated for 72 h with the complexes **C1** – **C6** and cisplatin at concentrations 1 µM, 5 µM, 10 µM and 20 µM, respectively. Metabolic activity in the absence of the compounds was set at 100% (data not shown). Data are expressed as mean + SE of five experiments. * p<0.05 against no compound.

## 2.3 Scratch assay

Scratch assay without complex (Figure S34A), with 20 µM C2 (Figure S34B) and 20 µM **C5** (Figure S34C) analysed immediately after addition of compound (0 h) and after 24 h and 72 h, respectively. One representative experiment is shown.

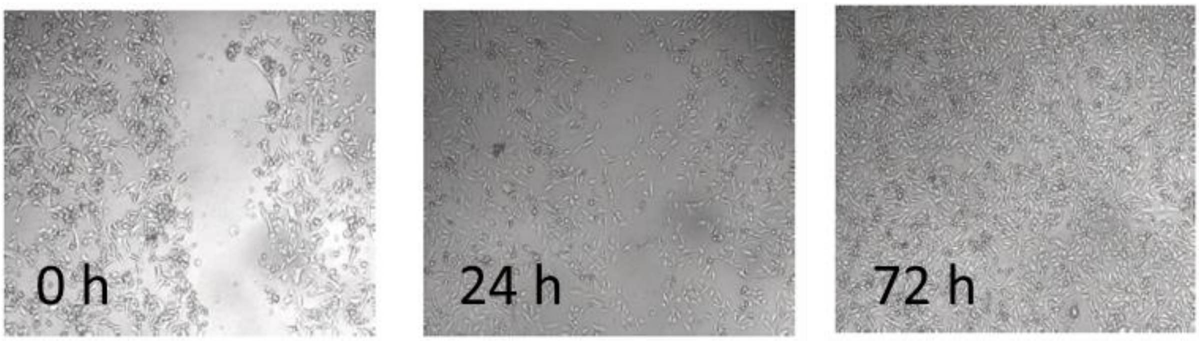


**Figure S34A:** MDA-MB 231

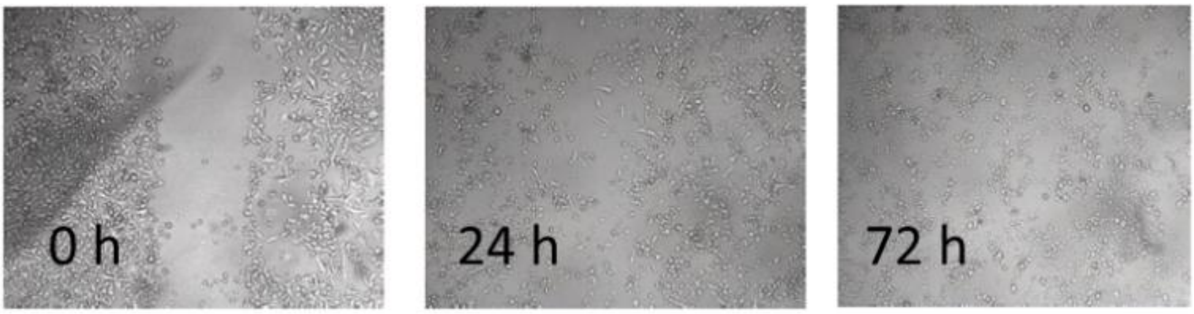


**Figure S34B:** 20 µM **C2**

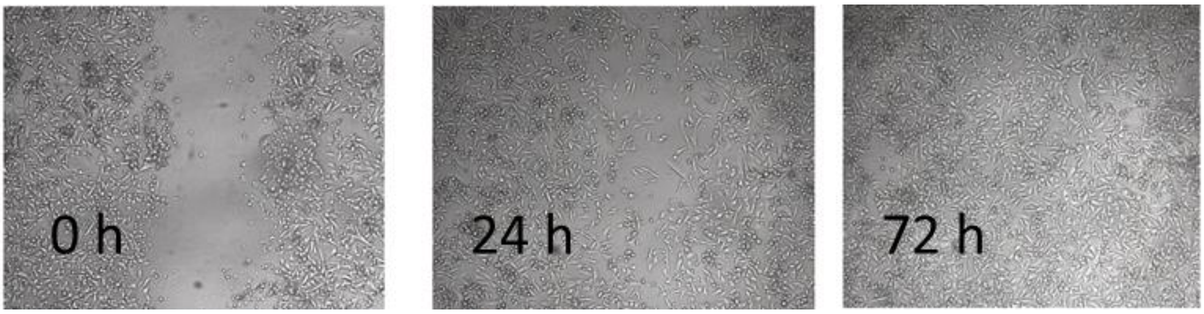


**Figure S34C:** 20 µM **C5**

## 2.4 Live confocal microscopy

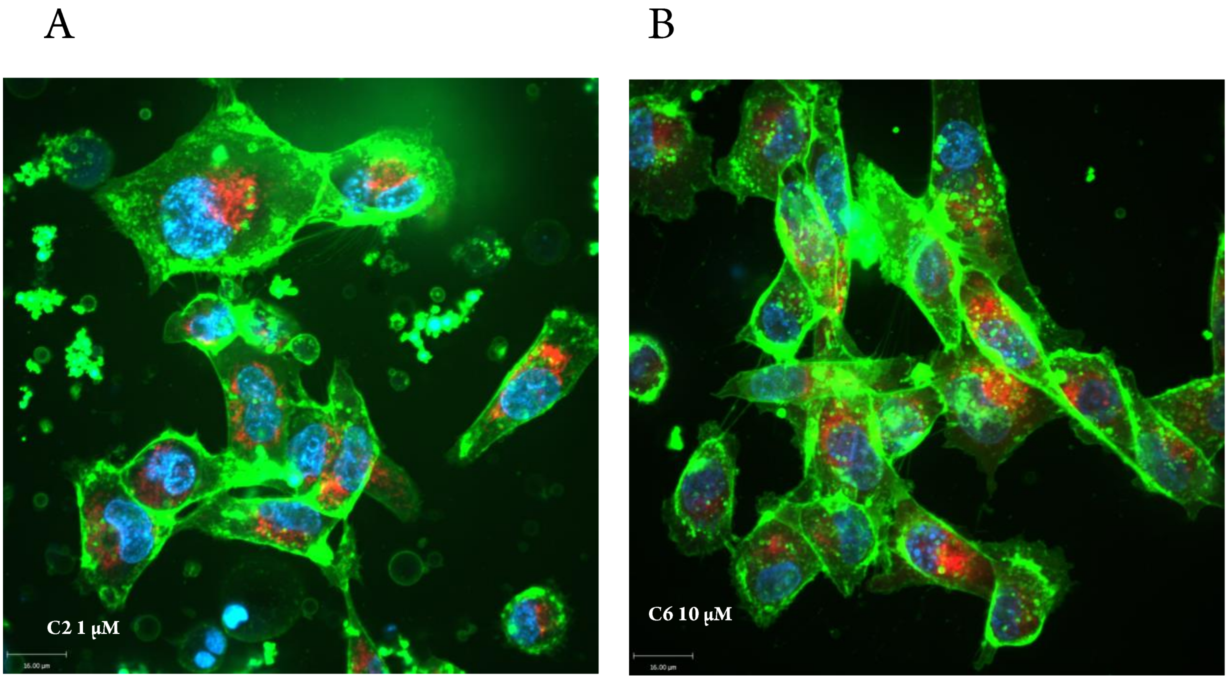


**Figure S35:** MDA-MB 231 cells imaged by live confocal microscopy after staining with Hoechst 33342 (blue, 405 nm) to image the nuclei, wheat germ lectin (green, 488 nm) to visualize the cell morphology and tetramethylrhodamine ethyl ester (TMRM, red, 561 nm) to stain active mitochondria. **S35A**: cells treated with 1 µM **C2** and **S35B** cells treated with 10 µM **C6** for 24 h. One representative experiment is shown.
