# Supplementary material for: Design, Synthesis, Electrochemical, and Biological Evaluation of Fluorescent Chlorido[N,N′-bis(methoxy/hydroxy)salicylidene-1,2-bis(4-methoxyphenyl)ethylenediamine]iron(III) Complexes as Anticancer Agents
Source: J Med Chem. 2023 Nov 28;66(23):15916–25. doi: 10.1021/acs.jmedchem.3c01359 (PMC10726350; doi:10.1021/acs.jmedchem.3c01359)

checkCIF/PLATON report

Structure factors have been supplied for datablock(s) C2, C4

THIS REPORT IS FOR GUIDANCE ONLY. IF USED AS PART OF A REVIEW PROCEDURE FOR PUBLICATION, IT SHOULD NOT REPLACE THE EXPERTISE OF AN EXPERIENCED CRYSTALLOGRAPHIC REFEREE.

No syntax errors found. CIF dictionary Interpreting this report

Datablock: C2

Bond precision: C-C = 0.0019 A Wavelength=0.71073

Cell:	a=9.5071(4)	b=10.9827(5)	c=14.9725(6)
	alpha=84.215(2)	beta=77.782(1)	gamma=71.380(1)
Temperature:	173 K		

	Calculated	Reported
Volume	1447.02(11)	1447.02(11)
Space group	P -1	P -1
Hall group	-P 1	-P 1
Moiety formula	C32 H30 Cl Fe N2 O6	C32 H30 Cl Fe N2 O6
Sum formula	C32 H30 Cl Fe N2 O6	C32 H30 Cl Fe N2 O6
Mr	629.88	629.88
Dx,g cm-3	1.446	1.446
Z	2	2
Mu (mm-1)	0.662	0.662
F000	654.0	654.0
F000'	655.25	
h,k,lmax	12,14,20	12,14,20
Nref	7249	7219
Tmin,Tmax	0.881,0.948	0.746,0.850
Tmin'	0.809	

Correction method= # Reported T Limits: Tmin=0.746 Tmax=0.850
AbsCorr = MULTI-SCAN

Data completeness= 0.996 Theta(max)= 28.377

R(reflections)= 0.0295(6756) wR2(reflections)= 0.0823(7219)

S = 1.047 Npar= 383

The following ALERTS were generated. Each ALERT has the format
test-name_ALERT_alert-type_alert-level.
Click on the hyperlinks for more details of the test.

Alert level C

```
PLAT911_ALERT_3_C Missing FCF Refl Between Thmin & STh/L=    0.600          5 Report
```

Alert level G

```
PLAT232_ALERT_2_G Hirshfeld Test Diff (M-X)  Fe1      --N1       .        6.2 s.u.
PLAT232_ALERT_2_G Hirshfeld Test Diff (M-X)  Fe1      --N2       .        5.6 s.u.
PLAT794_ALERT_5_G Tentative Bond Valency for Fe1       (III)     .       3.00 Info
PLAT883_ALERT_1_G No Info/Value for _atom_sites_solution_primary .     Please Do !
PLAT910_ALERT_3_G Missing # of FCF Reflection(s) Below Theta(Min).          1 Note
PLAT912_ALERT_4_G Missing # of FCF Reflections Above STh/L=  0.600         24 Note
PLAT978_ALERT_2_G Number C-C Bonds with Positive Residual Density.         20 Info
```

0 **ALERT level A** = Most likely a serious problem - resolve or explain
0 **ALERT level B** = A potentially serious problem, consider carefully
1 **ALERT level C** = Check. Ensure it is not caused by an omission or oversight
7 **ALERT level G** = General information/check it is not something unexpected

1 ALERT type 1 CIF construction/syntax error, inconsistent or missing data
3 ALERT type 2 Indicator that the structure model may be wrong or deficient
2 ALERT type 3 Indicator that the structure quality may be low
1 ALERT type 4 Improvement, methodology, query or suggestion
1 ALERT type 5 Informative message, check

Datablock: C4

Bond precision: C-C = 0.0043 A Wavelength=0.71073

Cell:	a=9.331(4)	b=10.765(5)	c=13.967(8)
	alpha=88.947(14)	beta=79.702(15)	gamma=69.113(15)
Temperature:	153 K		

	Calculated	Reported
Volume	1288.1(11)	1288.2(11)
Space group	P -1	P -1
Hall group	-P 1	-P 1
Moiety formula	C30 H26 Cl Fe N2 O6	C30 H26 Cl Fe N2 O6
Sum formula	C30 H26 Cl Fe N2 O6	C30 H26 Cl Fe N2 O6
Mr	601.83	601.83
Dx,g cm-3	1.552	1.552
Z	2	2
Mu (mm-1)	0.740	0.740
F000	622.0	622.0
F000'	623.25	
h,k,lmax	11,13,16	11,12,16
Nref	4754	4741
Tmin,Tmax	0.948,0.971	0.842,0.958
Tmin'	0.888	

Correction method= # Reported T Limits: Tmin=0.842 Tmax=0.958
AbsCorr = MULTI-SCAN

Data completeness= 0.997 Theta(max)= 25.418

R(reflections)= 0.0428(3650) wR2(reflections)= 0.1193(4741)

S = 1.017 Npar= 371

The following ALERTS were generated. Each ALERT has the format
test-name_ALERT_alert-type_alert-level.
Click on the hyperlinks for more details of the test.

Alert level G

```
PLAT002_ALERT_2_G Number of Distance or Angle Restraints on AtSite          4 Note
PLAT172_ALERT_4_G The CIF-Embedded .res File Contains DFIX Records          1 Report
PLAT432_ALERT_2_G Short Inter X...Y Contact  O5       ..C1       .       3.01 Ang.
                                           2-x,-y,1-z  =      2_756 Check
PLAT794_ALERT_5_G Tentative Bond Valency for Fe1       (III)     .       3.01 Info
PLAT860_ALERT_3_G Number of Least-Squares Restraints ............          2 Note
PLAT883_ALERT_1_G No Info/Value for _atom_sites_solution_primary .     Please Do !
PLAT910_ALERT_3_G Missing # of FCF Reflection(s) Below Theta(Min).          1 Note
PLAT912_ALERT_4_G Missing # of FCF Reflections Above STh/L=  0.600         12 Note
PLAT978_ALERT_2_G Number C-C Bonds with Positive Residual Density.          5 Info
```

---

0 **ALERT level A** = Most likely a serious problem - resolve or explain
0 **ALERT level B** = A potentially serious problem, consider carefully
0 **ALERT level C** = Check. Ensure it is not caused by an omission or oversight
9 **ALERT level G** = General information/check it is not something unexpected

```
  1 ALERT type 1 CIF construction/syntax error, inconsistent or missing data
  3 ALERT type 2 Indicator that the structure model may be wrong or deficient
  2 ALERT type 3 Indicator that the structure quality may be low
  2 ALERT type 4 Improvement, methodology, query or suggestion
  1 ALERT type 5 Informative message, check
```

It is advisable to attempt to resolve as many as possible of the alerts in all categories. Often the minor alerts point to easily fixed oversights, errors and omissions in your CIF or refinement strategy, so attention to these fine details can be worthwhile. In order to resolve some of the more serious problems it may be necessary to carry out additional measurements or structure refinements. However, the purpose of your study may justify the reported deviations and the more serious of these should normally be commented upon in the discussion or experimental section of a paper or in the "special_details" fields of the CIF. checkCIF was carefully designed to identify outliers and unusual parameters, but every test has its limitations and alerts that are not important in a particular case may appear. Conversely, the absence of alerts does not guarantee there are no aspects of the results needing attention. It is up to the individual to critically assess their own results and, if necessary, seek expert advice.

Publication of your CIF in IUCr journals

A basic structural check has been run on your CIF. These basic checks will be run on all CIFs submitted for publication in IUCr journals (*Acta Crystallographica*, *Journal of Applied Crystallography*, *Journal of Synchrotron Radiation*); however, if you intend to submit to *Acta Crystallographica Section C* or *E* or *IUCrData*, you should make sure that full publication checks are run on the final version of your CIF prior to submission.

Publication of your CIF in other journals

Please refer to the *Notes for Authors* of the relevant journal for any special instructions relating to CIF submission.

PLATON version of 06/07/2023; check.def file version of 30/06/2023

Datablock C2 - ellipsoid plot

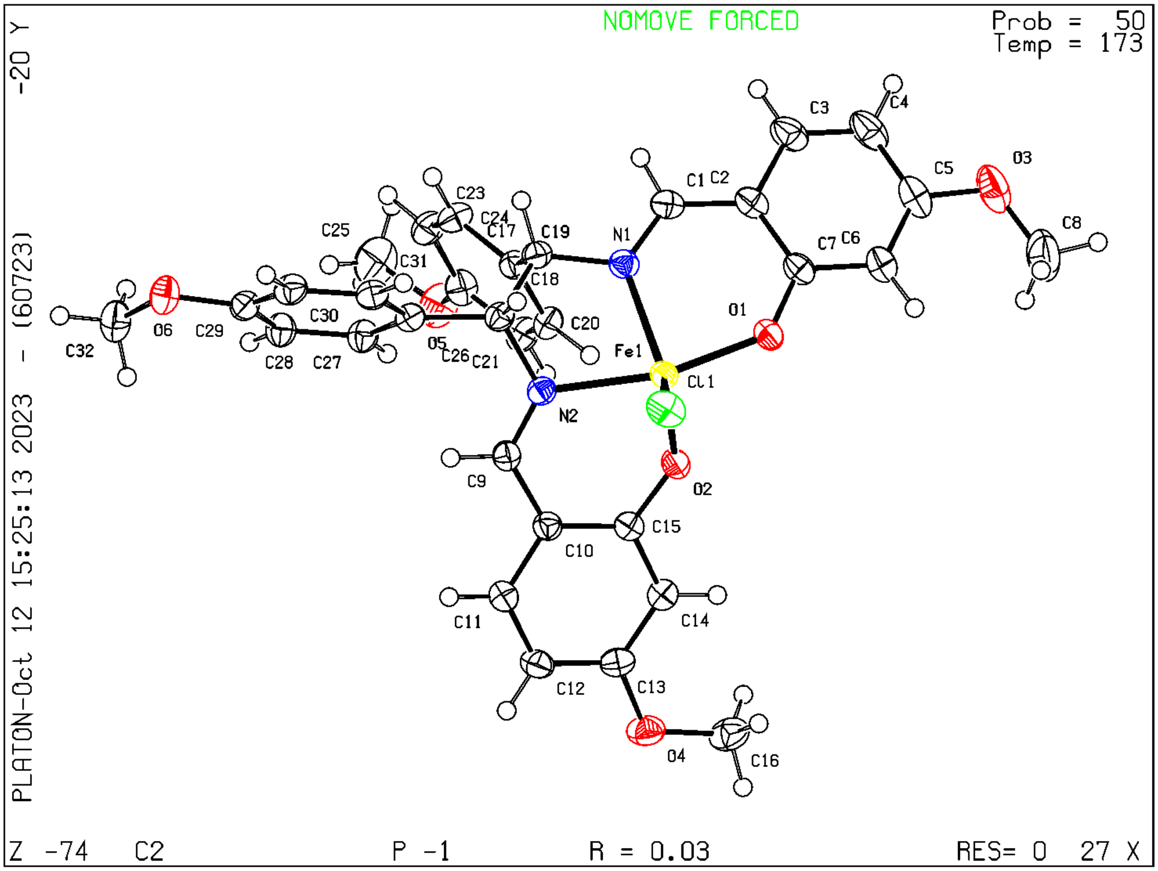

Datablock C4 - ellipsoid plot

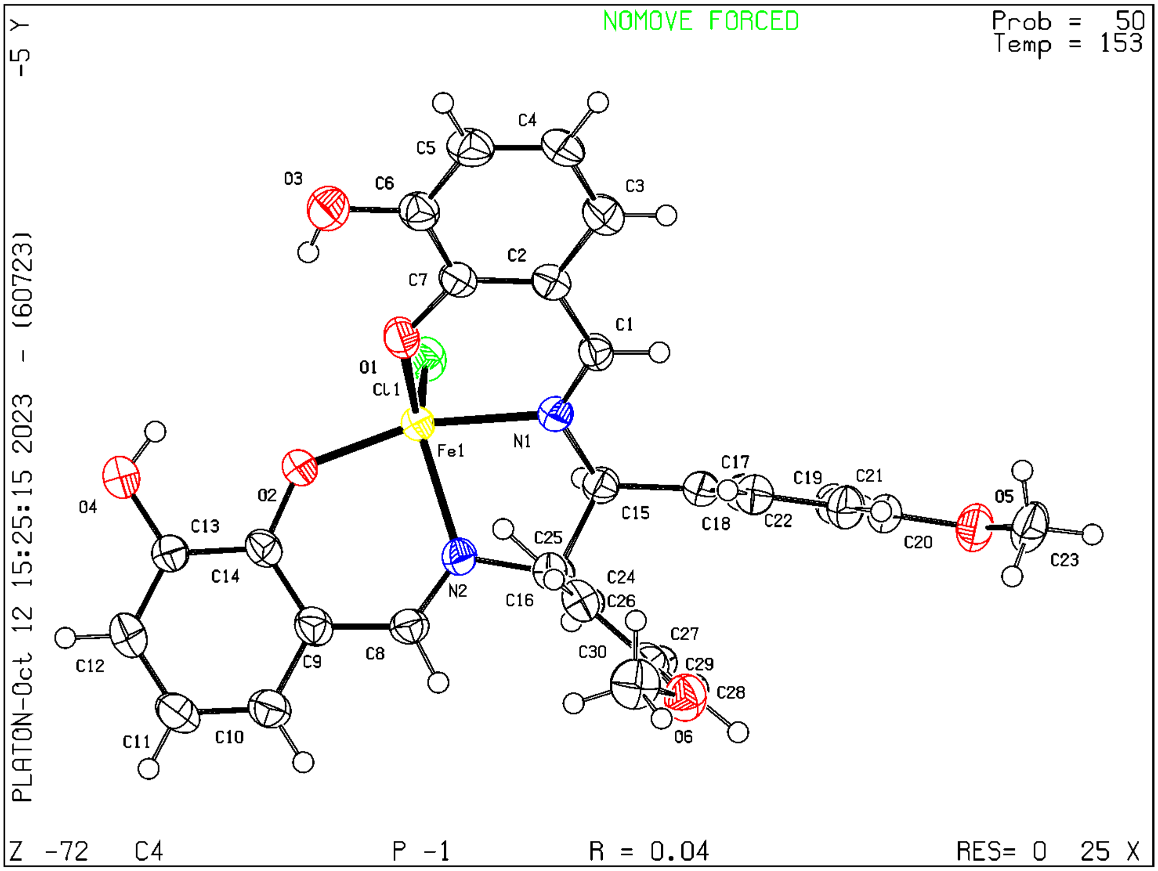

Supplement: Supplementary file 3 — jm3c01359_si_003.pdf [file jm3c01359_si_003.pdf]
